# Insight in Genome-Wide Association of Metabolite Quantitative Traits by Exome Sequence Analyses

**DOI:** 10.1371/journal.pgen.1004835

**Published:** 2015-01-08

**Authors:** Ayşe Demirkan, Peter Henneman, Aswin Verhoeven, Harish Dharuri, Najaf Amin, Jan Bert van Klinken, Lennart C. Karssen, Boukje de Vries, Axel Meissner, Sibel Göraler, Arn M. J. M. van den Maagdenberg, André M. Deelder, Peter A. C ’t Hoen, Cornelia M. van Duijn, Ko Willems van Dijk

**Affiliations:** 1 Department of Human Genetics, Leiden University Medical Center, Leiden, the Netherlands; 2 Department of Epidemiology, Erasmus Medical Center, Rotterdam, the Netherlands; 3 Center for Proteomics and Metabolomics, Leiden University Medical Center, Leiden, the Netherlands; 4 Department of Neurology, Leiden University Medical Center, Leiden, the Netherlands; 5 Department of Endocrinology, Leiden University Medical Center, Leiden, the Netherlands; University of Alabama at Birmingham, United States of America

## Abstract

Metabolite quantitative traits carry great promise for epidemiological studies, and their genetic background has been addressed using Genome-Wide Association Studies (GWAS). Thus far, the role of less common variants has not been exhaustively studied. Here, we set out a GWAS for metabolite quantitative traits in serum, followed by exome sequence analysis to zoom in on putative causal variants in the associated genes. ^1^H Nuclear Magnetic Resonance (^1^H-NMR) spectroscopy experiments yielded successful quantification of 42 unique metabolites in 2,482 individuals from The Erasmus Rucphen Family (ERF) study. Heritability of metabolites were estimated by SOLAR. GWAS was performed by linear mixed models, using HapMap imputations. Based on physical vicinity and pathway analyses, candidate genes were screened for coding region variation using exome sequence data. Heritability estimates for metabolites ranged between 10% and 52%. GWAS replicated three known loci in the metabolome wide significance: *CPS1* with glycine (P-value  = 1.27×10^−32^), *PRODH* with proline (P-value  = 1.11×10^−19^), *SLC16A9* with carnitine level (P-value  = 4.81×10^−14^) and uncovered a novel association between *DMGDH* and dimethyl-glycine (P-value  = 1.65×10^−19^) level. In addition, we found three novel, suggestively significant loci: *TNP1* with pyruvate (P-value  = 1.26×10^−8^), *KCNJ16* with 3-hydroxybutyrate (P-value  = 1.65×10^−8^) and 2p12 locus with valine (P-value  = 3.49×10^−8^). Exome sequence analysis identified potentially causal coding and regulatory variants located in the genes *CPS1, KCNJ2* and *PRODH*, and revealed allelic heterogeneity for *CPS1* and *PRODH.* Combined GWAS and exome analyses of metabolites detected by high-resolution ^1^H-NMR is a robust approach to uncover metabolite quantitative trait loci (mQTL), and the likely causative variants in these loci. It is anticipated that insight in the genetics of intermediate phenotypes will provide additional insight into the genetics of complex traits.

## Author Summary

Human metabolic individuality is under strict control of genetic and environmental factors. In our study, we aimed to find the genetic determinants of circulating molecules in sera of large set of individuals representing the general population. First, we performed a hypothesis-free genome wide screen in this population to identify genetic regions of interest. Our study confirmed four known gene metabolite connections, but also pointed to four novel ones. Genome-wide screens enriched for common intergenic variants may miss causal genetic variations directly changing the protein sequence. To investigate this further, we zoomed into regions of interest and tested whether the association signals obtained in the first stage were direct, or whether they represent causal variations, which were not captured in the initial panel. These subsequent tests showed that protein coding and regulatory variations are involved in metabolite levels. For two genomic regions we also found that genes harbour more than one causal variant influencing metabolite levels independent of each other. We also observed strong connection between markers of cardio-metabolic health and metabolites. Taken together, our novel loci are of interest for further research to investigate the causal relation to for instance type 2 diabetes and cardiovascular disease.

## Introduction

Intermediary metabolites in bodily fluids seem a direct reflection of our genetic constituency in interaction with the environment, which includes eating habits, life style and other external factors. Thus, the use of metabolomic phenotypes in genetic epidemiological studies may provide specific insight in pathways underlying complex metabolic diseases, such as type 2 diabetes mellitus (T2D), stroke or cardiovascular disease (CVD) but also other complex diseases such as rheumatoid arthritis, migraine and depression [1]–[3]. The sample sizes in the first genome-wide association studies (GWAS) of metabolite quantitative traits were in general relatively small compared to GWAS on traditional phenotypes, yet revealed strong signals for association of common variants with specific metabolites. Single-proton Nuclear Magnetic Resonance (^1^H-NMR) spectroscopy is a metabolomics technique that requires relatively little sample preparation, yet has the capacity to reproducibly quantify dozens to more than 100 metabolite signals per measurement. Several studies have reported genetic loci that influence the metabolites quantified by ^1^H-NMR in plasma and urine [4]-[7]. Here, we present the results of 42 plasma metabolites quantified by ^1^H-NMR spectroscopy in 2,482 individuals of the family-based Erasmus Rucphen Family (ERF) study, a Dutch genetic isolate. We estimated the heritability and the effect of shared environment (household effect) for these metabolites. The GWA was followed by high-resolution analysis of coding variants in the candidate genes that were identified by physical proximity and pathway analysis. To provide further insight into the pathogenesis of cardio-metabolic diseases, we also investigated the association between the NMR metabolites and the classical risk factors for CVD and T2D.

## Results

### Heritability estimates and GWAS results

**Figure 1:**
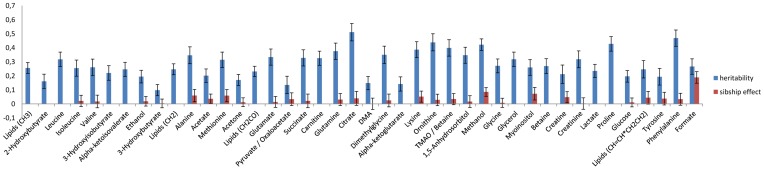
Heritability and sibship effects on the NMR metabolites. Figure shows the magnitude of heritability (H^2^) and sibship (household) effect estimates for each metabolic trait included in the ERF population.

**Figure 2:**
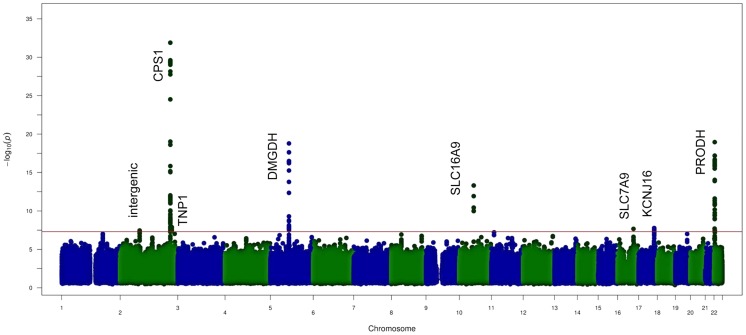
GWAS results of the NMR metabolites. Figure shows the aggregated Manhattan plot for the 42 metabolites studied. Red line shows the suggestive genome-wide significance level with a P-value of 5×10^−8^.Loci harbouring *DMGDH, SLC16A9, PRODH* and *CPS1* are reported as metabolome wide significance.

The study was conducted in the ERF population (see S1 Table) using fasting serum samples. After quality filtering, we resolved 42 metabolites, for which the identity was confirmed by the typical chemical shifts of the related peaks, their high correlation with other peaks and spiking of pure compounds in serum (S2 Table). Heritability estimates of the metabolites were moderate to high ranging from 10% to 52% whereas estimates for the shared environmental effect ranged from 0% to 8% (Fig. 1). The highest heritability is observed for citrate (52%), followed by phenylalanine (51%), ornithine (47%) and methanol (45%) whereas the lowest heritability estimate was 10% for 3-hydroxybutyrate. We performed genome-wide association (GWA) analysis for all metabolite SNP pairs, including 2.5 M SNPs from the HapMap2 reference panel, see S1 Fig. for the Q-Q plots of the 42 metabolites. In total, we found eight unique genomic loci that associated with NMR metabolites below the genome-wide significant P-value threshold (P-value <5.0×10^−8^) as shown in the Manhattan plot (Fig. 2). Regional plots of the 8 loci are shown in S2 Fig.. Four of these loci were also significant after correction for the number of metabolites analyzed (P-value <1.10×10^−9^) and three of these were previously shown to associate with the same metabolites: rs715 located in the 3′UTR of the carbamoyl-phosphate synthase 1 *(CPS1)* gene associated with glycine level (P-value  = 1.27×10^−32^) [8], rs2540641 35 Kb distant from proline dehydrogenase (oxidase) 1 (*PRODH)* gene (P-value  = 1.11×10^−19^) associated with proline levels [9] and rs1171614 in the 5’UTR of *SLC16A9* (solute carrier family 16, member 9) associated with carnitine level (P-value  = 4.81×10^−14^) [9]–[11]. The association between intronic SNP rs248386 within *DMGDH* (dimethyl-glycine dehydrogenase) and dimethyl-glycine level is a novel finding (P-value  = 1.65×10^−19^). This locus has also been associated with betaine, which is a closely related metabolite [8].

Four other suggestively significant loci were uncovered by our analyses (5.0×10^−8^>P-value>1.10×10^−9^). One of these has previously been identified in urine: the association between rs8056893 within the *SLC7A9* (solute carrier family 7 member 9) and lysine (P-value  = 1.26×10^−8^) [12]. Three novel associations were found (1) rs1922005 located inside the *TNP1* (transition protein 1) gene and pyruvate level (P-value  = 1.26×10^−8^), (2) rs9896573 located nearby *KCNJ16* (potassium inwardly-rectifying channel, subfamily J, member 16) and 3-hydroxybutyrate level (P-value  = 1.65×10^−8^) (3) rs11687765 located in a non-protein coding region on chromosome 2 and valine level, (P-value  = 3.49×10^−8^). For the 8 top loci, we also investigated the mode of inheritance. The model supervised analysis in those regions of interest shows that the recessive genetic model applies successfully for six of the effect alleles: rs715 (*CPS1*) on glycine, rs1922005 (*TNP1*) on pyruvate rs248386 (*DMGDH*) on dimethyl-glycine, rs1171614 (*SLC16A9*) on carnitine, rs8056893 (*SLC7A9*) on lysine and rs2540641 (*PRODH*) on proline. For rs11687765 (intergenic on chromosome 2) affecting valine, the mode of inheritance seems to be dominant for the effect allele. For rs9896573 (*KCNJ16*) affecting 3-hydroxybutyrate, the over-dominant model resulted in the strongest association among the models tested.

#### Fine mapping within the candidate genes

In the same study population exome sequences of 921 individuals from the ERF pedigree were analyzed for potentially causal SNPs in biologically plausible genes which were extracted using an automated workflow, within the top eight GWAS loci in Table 1. The outputs of the automated workflow are given in S1 Text. In addition to coding region variation, a number of intronic variants that were captured around the intron-exon connections, as well as nearby 5’UTR and 3′UTR variants were captured by sequencing and those were also included in analysis. This approach revealed in total seven independent SNPs with potentially causal effects located inside *CPS1, KCNJ2* (potassium inwardly-rectifying channel, subfamily J, member 2), *PRODH* and *SLC25A1* (solute carrier family 25 member 1) (Table 2). More precisely, for glycine we found evidence for two independent effects within the *CPS1* gene. First, the missense mutation Thr1412Asn (rs1047891) within *CPS1* is the most likely causal variant tagged by the GWAS SNP rs715, due to the high LD (R^2^  = 0.92) and large drop in P-values after conditioning the SNP-metabolite associations for each other. Second, we found three intronic variants in strong LD with each other (R^2^>0.89) in *CPS1* that independently associated with glycine (lowest P-value  = 2.55×10^−5^ for rs182548513, Table 2) when conditioned on the leading GWAS SNP. For 3-hydroxybutyrate, we found that rs173135 located 3′UTR of *KCNJ2* gene is most likely the causal SNP (P-value  = 1.01×10^−07^) influencing the circulating level of this metabolite. Rs173135 is in strong LD with the leading GWAS SNP (R^2^  = 0.72) showed a large drop in P-value, yet remained significant in the conditional analysis (P-value  = 0.002). For proline, in total, we observed four independent effects within the *PRODH* locus including one missense mutation Thr116Asn (rs5747933, P-value  = 1.82×10^−9^), two intronic SNPS (rs1076466, P-value  = 6.34×10^−4^ and rs3213491, P-value  = 7.48×10^−4^) and one (semi-)independent SNP rs13058335 (R^2^  = 0.66 with the leading GWAS SNP), explaining the GWAS finding with a conditional P-value  = 1.20×10^−5^. We also found significant coding variations associated with dimethyl-glycine, carnitine, pyruvate and lysine however all those signals vanished after adjustment by the leading GWAS SNP, indicating that these associations so far are best explained by the leading GWAS hits in these regions (S3 Table).

**Table 1:** Unique loci that influence the NMR-metabolome.

Metabolite	SNP	P-value	MAF	Location	CHR	Position	Candidate genes	R^2^	Genetic loci within +/- 500KB	eQTL	Metabolite related risk factors in ERF
Glycine	rs715*	1.27×10^−32^	0.34	3'UTR	2	211251300	*CPS1, ACADL*	10.7	Chronic kidney disease[32], homocysteine levels[33], fibrinogen[3], glycine and metabolite levels[9], [34]–[36], lung cancer[37]	None	TG, CRP, creatine, eGFR, BMI
Proline	rs2540641	1.11×10^−19^	0.09	33 KB from *PRODH*	22	17339684	*PRODH, SLC25A1, PEX26*	2.9	Proline levels[9], citrate levels[7], metabolite levels[38], hyperprolinemia type 1, schizophrenia[15]	None	HDL-C, TG, insulin, BMI
Dimethyl-glycine^Novel^	rs248386	1.65×10^−19^	0.15	intronic	5	78365983	*DMGDH, BHMT, BHMT2, ARSB*	1.4	betaine[8], height[39], MD[40], ferritin levels[41], hippocampal atrophy[42], social sciences[43]	None	Albumin, resistin, creatin, creatinine, eGFR, urea, uric acid
Carnitine	rs1171614	4.81×10^−14^	0.18	5'UTR	10	61139544	*SLC16A9, FAM13C*	3.0	Urate level[10], [11]_ENREF_25, carnitine level[9], oleic acid level[44]	*SLC16A9*	TG, albumin, leptin, uric acid, BMI
Pyruvate^Novel^	rs1922005	1.26×10^−8^	0.13	intronic	2	217441741	*IGFBP5, IGFBP2, RPL37A*	1.8	Thyroid levels[45] teeth development[46], breast cancer[47]–[51], esophageal cancer[7], visceral fat[17], height[52]	None	TG, glucose, HOMA-IR, insulin, leptin, PWV, WHR, gynoid fat, BMI
3-Hydroxybutyrate^Novel^	rs9896573	1.65×10^−8^	0.09	6 KB from *KCNJ16*	17	65650639	None	0.1	Height[53], tooth development[54], thyrotoxic hypokalemic periodic paralysis[55], [56], eosinophilic esophagitis (pediatric)[57], palmitoleic acid[44], response to TNF-alpha inhibitors in rheumatoid arthritis[58], QT interval[59], cardiac repolarization[60], obesity-related traits (LDL)[61]	None	Adiponectin, lean mass index, android fat
Lysine	rs8056893	2.14×10^−8^	0.28	intronic	16	66861893	*SLC7A9, SMPD3, ZFP90, DPEP2, LCAT, EDC4, CDH1, ATP6V0D1, CTCF, PRMT7*	1.5	Glutaroyl carnitine/lysine[9], magnesium levels[62], HDL-C[63]-[67], ulcerative colitis[68], [69], HIV-1 viral setpoint[70], neurocognition (risperidone)[71], CD[72], [73], MetS[74], CHD[75]	*CDH1, SLC7A6, ATP6V0D1, CTCF, PRMT7, CPO48*	TC, HDL-C, glucose, albumin, DBP, CRP, transferrin saturation, fat %, fat mass index, lean mass idex, android fat, BMI
Valine^Novel^	rs11687765	3.49×10^−8^	0.44	intergenic	2	82179042	None	0.4	Bilirubin levels[1]	None	HDL-C, TG, glucose, adiponectine, HOMA-IR, insulin, SBP, PWV, urea, uric acid, ferritin, WHR, gynoid fat, BMI

*Also associated to creatine (P-value  =  1.40×10^−8^). MD; major depression, CD; Crohn's disease, MetS; metabolic syndrome, CHD; coronary heart disease. CRP; C-reactive protein, eGFR; glomerular filtration rate, PWV, pulse wave velocity. Phenotypes are shown that are associated with loci reported in GWAS catalog [22] and that lie within a 500 kb window of the main locus, regardless of linkage disequilibrium. Candidate genes 500 kb window around the best associated SNP were selected by automated workflow based on metabolic pathway information (see methods). eQTL lookups were perfomed in GTEX and GEUVADIS databases. Chr; chromosome; MAF; minor allele frequency; SNP; single nucleotide polymorphism; R^2^; Explained variance in metabolite level by the top SNP, eQTL; expression quantitative trait loci.

**Table 2:** Sequence variants within the coding regions of candidate genes that influence the metabolomic levels independent of the GWAS hits.

												Conditional analysis				
Metabolite	SNP	CHR	Position	A1	A2	Beta	SE	P-value^a^	Function	GENE	MAF	P-value^b^	P-value^c^	LD(r2)	Proxy SNPs	
Glycine	rs1047891	2	211540507	C	A	0.61	0.06	8.75×10^−26^	Missense	*CPS1*	0.24	8.07×10^−9^	4.05×10^−1^	0.92	*	
Glycine	rs182548513	2	211455113	G	C	−0.48	0.17	7.93×10^−3^	Intron	*CPS1*	0.02	2.55E×10^−5^	6.34×10^−22^	0.00	rs147937942, rs143738855	
3-Hydroxybuyrate	rs173135	17	68172326	C	T	0.40	0.08	1.50×10^−7^	3′ UTR	*KCNJ2*	0.11	2.46×10^−3^	3.11×10^−1^	0.72	*	
Proline	rs5747933	22	18910355	G	T	0.88	0.14	1.82×10^−9^	Missense	*PRODH*	0.03	7.30×10^−9^	2.89×10^−8^	0.04	rs2277834, rs4269009	
Proline	rs1076466	22	18907124	G	A	−0.17	0.05	6.34×10^−4^	Intron	*PRODH*	0.50	6.09×10^−6^	1.23×10^−11^	0.07	rs2008720, rs2008912	
Proline	rs13058335	22	18910479	C	T	0.66	0.09	2.46×10^−13^	Intron	*PRODH*	0.07	1.20×10^−5^	4.32×10^−1^	0.66	*	
Proline	rs3213491	22	19164835	A	C	0.38	0.11	7.48×10^−4^	Intron	*SLC25A1*	0.05	8.47×10^−5^	4.00×10^−10^	0.00		

A1; affect allele, A2; other allele, beta; effect estimate, SE; standard error of beta, P-value^ a^; p value of the association between the SNP and the metabolite, P-value^ b^; p-value of the association between the SNP and the metabolite, adjusted by the GWAS SNP, P-value^ c^; p-value of the association between the GWAS SNP and the metabolite, adjusted by the SNP.*Loci in which the GWAS is explained by the SNPs within the genes. Selection of significance for SNPs is based on P-value^ b^. Chr; chromosome; LD; linkage disequilibrium; MAF; minor allele frequency; SNP; single nucleotide polymorphism; eQTL; expression quantitative trait loci.

#### eQTL and functional effects

We used the GTEX and GEUVADIS [13] databases to check if the significantly associated SNPs affect cis gene expression. We obtained evidence that the leading GWAS SNP for carnitine (rs1171614) influenced the expression of *SLC16A9* in lymphoblasts (P-value  = 8.91×10^−6^) and rs8056893 (associated with lysine) influenced the expression of *ZPF90* in lymphoblasts (P-value  = 4.01×10^−6^) and *SLC7A9* in thyroid cells (P-value  = 0.00008). Rs248386 (associated with dimethyl-glycine) associated with the expression of *BHMT* (betaine—homocysteine S-methyltransferase) in the tibial nerve (P-value  = 0.000066). One of the missense variants; Thr1412Asn (rs1047891) in *CPS1* predicted to be “tolerated” by SIFT and “benign” by Polyphen functional predictions. The other missense variant Thr116Asn (rs5747933) on *PRODH* predicted to be “tolerated” by SIFT and “possibly damaging” by Polyphen.

#### Correlation with classical risk factors

Within the ERF population, we found that BMI correlated positively with carnitine (r = 0.136, P-value  = 4.40×10^−11^), proline (r = 0.123, P-value  = 2.80×10^−9^), pyruvate (r = 0.240, P-value  = 5.40×10^−32^), lysine (r = 0.132, P-value  = 1.45×10^−10^), and valine (r = 0.383, P-value  = 2.05×10^−82^) (S4-A Table), whereas BMI correlated negatively with glycine (r = −0.178, P-value  = 4.19×10^−18^). After additional adjustment for BMI, we observed that pyruvate, lysine and valine correlated positively with risk factors of T2DM, whereas glycine correlated negatively with triglycerides and C-reactive protein (CRP) (S4-B Table). Dimethyl-glycine particularly correlated with measures of kidney function; uric acid (r = 0.21, P-value  = 2.42×10^−9^), glomerular filtration rate (eGFR) (r = −0.14, P-value  = 2.53×10^−10^), urea (r = 0.18, P-value  = 1.20×10^−7^), and creatinine (r = 0.22, P-value  = 1.35×10^−22^).

We also explored possible relationships between the eight mQTL and the classical risk factors. Among the metabolites which associate with BMI, none of the mQTLs were associated with BMI itself in the ERF population. In addition, the association of the mQTLs with the metabolites glycine, carnitine, proline, pyruvate, lysine and valine did not change after adjustment for BMI (S5 Table). Interestingly, only for rs11687765 (valine-QTL) association with risk factors reached nominally significant P-values: specifically glucose (P-value  = 0.013), HOMA, insulin resistance (P-value  = 0.049) and gynoid fat mass (P-value  = 0.003). Association of rs11687765 with HOMA-insulin resistance dropped when adjusted by the valine level itself (P-value  = 0.122).

## Discussion

In this study, we report on the heritability, GWAS, candidate genes and fine genetic mapping of 42 metabolites identified and quantified using ^1^H-NMR spectroscopy in the Erasmus Rucphen Family (ERF) study. In 2009, the first GWAS of metabolites identified by ^1^H-NMR spectroscopy measured in human plasma was reported by Chasman *et al.* [4]. This study focused primarily on lipoprotein particle size and content, and did not measure other metabolites such as organic acids and amino acids, yet reported 43 significant metabolite mQTL. This was followed by three reports on blood and urine samples [5], [6] the largest of which by Kettunnen *et al.* involving both small metabolites and lipoprotein particle sizes, reporting 31 novel mQTL [7]. Recently, Rueedi *et al.* reported one novel locus using an untargeted approach [12]. Here, we used ^1^H-NMR J-Resolved 2D spectrometry followed by spiking experiments, yielding a reliable certain metabolite identification. Traditional CVD traits in ERF and other cohorts in general show a heritability ranging from 20% to 30% [14]. In the present study, we observed a similar distribution of heritability for NMR detected metabolites, ranging from 10% to 52%. These heritability estimates seem somewhat lower than those found in the NMR GWAS by Kettunen *et al.* [7]. However, in that report a significant proportion of the reported NMR traits and heritability estimates concern lipoprotein particle characteristics. Since, in general, heritability for lipoproteins is high [7], ranging from 30% to 50%, this could explain the apparent discrepancy with our reported heritability data.

Using verified metabolites, we replicated three known loci and uncovered a novel association for dimethyl-glycine in the vicinity of the biologically plausible genes *DMGDH* and *BHMT*. This was expected since our study had 62 to 100% power to detect genetic variants with 0.2 to 0.5 effect size with metabolome-wide significant P-value (1.1×10^−9^) for a bi-allelic marker with 0.3 MAF (for instance rs715 in *CPS1*) based on the assumption of complete LD with the causal genetic variant. For more rare variants with larger effect size such as rs248386 in *DMDGH* with 0.15 MAF and 0.4 effect size the power on metabolome wide significance was 100%. Furthermore, we report suggestive common genetic variants; first in an intergenic region on chromosome 2 for valine, second in *TNP1* for pyruvate and lastly in *KCNJ16* for 3-hydroxybutyrate levels. Analysis of the coding sequence in the candidate genes uncovered potentially causal signals within *CPS1, KCNJ2* and *PRODH* that explain the GWAS hits, as well as additional independent signals located in *CPS1* and *PRODH* indicating allelic heterogeneity within these genes. Among the eight mQTL, rs715 in CPS1 explained the highest (10%)of the total phenotypic variance in circulating glycine levels (Table 1).This was higher than the total explained variance in for glycine level by age and sex. (S6 Table).

The *CPS1* locus has been previously found associated with kidney disease, homocysteine, and several metabolite levels including glycine. *CPS1* mutations are known to cause carbamoylphosphate synthetase I deficiency, an autosomal recessive inborn error of metabolism of the urea cycle which causes hyperammonemia. The disease may also have a delayed onset in adulthood and is associated with chronic kidney disease. Gene-network predictions for this gene included functions such as triglycerides (TG) and lipoprotein homeostasis. In our study, we also found association of the same SNP with creatine level and also observed a significant correlation between creatine and glycine (r = 0.08, P-value  = 1.46×10^−4^), glomerular filtration rate (r = −0.09, P-value  = 7.07×10^−5^) and TG (r = −0.08, P-value  = 1.15×10^−4^). We identified Thr1412Asn in *CPS1* as a potential variant that may alter the protein function. The second independent signal within *CPS1* was located intronic (rs182548513). The neighbouring SNP, rs147937942, (Table 2) in LD with rs182548513 is located on 5′UTR of a *CPS1* transcript variant (*CPS1-001*), and identified as transcription factor binding site according to the ENCODE database however, so far we did not find any evidence that the SNP affects expression which may be tissue specific.

The second locus, *PRODH*, a gene highly expressed in cerebral cortex, cerebrum and other brain tissues is known to be involved in proline metabolism, but also in central nervous system myelination. The locus was previously shown to associate with schizophrenia [15] and autism [16]. We show in total 4 independent SNPs that associate with circulating proline level; including (1) the GWAS hit, (2) one very common SNP (tagged by rs2008720), (3) a possibly damaging missense mutation with low frequency (MAF  = 0.03, Thr167Asn) and (4) another with MAF  = 0.05 (rs3213491). It is important to mention that rs2008720 maps to first exon of *PRODH (PRODH-001 isoform)* resulting the amino-acid change Pro19Gln, whereas it also maps to the promoter regulatory region of another *PRODH* isoform (*PRODH-004*). Neither for these variants did we find experimental evidence from eQTL database.

*DMGDH* codes for the enzyme dimethyl-glycine dehydrogenase which is involved in catabolism of choline, catalyzing the oxidative demethylation of dimethyl-glycine to form sarcosine. The gene is highly expressed in liver, followed by kidney. Mutations in this gene cause an inborn error of metabolism characterized by unusual fish-like body odour. Functional predictions for this gene by KEGG database include several functions in amino-acid metabolism and bile acid synthesis. Conditional analysis in this region showed that the GWAS hit located intronic in *DMGDH* (rs248386) is most likely the causal variant. Interestingly we found this SNP associated with the expression of the neighbouring gene, *BHMT* that is also involved in dimethyl-glycine and betaine metabolism.

*SLC16A9* is involved in drug transport, bile salt and organic anion transport and has been previously shown to be associated with carnitine, uric acid levels. In the ERF population carnitine and uric acid are highly correlated (r = 0.25, P -value  = 3.93×10^−13^). For this locus, we did not find any potentially causal coding variants. However, the GWAS hit (rs1171614) located 5′UTR of *SLC16A9* influences the expression of *SLC16A9* in both GTEX and GEUVADIS databases, indicating that the effect on carnitine level is possibly through expression, rather than the change in protein function.

The metabolite pyruvate is the product of anaerobic glycolysis. Pyruvate levels correlate with gynoid adipose tissue mass, BMI, waist hip ratio, TG, glucose, HOMA-IR and leptin in the ERF population (S4B Table). Genes in the *TNP1* locus, particularly *IGFBP5* have been previously associated with visceral adipose tissue mass in men [17]. Within these genes, we did not find any causal variants, neither for the GWAS hits were we unable to uncover downstream eQTL. For 3-hydroxybutyrate, rs173135 located in the 3′UTR of *KCNJ2* is the most likely causal variant tagged by the GWAS hit for 3-hydroxybutyrate. The gene is predominantly expressed in heart muscles but also in brain and the locus has been previously associated with QT interval and cardiac repolarization. Currently, it is not known how this gene may be affecting 3-hydroxybutyrate levels. The association between *SLC7A9* and valine has previously been shown [9]. Within the candidate genes in this locus, we were not able to detect any causal variants. However, the leading GWAS SNP is associated with expression of *SLC7A9* and *ZPF90.* Finally, valine has been suggestively associated with an intergenic region with no eQTL association. This region has been previously shown to associate with bilirubin level, which is a determinant of hepatic health. The strong correlation between valine and pyruvate levels and the risk factors of T2DM suggests these loci are candidates for T2DM research. Using the data from the ERF population, for 7 out of 8 loci, we found no evidence that the mQTL discovered directly or indirectly influenced the risk factors for common diseases. Our data indicate that the association between these mQTLs and the metabolites were independent of disease risk factors. For BMI, our results support an additive effect of BMI and mQTL, both influencing the metabolite levels. We did find evidence for an association between HOMA insulin resistance, valine and rs11687765. However, this finding asks for replication in independent larger sized studies.

Altogether, our study provides strong evidence for associations of metabolic traits with a range of novel and previously detected genetic loci. These loci are potentially of biomedical and pharmaceutical interest, and may provide insight into human metabolic and disease pathways.

## Methods

### Study cohort

The Erasmus Rucphen Family (ERF) study is a cross-sectional cohort including 3000 living descendants of 22 couples who had at least 6 children baptized in the community church around 1850-1900. The participants are not selected on any disease or other outcome (S1 Table). Details about the genealogy of the population have been described elsewhere[18]. The study protocol was approved by the medical ethics board of the Erasmus MC Rotterdam, the Netherlands.

### ^1^H-NMR JRES measurements

2,640 sera of ERF participants were submitted for ^1^H-NMR experiments. All NMR experiments were acquired on a 600 MHz Bruker Avance II spectrometer (Bruker BioSpin, Karlsruhe, Germany). For this study the 2D J-resolved (JRES) and CPMG (Carr-Purcell-Meiboom-Gill) methods were used. Data processing was performed in Topspin and Matlab (R2009a, The Mathworks Inc., Natick, MA, USA). After eliminating low-quality spectra after a QC procedure, metabolite intensities were obtained from the serum CPMG spectra by applying a linear model. The model was constructed by identifying well-resolved peaks in the 2D JRES spectrum, and relating the intensity of the peak representing the metabolite with the intensity profile of the much more convoluted CPMG spectrum. This way, the higher resolution of the JRES 2D spectrum is combined with the better signal-to-noise of the CPMG spectrum. After quality control peaks in the JRES projection were automatically deconvoluted by fitting the spectra with mixed Gauss-Lorentz line-shapes using the Simplex method yielding 256 deconvoluted peaks, 42 metabolites could be reliably assigned using a combination of chemical shift interpretation, cross-correlation between peaks and spiking of pure compounds in a mixed serum sample of them were annotated to unique metabolites (S2 Table). Further selection procedure and QC and the list of unique metabolites studied are given in the supplement.

### Heritability analysis

Heritability estimations for all metabolite concentrations were obtained using SOLAR version 6.6.2 software using a polygenic model and sex and age as covariates.

### Genome-wide association analyses

Data points below or above 4 standard deviations from the mean were removed and non-missing data points of all variables were rank transformed using the “rank” function in R, this function takes the missing values into account. No samples were detected as outliers. DNA samples were genotyped according to the manufacturer's instructions on Illumina Infinium HumanHap300v2, HumanHap300v1 or HumanCNV370v1 SNP bead microarrays. Genotype data were imputed using MACH 1.0 (v1.0.18.c) using the HapMap CEU population (release 22, build 36). As the ERF study included related individuals, testing for association between lipid and allele dosage was performed using a mixed model approach as implemented with the ‘mmscore’ option in the GenABEL software. 1.7–4 (R 2.15.3) [19]. This option combines the Family Based Score Test for Association (FASTA) method of Abecasis et al. [20] and kinship matrix estimated from genotyped SNPs [21]. The total genotype set after imputation involved dosage information of approximately 2.5 million SNPs. Among the 2,640 samples, 2,416 were genotyped, following the exclusion of people on lipid lowering (N = 298), in total 2,118 samples were included in the final analysis. To correct for multiple testing, we used the number of unique metabolites (N = 42) which yielded a suggestive significance zone that lies between 5×10^−8^ and 1.2×10^−9^. Details are described in the S2 Text.

### Automated annotation of GWAS results

In order to facilitate the manual process of assigning genes to a locus, we used an automated workflow developed in-house to generate reports containing the associated protein, enzyme, metabolic reaction, pathway, and disease phenotypes of every gene within a window of 1 MB of the locus. In addition, SNPs published in the GWAS catalog [22] and eQTLs from the GTEx-eQTL database. (http://www.ncbi.nlm.nih.gov/gtex/GTEX2) were given. In detail, the reports created by our workflow were based on the dbSNP [23], NCBI-Gene (http://www.ncbi.nlm.nih.gov/gene), GTEx-eQTL, GWAS catalog, ConsensusPathDB [24], UniProtKB [25], OMIM [26], TCDB [27], ExPASy [28] and KEGG database [29]. The databases had been downloaded earlier from the respective ftp servers and have been integrated offline in Matlab. For the KEGG database the last freely available version was used (30-6-2011).

### Exome sequencing

Coding variant analysis were performed within the 3^rd^ data freeze (N = 1309) from the ERF pedigree which were sequenced “in-house” at the Center for Biomics of the Cell Biology department of the Erasmus MC, The Netherlands, using the Agilent version V4 capture kit on an Illumina Hiseq2000 sequencer using the TruSeq Version 3 protocol. The sequence reads were aligned to the human genome build 19 (hg19) using BWA and the NARWHAL pipelines [30], [31]. After processing, genetic variants were called using the Unified Genotyper tool from the GATK. The effects of the called variants on the respective protein sequences were determined with a custom variant annotation script. For each sample, at least 4 Gigabases of sequence was aligned to the genome. All variants in the vicinity of the genes of interest were selected for further analysis. Variants with less than 5 observations were removed. Of the 1,309 individuals with exome sequencing data, 921 had eligible NMR metabolite measurements. Single variant analyses were performed using and additive model as implemented in the “mmscore” function in GenABEL v.1.7–4, adjusting for relatedness.

### Ethics statement

The study protocol was approved by the medical ethics board of the Erasmus MC Rotterdam, the Netherlands. The study included only adults and written informed consents were provided by all the subjects participated in the study.

## Supporting Information

S1 Fig Q-Q plots of the top regions.
(PDF)

S2 Fig Regional association plots of the top regions.
(PDF)

S1 Text Results from automated selection.
(PDF)

S2 Text Methods on NMR spectroscopy, genotyping, exome sequencing and statistics.
(PDF)

S1 Table Characteristics of ERF study sample.
(PDF)

S2 Table Unique NMR Metabolite peaks selected for GWAS. In total we studied 42 uniquely annotated NMR peaks.
(PDF)

S3 Table Results from exome sequence association study.
(XLS)

S4 Table Correlation to risk factors of disease.
(PDF)

S5 Table Association between metabolites and mQTL adjusted by BMI.
(PDF)

S6 Table The effect of age and gender on metabolite levels.
(PDF)
