# Supplementary material for: Insight in Genome-Wide Association of Metabolite Quantitative Traits by Exome Sequence Analyses
Source: PLoS Genet. 2015 Jan 8;11(1):e1004835. doi: 10.1371/journal.pgen.1004835 (PMC4287344; doi:10.1371/journal.pgen.1004835)

Supplementary Figure 1. Q-Q plots (1)

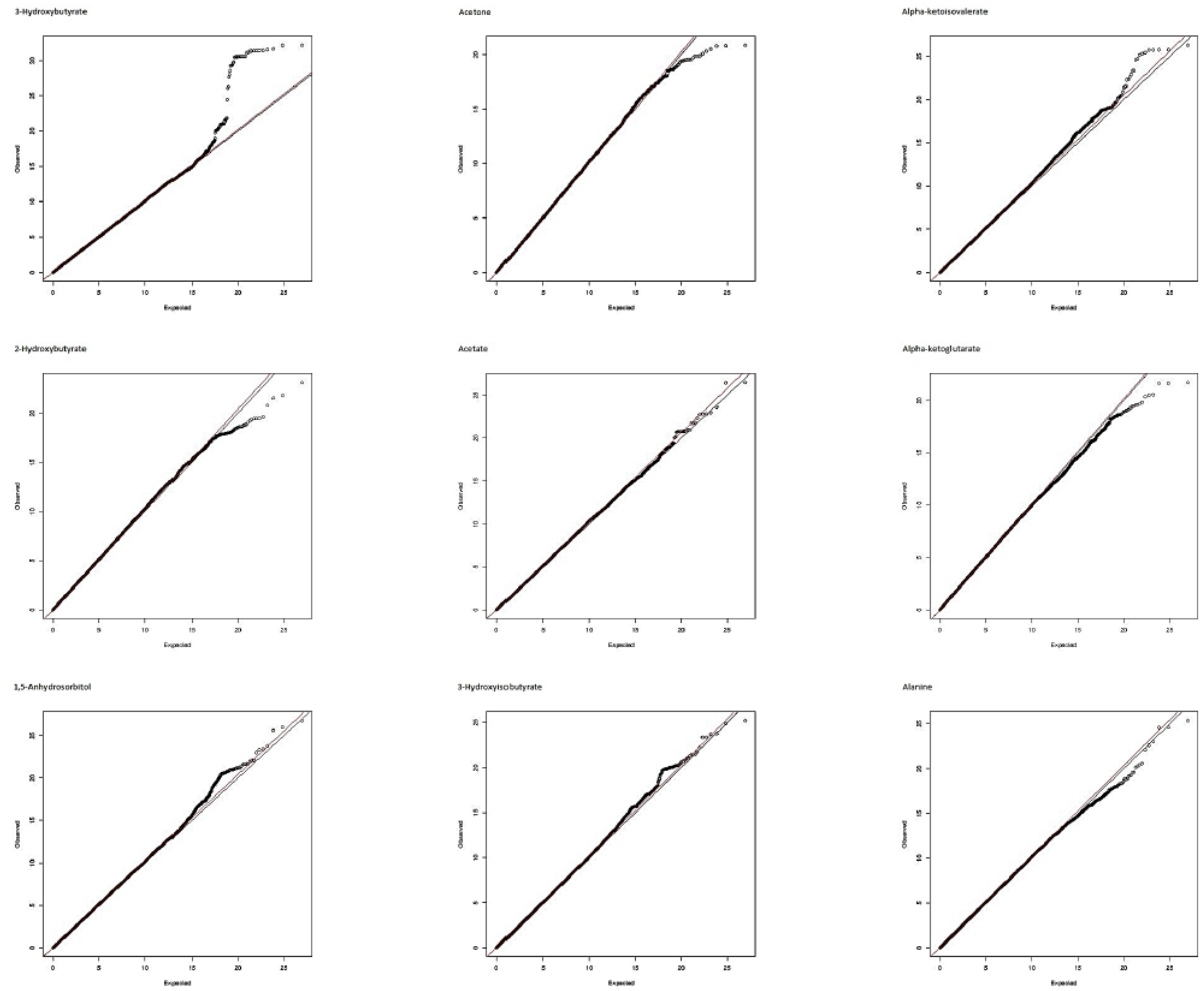

Supplementary Figure 1. Q-Q plots (2).

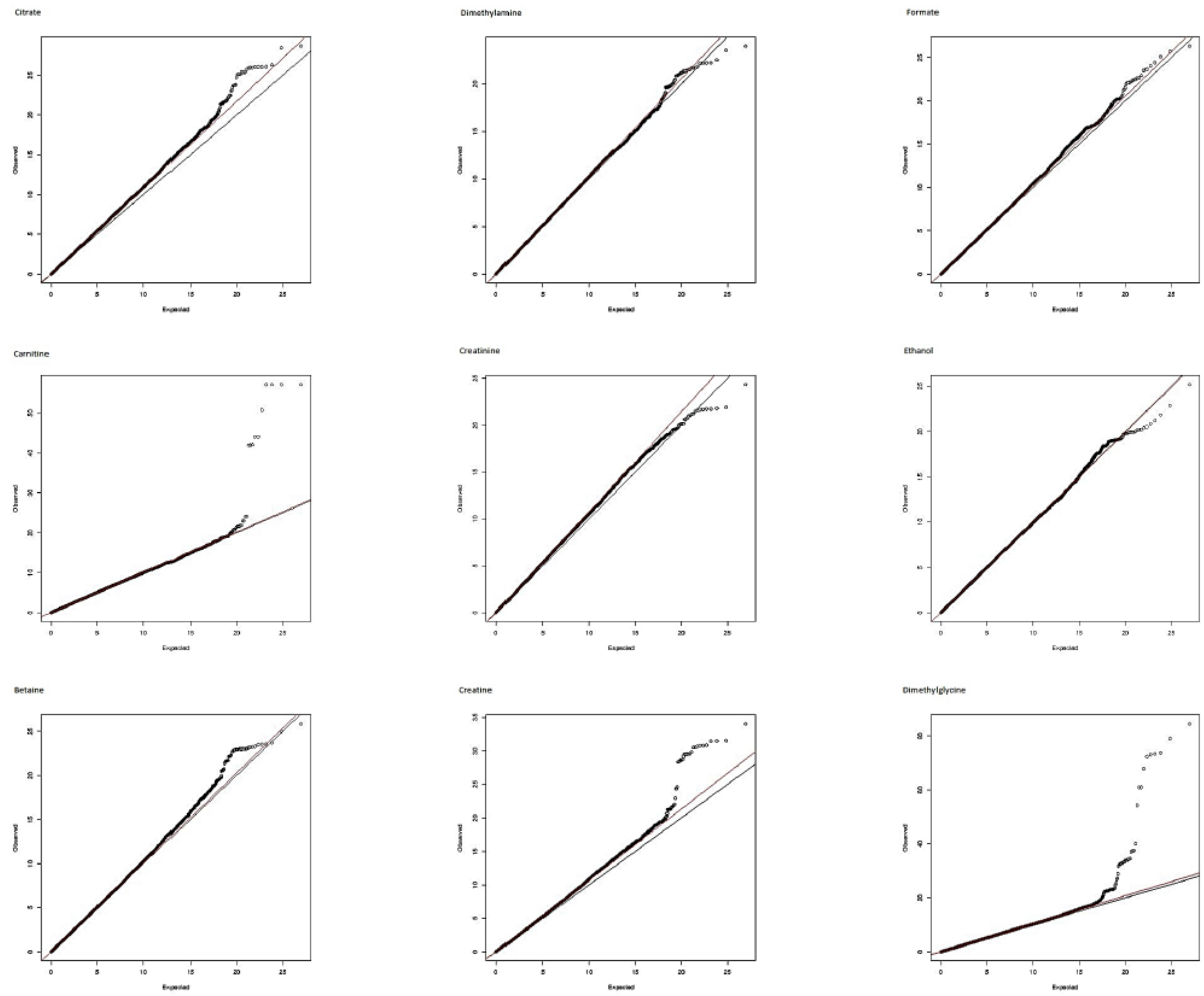

Supplementary Figure 1. Q-Q plots (3).

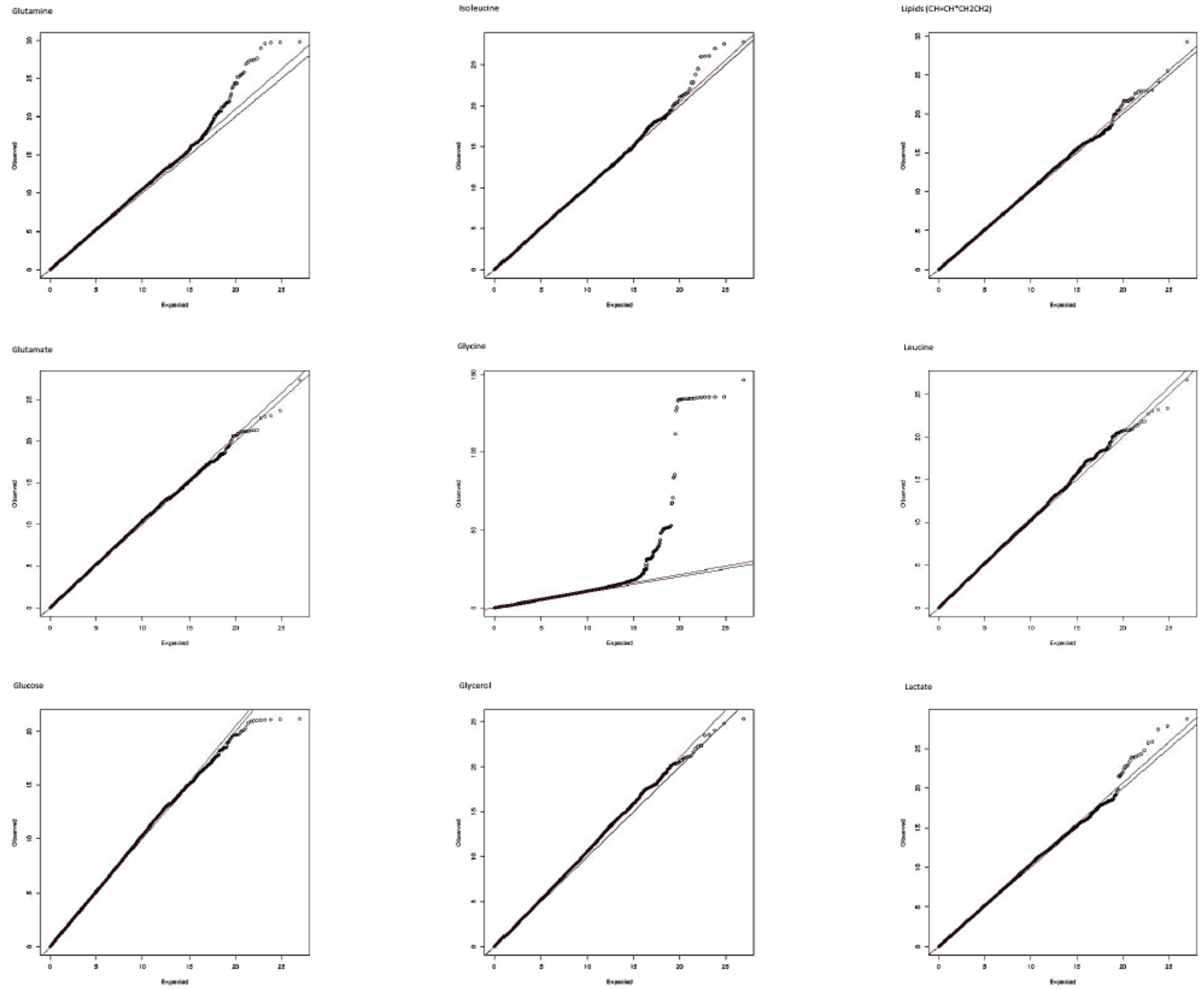

Supplementary Figure 1. Q-Q plots (4).

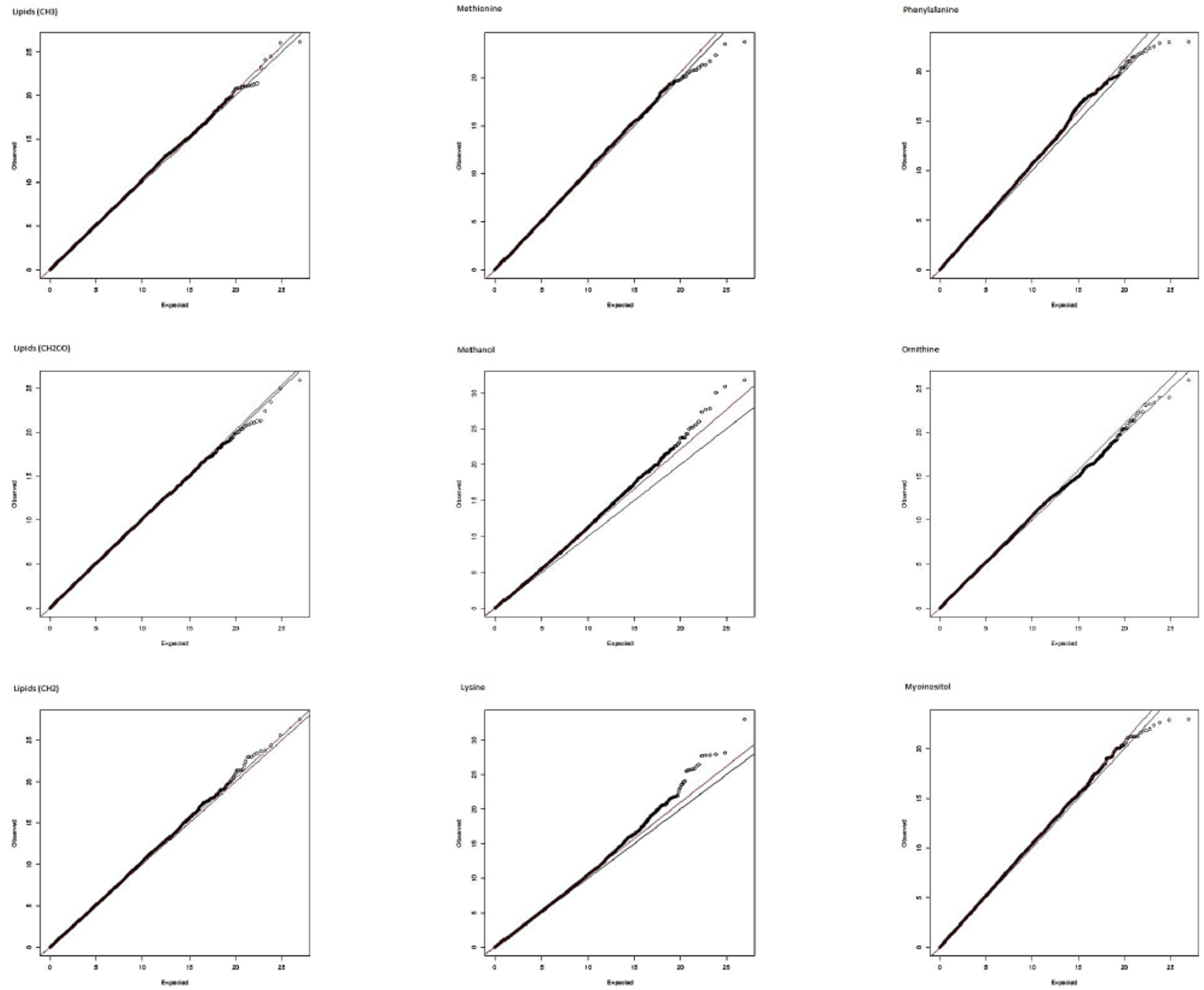

Supplementary Figure 1. Q-Q plots (5).

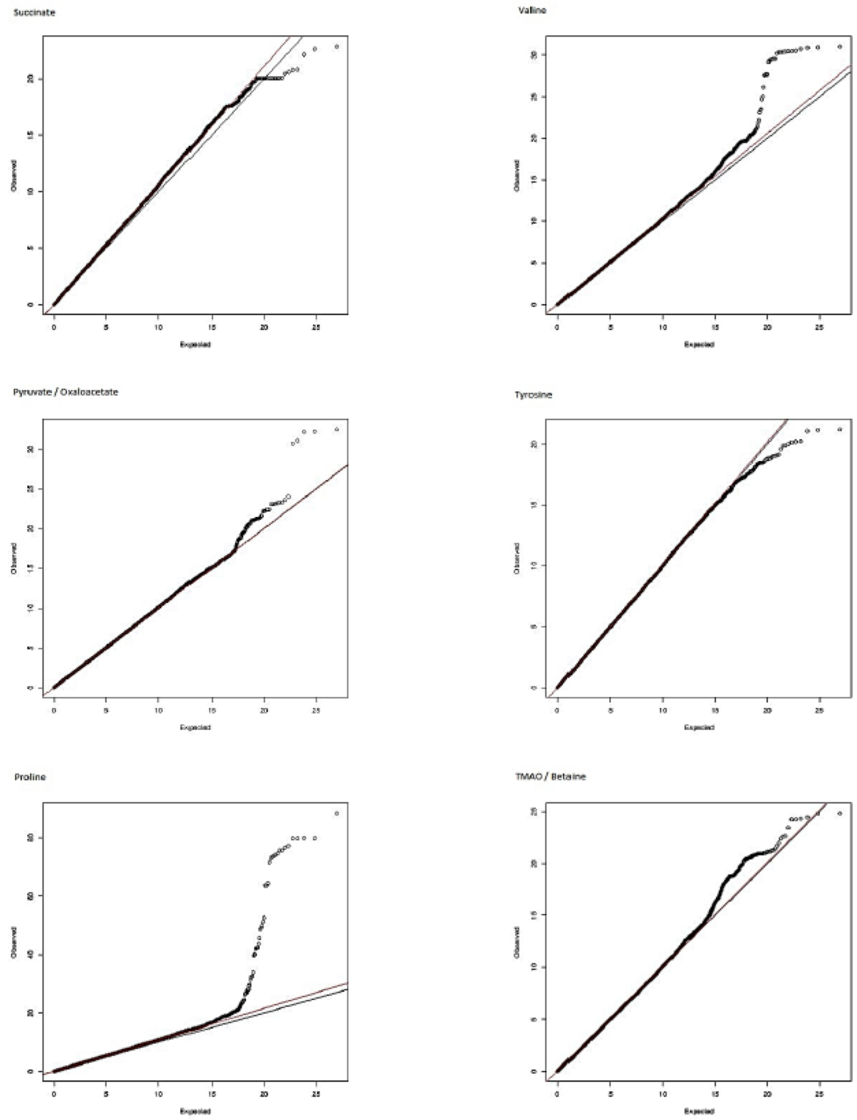

Supplement: S1 Fig — Q-Q plots of the top regions. (PDF) [file pgen.1004835.s001.pdf]
