# Supplementary figures and images for: Insight in Genome-Wide Association of Metabolite Quantitative Traits by Exome Sequence Analyses
Source: PLoS Genet. 2015 Jan 8;11(1):e1004835. doi: 10.1371/journal.pgen.1004835 (PMC4287344; doi:10.1371/journal.pgen.1004835)

**Supplementary Figure 2. Regional plots of the GWAS loci**

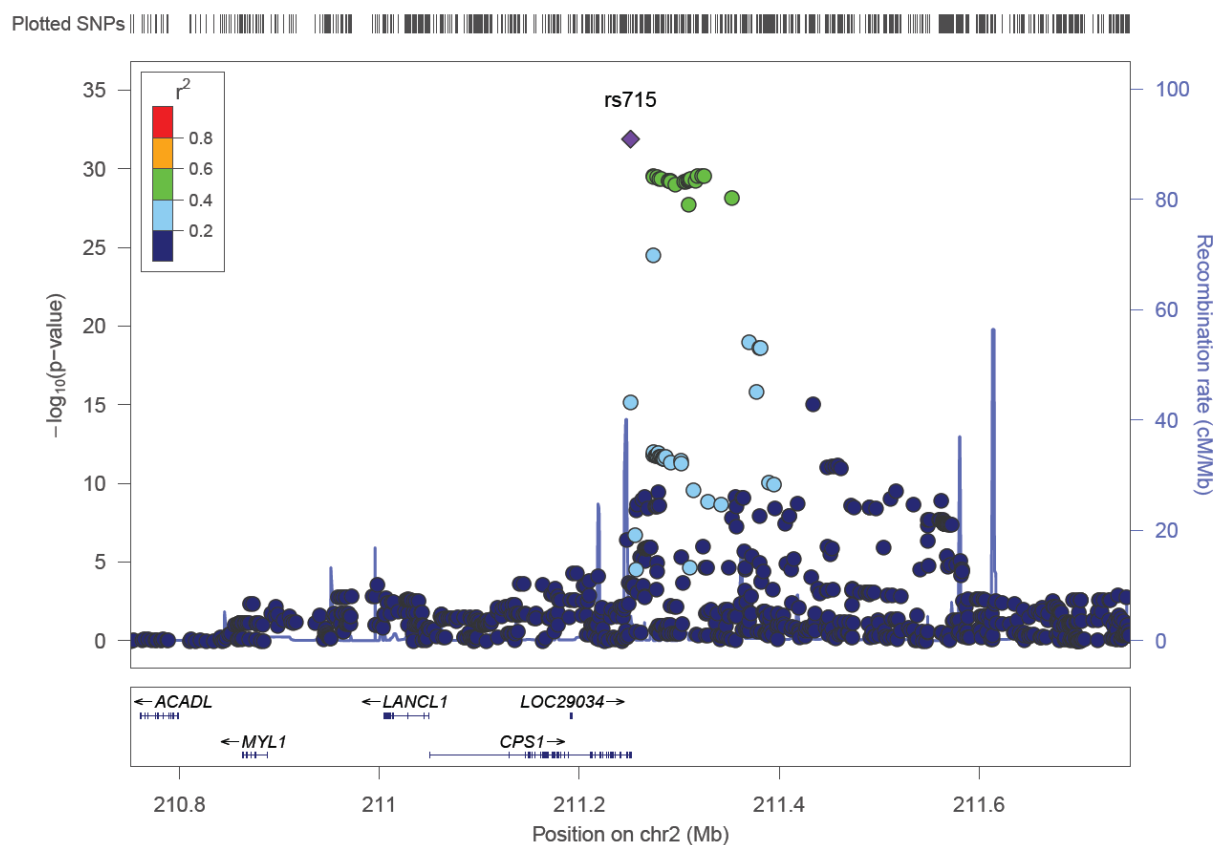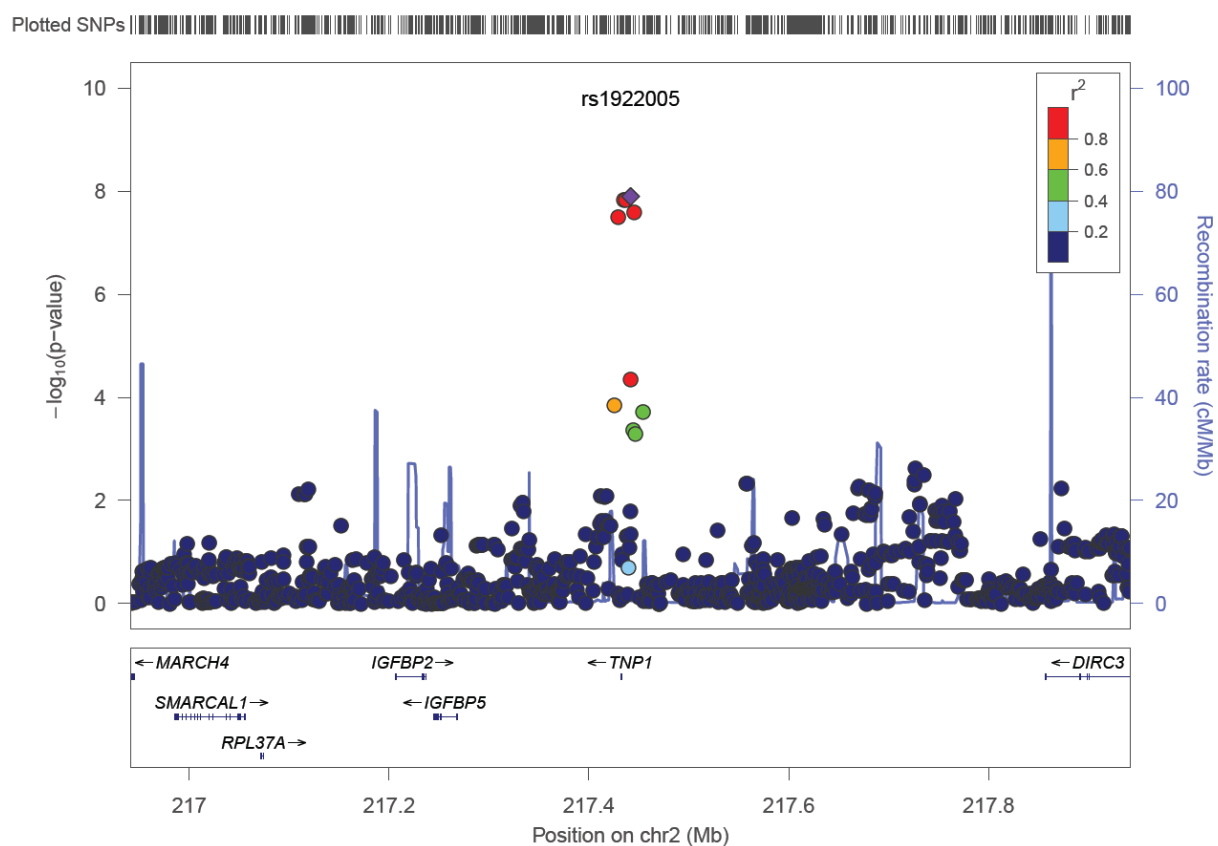

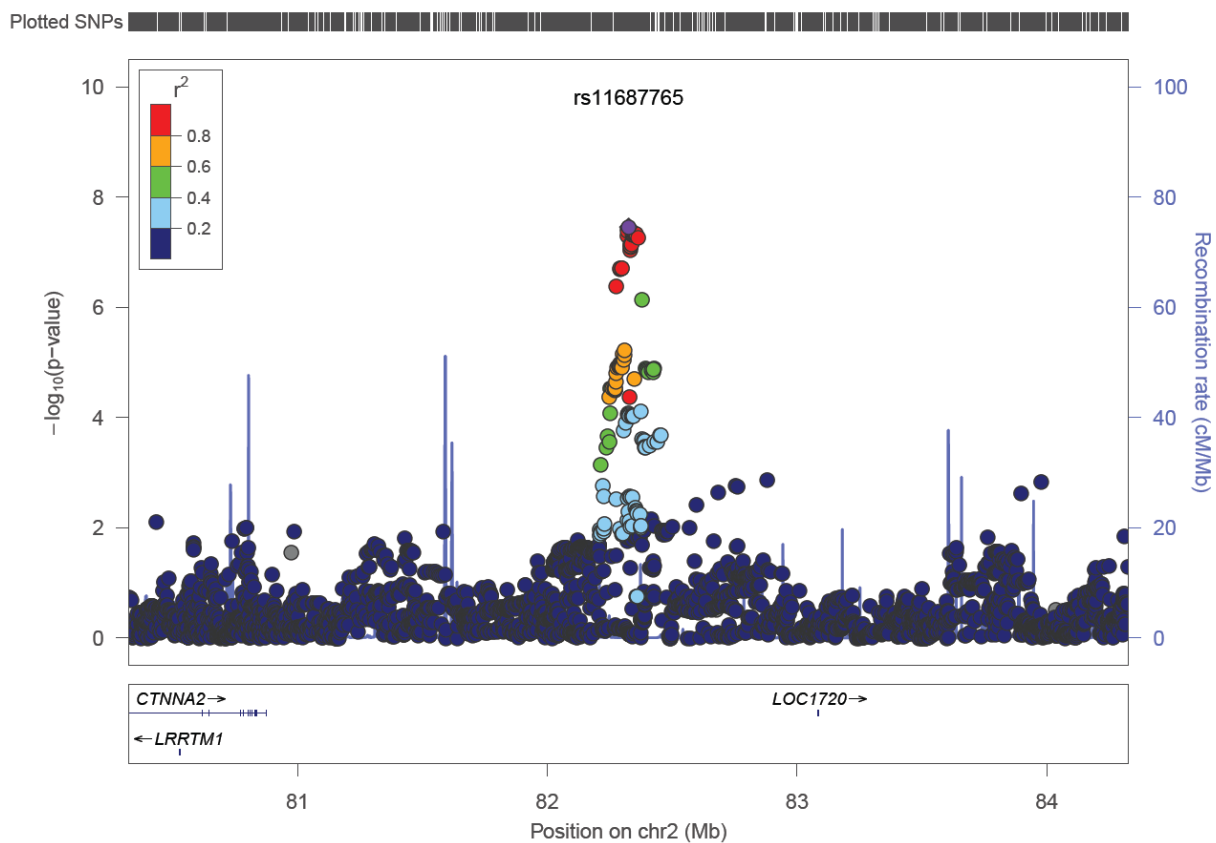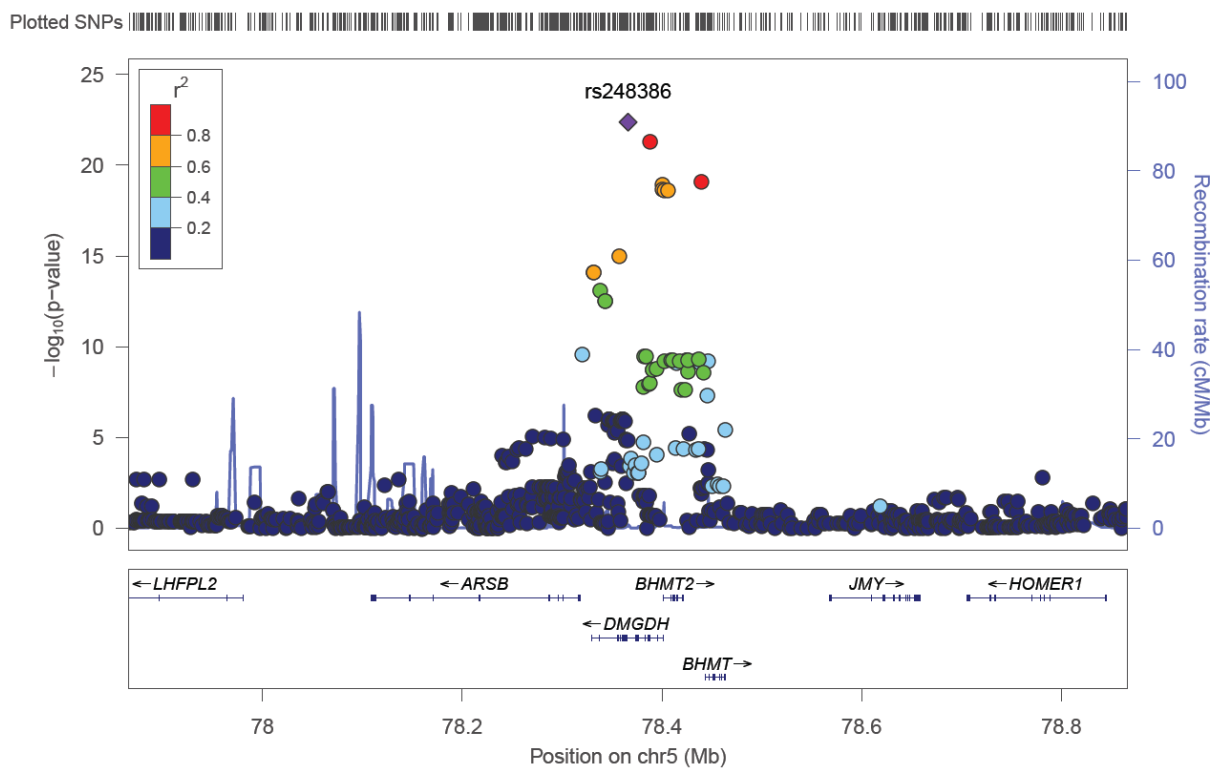

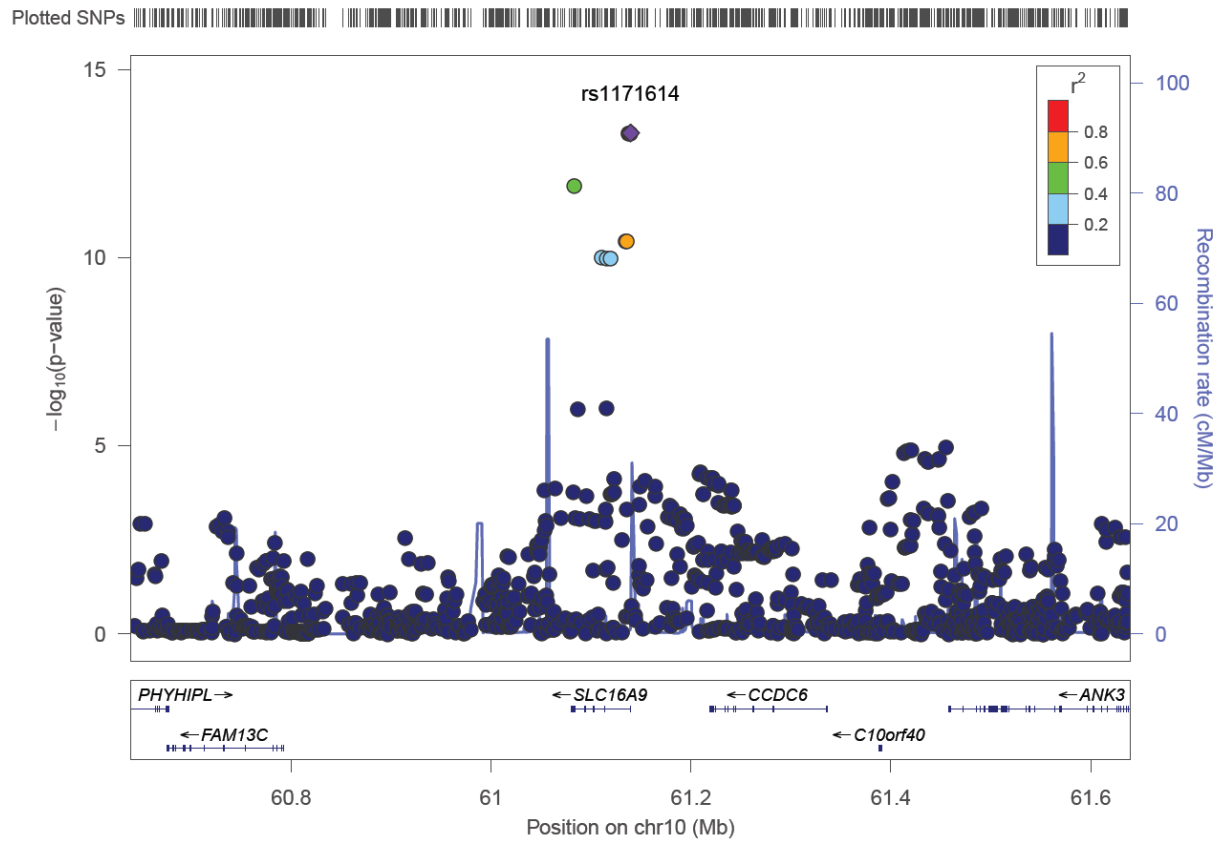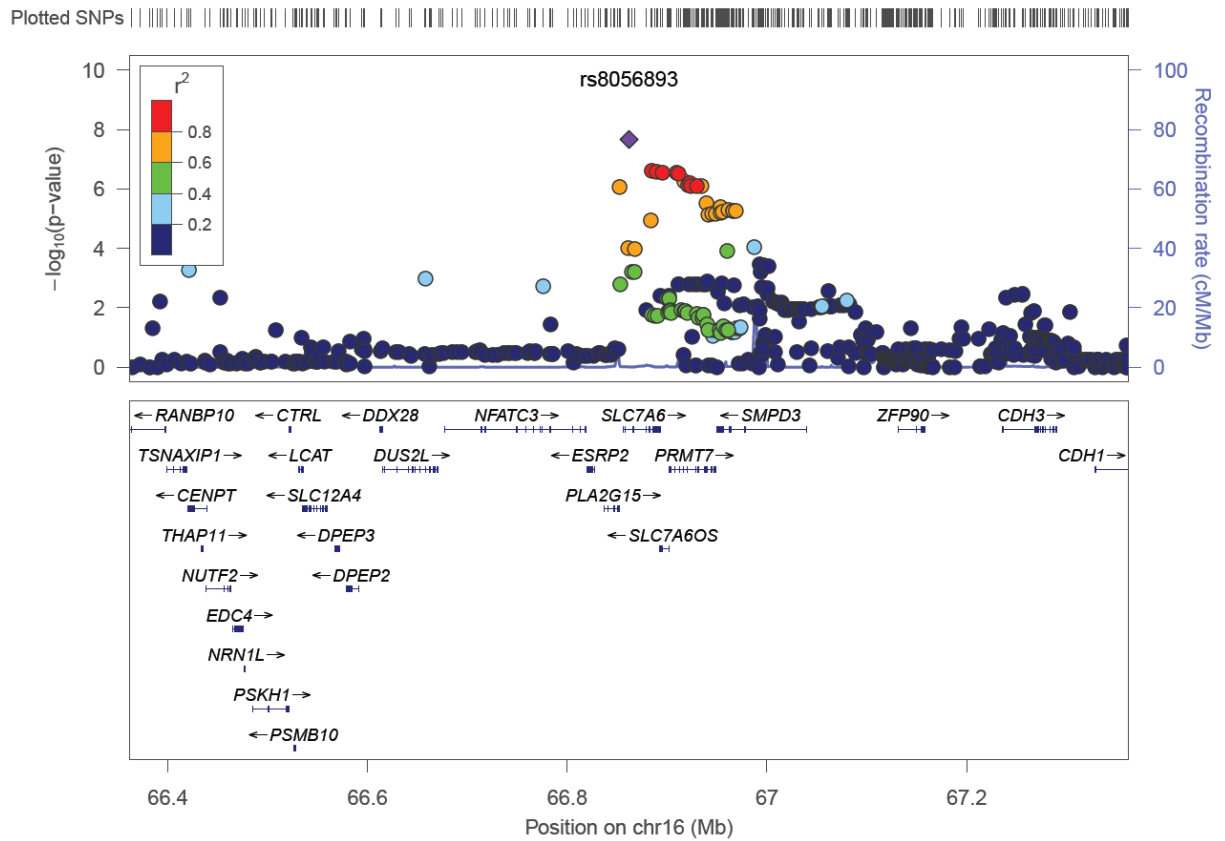

Plotted SNPs

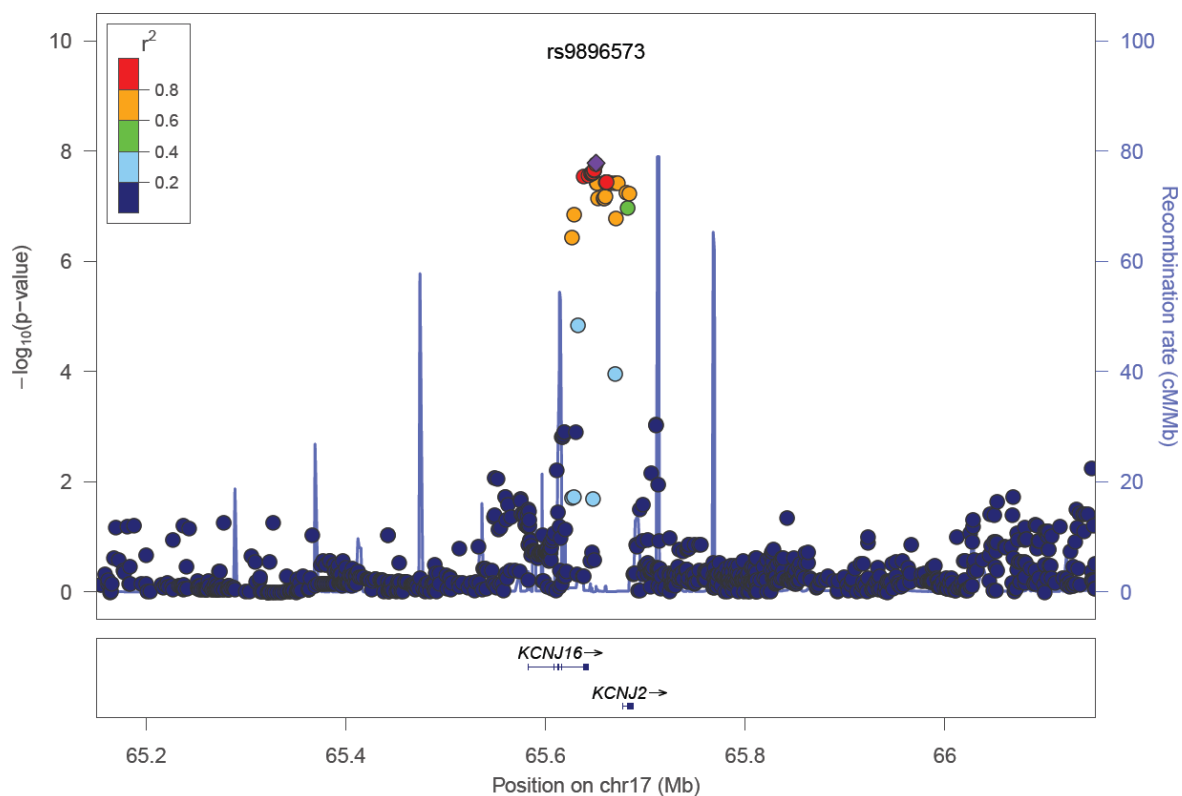

Plotted SNPs

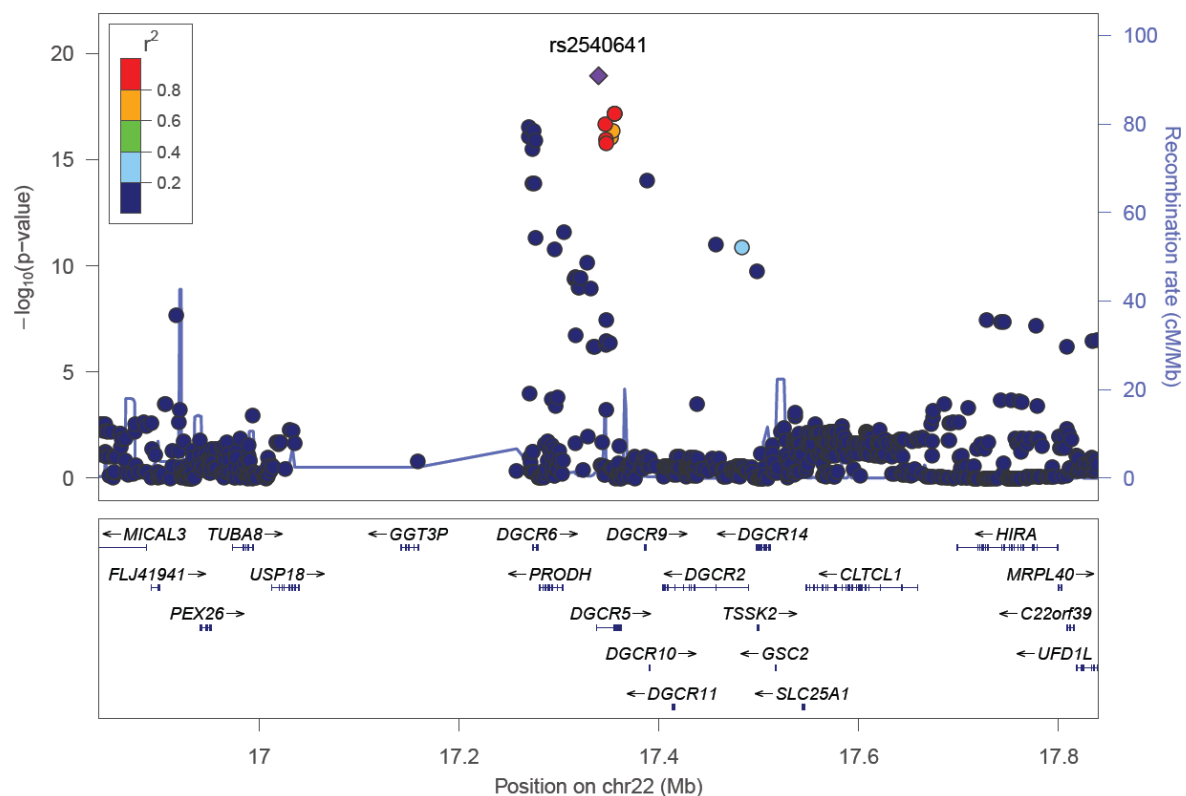

Supplement: S2 Fig — Regional association plots of the top regions. (PDF) [file pgen.1004835.s002.pdf]
