# Supplementary material for: Insight in Genome-Wide Association of Metabolite Quantitative Traits by Exome Sequence Analyses
Source: PLoS Genet. 2015 Jan 8;11(1):e1004835. doi: 10.1371/journal.pgen.1004835 (PMC4287344; doi:10.1371/journal.pgen.1004835)
Supplement: S1 Text — Results from automated selection. (PDF) [file pgen.1004835.s003.pdf]


Mapping SNP rs1171614 Chr 10 61469538

Distance to locus: 0
    Gene: 220963 (SLC16A9) Chr 10 61410522-61469649 (-)
        Pathway: Glycosphingolipid metabolism (database: EHMN)
        Pathway: Glycerophospholipid metabolism (database: EHMN)
        Protein: MOT9 Monocarboxylate transporter 9;
            KO: K08186 MFS transporter, MCP family, solute carrier family 16 (monocarboxylic acid transporters), member 9
        OMIM: *614242 SOLUTE CARRIER FAMILY 16 (MONOCARBOXYLIC ACID TRANSPORTER), MEMBER9; SLC16A9

Distance to locus: -27210
    Gene: 100507027 (M1) Chr 10 61496748-61513203 (-)

Distance to locus: -78967
    Gene: 8030 (CCDC6) Chr 10 61548505-61666414 (-)
        Pathway: Pathways in cancer - Homo sapiens (human) (database: KEGG)
        Pathway: Thyroid cancer - Homo sapiens (human) (database: KEGG)
        Protein: CCDC6 Coiled-coil domain-containing protein 6;
            KO: K09288 coiled-coil domain-containing protein 6
        OMIM: *601985 COILED-COIL DOMAIN-CONTAINING PROTEIN 6; CCDC6

Distance to locus: 157902
    Gene: 359751 (MRPL50P4) Chr 10 61311157-61311636 (-)

Distance to locus: -248437
    Gene: 283025 (C10orf40) Chr 10 61717975-61720671 (-)

Distance to locus: -316518
    Gene: 288 (ANK3) Chr 10 61786056-62493284 (-)
        Pathway: Developmental Biology (database: Reactome)
        Pathway: Interaction between L1 and Ankyrins (database: Reactome)
        Pathway: L1CAM interactions (database: Reactome)
        Pathway: Axon guidance (database: Reactome)
        Protein: ANK3 Ankyrin-3;
            KO: K10380 ankyrin
        OMIM: *600465 ANKYRIN 3; ANK3

Distance to locus: 346877
    Gene: 220965 (FAM13C) Chr 10 61005889-61122661 (-)
        Protein: FA13C Protein FAM13C;

Distance to locus: 462004
    Gene: 84457 (PHYHIPL) Chr 10 60936348-61007534 (+)
        Protein: PHIPL Phytanoyl-CoA hydroxylase-interacting protein-like;

GWAS catalog
----

rs1171614 Chr 10 61469538 (0) SLC16A9 Urate levels [Kottgen A, 12/23/2012, Nat Genet]
rs7094971 Chr 10 61449564 (19974) SLC16A9 Metabolic traits (carnitine) [Suhre K, 08/31/2011, Nature]
rs12356193 Chr 10 61413353 (56185) SLC16A9 Uric acid levels [Kolz M, 06/05/2009, PLoS Genet]
rs11006464 Chr 10 61146582 (322956) FAM13C1 Oleic acid (18:1n-9) plasma levels [Wu JH, 01/29/2013, Circ Cardiovasc Genet]

----
eQTL mapping (NCBI-GTEx database)

LD r^2	rs1171614	Gene	distance
rs1171638	0.0025649	SLC16A9	14769
rs10128501	0.044716	SLC16A9	-39246
rs6479648	0.033617	SLC16A9	-41021
rs1913507	0.014285	SLC16A9	-49451
rs1913506	0.014285	SLC16A9	-49557

Mapping eQTL-SNP rs1171638 Chr 10 61454769 p-value = 5.6951e-010 tissue = Lymphoblastoid
Mapping eQTL-SNP rs10128501 Chr 10 61508784 p-value = 2.01e-008 tissue = Brain Cerebellum
Mapping eQTL-SNP rs6479648 Chr 10 61510559 p-value = 2.01e-008 tissue = Brain Cerebellum
Mapping eQTL-SNP rs1913507 Chr 10 61518989 p-value = 3.24e-008 tissue = Brain Cerebellum
Mapping eQTL-SNP rs1913506 Chr 10 61519095 p-value = 3.24e-008 tissue = Brain Cerebellum
Gene: 220963 (SLC16A9) Chr 10 61410522-61469649 (-)
Pathway: Glycosphingolipid metabolism (database: EHMN)
Pathway: Glycerophospholipid metabolism (database: EHMN)
Protein: MOT9 Monocarboxylate transporter 9;
KO: K08186 MFS transporter, MCP family, solute carrier family 16 (monocarboxylic acid transporters), member 9
OMIM: *614242 SOLUTE CARRIER FAMILY 16 (MONOCARBOXYLIC ACID TRANSPORTER), MEMBER9; SLC16A9

Mapping SNP rs8056893 Chr 16 68304392

Distance to locus: 0
    Gene: 9057 (SLC7A6) Chr 16 68298423-68335722 (+)
        Pathway: Lysinuric Protein Intolerance (database: SMPDB)
        Pathway: Hartnup Disorder (database: SMPDB)
        Pathway: Leukocyte TarBase (database: Wikipathways)
        Pathway: Lymphocyte TarBase (database: Wikipathways)
        Pathway: Muscle cell TarBase (database: Wikipathways)
        Pathway: SLC-mediated transmembrane transport (database: Reactome)
        Pathway: Amino acid transport across the plasma membrane (database: Reactome)
        Pathway: Amino acid and oligopeptide SLC transporters (database: Reactome)
        Pathway: Transport of inorganic cations/anions and amino acids/oligopeptides (database: Reactome)
        Pathway: Transmembrane transport of small molecules (database: Reactome)
        Pathway: Basigin interactions (database: Reactome)
        Pathway: Glycine, serine, alanine and threonine metabolism (database: EHMN)
        Pathway: Amino acid transport across the plasma membrane (database: PID)
        Pathway: Cell surface interactions at the vascular wall (database: Reactome)
        Pathway: Hemostasis (database: Reactome)
        Protein: YLAT2 Y+L amino acid transporter 2;
            KO: K13872 solute carrier family 7 (L-type amino acid transporter), member 6
        OMIM: *605641 SOLUTE CARRIER FAMILY 7 (CATIONIC AMINO ACID TRANSPORTER, y+ SYSTEM),MEMBER 6; SLC7A6

Distance to locus: 9431
    Gene: 23659 (PLA2G15) Chr 16 68279247-68294961 (+)
        Pathway: Glycerophospholipid metabolism - Homo sapiens (human) (database: KEGG)
        Pathway: Lysosome - Homo sapiens (human) (database: KEGG)
        Pathway: Phospholipid Biosynthesis (database: SMPDB)
        Pathway: Glycerophospholipid metabolism (database: EHMN)
        Protein: PAG15 Group XV phospholipase A2;
            KO: K06129 lysophospholipase III [EC:3.1.1.5]
                ReactionKEGG: 1-Acyl-sn-glycero-3-phosphocholine + H2O <=> sn-glycero-3-Phosphocholine + Fatty acid
        OMIM: *609362 LYSOPHOSPHOLIPASE 3; LYPLA3

Distance to locus: -30126
    Gene: 84138 (SLC7A6OS) Chr 16 68334518-68344868 (-)
        Protein: S7A6O Probable RNA polymerase II nuclear localization protein SLC7A6OS;

Distance to locus: 34256
    Gene: 80004 (ESRP2) Chr 16 68262450-68270136 (-)
        Protein: ESRP2 Epithelial splicing regulatory protein 2;
            KO: K14947 epithelial splicing regulatory protein 1/2
        OMIM: *612960 EPITHELIAL SPLICING REGULATORY PROTEIN 2; ESRP2

Distance to locus: -40485
    Gene: 54496 (PRMT7) Chr 16 68344877-68391169 (+)
        Protein: ANM7 Protein arginine N-methyltransferase 7;
            KO: K11438 protein arginine N-methyltransferase 7 [EC:2.1.1.-]
            EC/TCDB: EC:2.1.1.125 Histone-arginine N-methyltransferase.
            EC/TCDB: EC:2.1.1.126 [Myelin basic protein]-arginine N-methyltransferase.
        OMIM: *610087 PROTEIN ARGININE N-METHYLTRANSFERASE 7; PRMT7

Distance to locus: 41230
    Gene: 4775 (NFATC3) Chr 16 68119269-68263162 (+)
        Pathway: T cell receptor signaling pathway - Homo sapiens (human) (database: KEGG)
        Pathway: B cell receptor signaling pathway - Homo sapiens (human) (database: KEGG)
        Pathway: Wnt signaling pathway - Homo sapiens (human) (database: KEGG)
        Pathway: HTLV-I infection - Homo sapiens (human) (database: KEGG)
        Pathway: Axon guidance - Homo sapiens (human) (database: KEGG)
        Pathway: VEGF signaling pathway - Homo sapiens (human) (database: KEGG)
        Pathway: Natural killer cell mediated cytotoxicity - Homo sapiens (human) (database: KEGG)
        Pathway: Heart Development (database: Wikipathways)
        Pathway: B Cell Receptor Signaling Pathway (database: Wikipathways)
        Pathway: BCR (database: NetPath)
        Pathway: Role of Calcineurin-dependent NFAT signaling in lymphocytes (database: PID)
        Pathway: Calcium signaling in the CD4+ TCR pathway (database: PID)
        Pathway: Downstream signaling in naive CD8+ T cells (database: PID)
        Pathway: Validated transcriptional targets of AP1 family members Fra1 and Fra2

(database: PID)
Pathway: Calcineurin-regulated NFAT-dependent transcription in lymphocytes (database: PID)
Pathway: AP-1 transcription factor network (database: PID)
Pathway: FOXM1 transcription factor network (database: PID)
Protein: NFAC3 Nuclear factor of activated T-cells, cytoplasmic 3;
KO: K04446 nuclear factor of activated T-cells, cytoplasmic
OMIM: *602698 NUCLEAR FACTOR OF ACTIVATED T CELLS, CYTOPLASMIC, CALCINEURIN-DEPENDENT3; NFATC3

Distance to locus: 60665
Gene: 100271552 (RPS12P27) Chr 16 68243284-68243727 (+)

Distance to locus: -87838
Gene: 55512 (SMPD3) Chr 16 68392230-68482409 (-)
Pathway: Metabolic pathways - Homo sapiens (human) (database: KEGG)
ReactionKEGG: N6-(L-1,3-Dicarboxypropyl)-L-lysine + NADP+ + H2O <=> L-Lysine + 2-Oxoglutarate + NADPH + H+
ReactionKEGG: Biocytin + H2O <=> Biotin + L-Lysine
Pathway: Sphingolipid metabolism - Homo sapiens (human) (database: KEGG)
Pathway: Glycosphingolipid metabolism (database: EHMN)
Pathway: Metabolism (database: Reactome)
ReactionKEGG: N6-(L-1,3-Dicarboxypropyl)-L-lysine + NADP+ + H2O <=> L-Lysine + 2-Oxoglutarate + NADPH + H+
Pathway: Phospholipid metabolism (database: Reactome)
Pathway: sphingomyelin metabolism (database: HumanCyc)
Pathway: Ceramide signaling pathway (database: PID)
Pathway: Metabolism of lipids and lipoproteins (database: Reactome)
Pathway: Glycosphingolipid metabolism (database: Reactome)
Pathway: Sphingolipid metabolism (database: Reactome)
Protein: NSMA2 Sphingomyelin phosphodiesterase 3;
KO: K12352 sphingomyelin phosphodiesterase 3 [EC:3.1.4.12]
ReactionKEGG: Sphingomyelin + H2O <=> N-Acylsphingosine + Choline phosphate
EC/TCDB: EC:3.1.4.12 Sphingomyelin phosphodiesterase.
ReactionHepatoNet1: H2O_cyto + SM-pool_cyto <=> Phosphocholine_cyto + Ceramide-pool_cyto
OMIM: *605777 SPHINGOMYELIN PHOSPHODIESTERASE 3, NEUTRAL MEMBRANE; SMPD3

Distance to locus: 191208
Gene: 54920 (DUS2L) Chr 16 68057204-68113184 (+)
Protein: DUS2L tRNA-dihydrouridine(20) synthase [NAD(P)+]-like;
KO: K05543 tRNA-dihydrouridine synthase 2 [EC:1.3.1.91]
OMIM: *609707 DIHYDROURIDINE SYNTHASE 2-LIKE; DUS2L

Distance to locus: -206703
Gene: 100271634 (RPL35AP33) Chr 16 68511095-68511562 (-)

Distance to locus: 246622
Gene: 55794 (DDX28) Chr 16 68055177-68057770 (-)
Protein: DDX28 Probable ATP-dependent RNA helicase DDX28;
EC/TCDB: EC:3.6.4.13 RNA helicase.
OMIM: *607618 DEAD/H BOX 28; DDX28

Distance to locus: 266038
Gene: 100132079 (KARSP3) Chr 16 68036326-68038354 (-)

Distance to locus: -269269
Gene: 146198 (ZFP90) Chr 16 68573661-68601039 (+)
Pathway: Gene Expression (database: Reactome)
ReactionKEGG: ATP + L-Lysine + tRNA(Lys) <=> AMP + Diphosphate + L-Lysyl-tRNA
Pathway: Generic Transcription Pathway (database: Reactome)
Protein: ZFP90 Zinc finger protein 90 homolog;
KO: K09228 KRAB domain-containing zinc finger protein
OMIM: *609451 ZINC FINGER PROTEIN 90, MOUSE, HOMOLOG OF; ZFP90

Distance to locus: 271028
Gene: 64174 (DPEP2) Chr 16 68021293-68033364 (-)
Pathway: Busulfan Pathway, Pharmacodynamics (database: PharmGKB)
Pathway: leukotriene biosynthesis (database: HumanCyc)
Pathway: Metabolism (database: Reactome)
ReactionKEGG: N6-(L-1,3-Dicarboxypropyl)-L-lysine + NADP+ + H2O <=> L-Lysine + 2-Oxoglutarate + NADPH + H+

Pathway: Arachidonic acid metabolism (database: Reactome)
Pathway: Metabolism of lipids and lipoproteins (database: Reactome)
Pathway: Leukotriene synthesis (database: PID)
Protein: DPEP2 Dipeptidase 2;
EC/TCDB: EC:3.4.13.19 Membrane dipeptidase.
OMIM: *609925 DIPEPTIDASE 2; DPEP2

Distance to locus: 289940
Gene: 64180 (DPEP3) Chr 16 68009566-68014452 (-)
Pathway: Busulfan Pathway, Pharmacodynamics (database: PharmGKB)
Protein: DPEP3 Dipeptidase 3;
EC/TCDB: EC:3.4.13.19 Membrane dipeptidase.
OMIM: *609926 DIPEPTIDASE 3; DPEP3

Distance to locus: 301795
Gene: 6560 (SLC12A4) Chr 16 67977377-68002597 (-)
Pathway: Epithelium TarBase (database: Wikipathways)
Pathway: Lymphocyte TarBase (database: Wikipathways)
Pathway: Muscle cell TarBase (database: Wikipathways)
Pathway: Cation-coupled Chloride cotransporters (database: Reactome)
Pathway: SLC-mediated transmembrane transport (database: Reactome)
Pathway: Transport of inorganic cations/anions and amino acids/oligopeptides (database: Reactome)
Pathway: Transmembrane transport of small molecules (database: Reactome)
Protein: S12A4 Solute carrier family 12 member 4;
KO: K14427 solute carrier family 12 (potassium/chloride transporter), member 4/5/6
OMIM: *604119 SOLUTE CARRIER FAMILY 12 (POTASSIUM/CHLORIDE TRANSPORTER), MEMBER4; SLC12A4

Distance to locus: 326377
Gene: 3931 (LCAT) Chr 16 67973787-67978015 (-)
Pathway: Glycerophospholipid metabolism - Homo sapiens (human) (database: KEGG)
Pathway: Statin Pathway, Pharmacodynamics (database: PharmGKB)
Pathway: Statin Pathway (database: Wikipathways)
Pathway: Metabolism (database: Reactome)
ReactionKEGG: N6-(L-1,3-Dicarboxypropyl)-L-lysine + NADP+ + H2O <=> L-Lysine + 2-Oxoglutarate + NADPH + H+
Pathway: HDL-mediated lipid transport (database: Reactome)
Pathway: Glycerophospholipid metabolism (database: EHMN)
Pathway: Metabolism of lipids and lipoproteins (database: Reactome)
Pathway: HDL-mediated lipid transport (database: PID)
Pathway: Lipoprotein metabolism (database: Reactome)
Pathway: Lipid digestion, mobilization, and transport (database: Reactome)
Protein: LCAT Phosphatidylcholine-sterol acyltransferase;
KO: K00650 lecithin-cholesterol acyltransferase [EC:2.3.1.43]
ReactionKEGG: Phosphatidylcholine + Sterol <=> 1-Acyl-sn-glycero-3-phosphocholine + Steryl ester
EC/TCDB: EC:2.3.1.43 Phosphatidylcholine--sterol O-acyltransferase.
OMIM: *606967 LECITHIN:CHOLESTEROL ACYLTRANSFERASE; LCAT
OMIM: #136120 FISH-EYE DISEASE; FED
OMIM: #245900 LECITHIN:CHOLESTEROL ACYLTRANSFERASE DEFICIENCY

Distance to locus: 333612
Gene: 5699 (PSMB10) Chr 16 67968407-67970780 (-)
Pathway: Proteasome - Homo sapiens (human) (database: KEGG)
Pathway: Proteasome Degradation (database: Wikipathways)
Pathway: IL-1 JNK (database: INOH)
Pathway: TGF-beta super family signaling pathway canonical (database: INOH)
Pathway: IL-1 NFkB (database: INOH)
Pathway: IL-1 p38 (database: INOH)
Pathway: JAK STAT pathway and regulation (database: INOH)
Pathway: B cell receptor signaling (database: INOH)
Pathway: Notch (database: INOH)
Pathway: Degradation of beta-catenin by the destruction complex (database: PID)
Pathway: TLR JNK (database: INOH)
Pathway: TLR NFkB (database: INOH)
Pathway: TLR p38 (database: INOH)
Pathway: TNF (database: INOH)
Pathway: Wnt Canonical (database: INOH)
Pathway: DroToll-like (database: INOH)
Pathway: CD4 T cell receptor signaling-NFkB cascade (database: INOH)
Pathway: CD4 T cell receptor signaling (database: INOH)
Pathway: Regulation of activated PAK-2p34 by proteasome mediated degradation (database: PID)
Pathway: APC/C:Cdc20 mediated degradation of Securin (database: PID)
Pathway: Vif-mediated degradation of APOBEC3G (database: PID)
Pathway: Host Interactions of HIV factors (database: PID)

Pathway: APC/C:Cdh1 mediated degradation of Cdc20 and other APC/C:Cdh1 targeted proteins in late mitosis/early G1 (database: PID)
Pathway: Regulation of activated PAK-2p34 by proteasome mediated degradation (database: PID)
Pathway: Ubiquitin-dependent degradation of Cyclin D1 (database: PID)
Pathway: Autodegradation of Cdh1 by Cdh1:APC/C (database: PID)
Pathway: Regulation of ornithine decarboxylase (ODC) (database: PID)
Pathway: SCF-beta-TrCP mediated degradation of Emi1 (database: PID)
Pathway: Autodegradation of the E3 ubiquitin ligase COP1 (database: PID)
Pathway: Removal of licensing factors from origins (database: PID)
Pathway: CDT1 association with the CDC6:ORC:origin complex (database: PID)
Pathway: Cdc20:Phospho-APC/C mediated degradation of Cyclin A (database: PID)
Pathway: Switching of origins to a post-replicative state (database: PID)
Pathway: Vpu mediated degradation of CD4 (database: PID)
Pathway: SCF(Skp2)-mediated degradation of p27/p21 (database: PID)
Pathway: Ubiquitin Mediated Degradation of Phosphorylated Cdc25A (database: PID)
Pathway: CDK-mediated phosphorylation and removal of Cdc6 (database: PID)
Pathway: Orc1 removal from chromatin (database: PID)
Pathway: Hedgehog (database: INOH)
Pathway: Wnt Mammals (database: INOH)
Protein: PSB10 Proteasome subunit beta type-10;
KO: K02733 20S proteasome subunit beta 10 [EC:3.4.25.1]
EC/TCDB: EC:3.4.25.1 Proteasome endopeptidase complex.
OMIM: *176847 PROTEASOME SUBUNIT, BETA-TYPE, 10; PSMB10

Distance to locus: 338614
Gene: 1506 (CTRL) Chr 16 67963473-67965778 (-)
Pathway: Protein digestion and absorption - Homo sapiens (human) (database: KEGG)
Pathway: Pancreatic secretion - Homo sapiens (human) (database: KEGG)
Protein: CTRL Chymotrypsin-like protease CTRL-1;
KO: K09632 chymotrypsin-like protease [EC:3.4.21.-]
OMIM: *118888 CHYMOTRYPSIN-LIKE PROTEASE; CTRL

Distance to locus: 340811
Gene: 5681 (PSKH1) Chr 16 67927175-67963581 (+)
Pathway: mRNA processing (database: Wikipathways)
Protein: KPSH1 Serine/threonine-protein kinase H1;
KO: K08808 protein serine kinase H [EC:2.7.11.1]
EC/TCDB: EC:2.7.11.1 Non-specific serine/threonine protein kinase.
OMIM: *177015 PROTEIN SERINE KINASE H1; PSKH1

Distance to locus: -373759
Gene: 1001 (CDH3) Chr 16 68678151-68732957 (+)
Pathway: Cell adhesion molecules (CAMs) - Homo sapiens (human) (database: KEGG)
Pathway: EGFR1 (database: NetPath)
Pathway: Cell-Cell communication (database: Reactome)
Pathway: Cell junction organization (database: Reactome)
Pathway: Adherens junctions interactions (database: Reactome)
Pathway: Cell-cell junction organization (database: Reactome)
Protein: CADH3 Cadherin-3;
KO: K06796 cadherin 3, type 1, P-cadherin
OMIM: *114021 CADHERIN 3; CDH3
OMIM: #225280 EEM SYNDROME
OMIM: #601553 HYPOTRICHOSIS, CONGENITAL, WITH JUVENILE MACULAR DYSTROPHY; HJMD

Distance to locus: -380541
Gene: 100418868 (LOC100418868) Chr 16 68684933-68685852 (-)

Distance to locus: 384121
Gene: 123904 (NRN1L) Chr 16 67918781-67920271 (+)
Protein: NRN1L Neuritin-like protein;

Distance to locus: 385986
Gene: 23644 (EDC4) Chr 16 67906999-67918406 (+)
Pathway: RNA degradation - Homo sapiens (human) (database: KEGG)
Pathway: mRNA Decay by 5' to 3' Exoribonuclease (database: PID)
Pathway: Gene Expression (database: Reactome)
ReactionKEGG: ATP + L-Lysine + tRNA(Lys) <=> AMP + Diphosphate + L-Lysyl-tRNA
Pathway: Metabolism of RNA (database: Reactome)
Pathway: Metabolism of mRNA (database: Reactome)
Pathway: mRNA Decay by 5, to 3, Exoribonuclease (database: Reactome)
Pathway: Deadenylation-dependent mRNA decay (database: Reactome)
Protein: EDC4 Enhancer of mRNA-decapping protein 4;
KO: K12616 enhancer of mRNA-decapping protein 4

OMIM: *606030 ENHANCER OF mRNA DECAPPING 4; EDC4

Distance to locus: 399173
Gene: 10204 (NUTF2)   Chr 16   67880819-67905219 (+)
Pathway: mechanism of protein import into the nucleus   (database: BioCarta)
Pathway: mechanism of protein import into the nucleus   (database: PID)
Protein: NTF2   Nuclear transport factor 2;
OMIM: *605813 NUCLEAR TRANSPORT FACTOR 2

Distance to locus: -415890
Gene: 100506000 (LOC100506000)   Chr 16   68720282-68720589 (+)

Distance to locus: 423031
Gene: 80152 (CENPT)   Chr 16   67862060-67881361 (-)
Pathway: Cell Cycle, Mitotic   (database: Reactome)
Pathway: Cell Cycle   (database: Reactome)
Pathway: Mitotic Prometaphase   (database: Reactome)
Pathway: M Phase   (database: Reactome)
Pathway: Mitotic M-M/G1 phases   (database: Reactome)
Pathway: DNA Replication   (database: Reactome)
Pathway: Mitotic Prometaphase   (database: PID)
Protein: CENPT   Centromere protein T;
KO: K11512   centromere protein T
OMIM: *611510 CENTROMERIC PROTEIN T; CENPT

Distance to locus: 426294
Gene: 57215 (THAP11)   Chr 16   67876213-67878098 (+)
Protein: THA11   THAP domain-containing protein 11;
OMIM: *609119 THAP DOMAIN-CONTAINING PROTEIN 11; THAP11

Distance to locus: 442421
Gene: 55815 (TSNAXIP1)   Chr 16   67841010-67861971 (+)
Protein: TXIP1   Translin-associated factor X-interacting protein 1;
OMIM: *607720 TRANSLIN-ASSOCIATED FACTOR X-INTERACTING PROTEIN 1; TSNAXIP1

Distance to locus: 463837
Gene: 57610 (RANBP10)   Chr 16   67757005-67840555 (-)
Pathway: Signaling events mediated by Hepatocyte Growth Factor Receptor (c-Met) (database: PID)
Protein: RBP10   Ran-binding protein 10;
OMIM: *614031 RAN-BINDING PROTEIN 10; RANBP10

Distance to locus: -466803
Gene: 999 (CDH1)   Chr 16   68771195-68869444 (+)
Pathway: Cell adhesion molecules (CAMs) - Homo sapiens (human)   (database: KEGG)
Pathway: Adherens junction - Homo sapiens (human)   (database: KEGG)
Pathway: Melanoma - Homo sapiens (human)   (database: KEGG)
Pathway: Bladder cancer - Homo sapiens (human)   (database: KEGG)
Pathway: Bacterial invasion of epithelial cells - Homo sapiens (human)   (database: KEGG)
Pathway: Pathogenic Escherichia coli infection - Homo sapiens (human)   (database: KEGG)
Pathway: Pathways in cancer - Homo sapiens (human)   (database: KEGG)
Pathway: Thyroid cancer - Homo sapiens (human)   (database: KEGG)
Pathway: Endometrial cancer - Homo sapiens (human)   (database: KEGG)
Pathway: Apoptotic execution phase   (database: Wikipathways)
Pathway: Cell cycle   (database: Wikipathways)
Pathway: Immunoregulatory interactions between a Lymphoid and a non-Lymphoid cell (database: Wikipathways)
Pathway: Integrin cell surface interactions   (database: Wikipathways)
Pathway: Integrated Breast Cancer Pathway   (database: Wikipathways)
Pathway: Neural Crest Differentiation   (database: Wikipathways)
Pathway: Pathogenic Escherichia coli infection   (database: Wikipathways)
Pathway: EGFR1   (database: NetPath)
Pathway: Signaling events mediated by Hepatocyte Growth Factor Receptor (c-Met) (database: PID)
Pathway: Integrin cell surface interactions   (database: Reactome)
Pathway: Signal Transduction   (database: Reactome)
Pathway: sumoylation as a mechanism to modulate ctbp-dependent gene responses (database: BioCarta)
Pathway: Immunoregulatory interactions between a Lymphoid and a non-Lymphoid cell (database: Reactome)
Pathway: Adaptive Immune System   (database: Reactome)
Pathway: Regulation of nuclear beta catenin signaling and target gene transcription (database: PID)
Pathway: tgf beta signaling pathway   (database: BioCarta)

Pathway: Wnt (database: NetPath)
Pathway: Apoptotic cleavage of cell adhesion proteins (database: Reactome)
Pathway: Apoptotic cleavage of cellular proteins (database: Reactome)
Pathway: Cell-Cell communication (database: Reactome)
Pathway: Cell junction organization (database: Reactome)
Pathway: Immune System (database: Reactome)
Pathway: Posttranslational regulation of adherens junction stability and dissassembly (database: PID)
Pathway: E-cadherin signaling in the nascent adherens junction (database: PID)
Pathway: Nectin adhesion pathway (database: PID)
Pathway: E-cadherin signaling in keratinocytes (database: PID)
Pathway: Integrin cell surface interactions (database: PID)
Pathway: CDC42 signaling events (database: PID)
Pathway: sumoylation as a mechanism to modulate ctbp-dependent gene responses (database: PID)
Pathway: RAC1 signaling pathway (database: PID)
Pathway: AlphaE beta7 integrin cell surface interactions (database: PID)
Pathway: Adherens junctions interactions (database: Reactome)
Pathway: Cell-cell junction organization (database: Reactome)
Pathway: tgf beta signaling pathway (database: PID)
Pathway: Apoptotic cleavage of cell adhesion proteins (database: PID)
Pathway: a6b1 and a6b4 Integrin signaling (database: PID)
Pathway: Apoptotic execution phase (database: Reactome)
Pathway: Apoptosis (database: Reactome)
Pathway: Immunoregulatory interactions between a Lymphoid and a non-Lymphoid cell (database: PID)
Pathway: Stabilization and expansion of the E-cadherin adherens junction (database: PID)
Pathway: downregulated of mta-3 in er-negative breast tumors (database: PID)
Pathway: Arf6 trafficking events (database: PID)
Pathway: FGF signaling pathway (database: PID)
Pathway: downregulated of mta-3 in er-negative breast tumors (database: BioCarta)
Protein: CADH1 Cadherin-1;
KO: K05689 cadherin 1, type 1, E-cadherin
OMIM: *192090 CADHERIN 1; CDH1
OMIM: #137215 GASTRIC CANCER, HEREDITARY DIFFUSE; HDGC
OMIM: #167000 OVARIAN CANCER OVARIAN CANCER, EPITHELIAL, INCLUDED

GWAS catalog
----

rs6499165 Chr 16 68326200 (-21808) SLC7A6 Metabolic traits (glutaroyl carnitine/lysine + 3 other traits) [Suhre K, 08/31/2011, Nature]
rs7197653 Chr 16 68383047 (-78655) PRMT7 Magnesium levels [Meyer TE, 08/05/2010, PLoS Genet]
rs255052 Chr 16 68024995 (279397) LCAT HDL cholesterol [Willer CJ, 01/13/2008, Nat Genet]
rs1728785 Chr 16 68591230 (-286838) ZFP90 Ulcerative colitis [Jostins L, 11/01/2012, Nature]
rs1728785 Chr 16 68591230 (-286838) CDH1 Ulcerative colitis [Barrett JC, 11/15/2009, Nat Genet]
rs255049 Chr 16 68013471 (290921) LCAT HDL cholesterol [Sabatti C, 12/07/2008, Nat Genet]
rs17689437 Chr 16 68600351 (-295959) ZFP90 HIV-1 viral setpoint [Lingappa JR, 12/12/2011, PLoS One]
rs17689437 Chr 16 68600351 (-295959) ZFP90 Reasoning (risperidone) [McClay JL, 11/24/2010, Neuropsychopharmacology]
rs11574514 Chr 16 67971380 (333012) PSMB10 Crohn's disease [Kenny EE, 03/08/2012, PLoS Genet]
rs6499188 Chr 16 68674788 (-370396) ZFP90 Ulcerative colitis [Anderson CA, 02/06/2011, Nat Genet]
rs16942887 Chr 16 67928042 (376350) LCAT HDL cholesterol [Teslovich TM, 08/05/2010, Nature]
rs8060686 Chr 16 67911517 (392875) EDC4 Metabolic syndrome (HDL) [Kristiansson K, 03/07/2012, Circ Cardiovasc Genet]
rs8060686 Chr 16 67911517 (392875) EDC4 Coronary heart disease (HDL-C) [Lettre G, 02/10/2011, PLoS Genet]
rs2271293 Chr 16 67902070 (402322) CTCF, PRMT8 HDL cholesterol [Aulchenko YS, 12/07/2008, Nat Genet]
rs2271293 Chr 16 67902070 (402322) LCAT HDL cholesterol [Kathiresan S, 12/07/2008, Nat Genet]

----
eQTL mapping (NCBI-GTEx database)

LD r^2	rs8056893	Gene	distance
rs9788810	0.51324	CDH1	-25605
rs10852440	0.53859	SLC7A6	-12669
rs35316276	0.60154	ATP6V0D1	453692
rs9937560	0.32178	CTCF	-130633
rs9937560	0.32178	CTCF	-130633
rs9788810	0.51324	PRMT7	-25605
rs3743742	0.45189	PRMT7	-40644
rs13336173	0.45189	PRMT7	-41299
rs4783618	0.43367	PRMT7	-52027
rs2290698	0.45338	PRMT7	-58417
rs1111574	0.42892	PRMT7	-74165
rs3826166	0.35218	PRMT7	-84389
rs3785129	0.37104	PRMT7	-96985
rs1868157	0.38136	PRMT7	-99262
rs2166768	0.35218	PRMT7	-102515
rs2166767	0.35218	PRMT7	-102804
rs3760001	0.3447	PRMT7	-103062
rs12917815	0.51015	C16orf48	-193063
rs7184977	0.32764	C16orf48	-201395

Mapping eQTL-SNP rs9788810 Chr 16 68329997 p-value = 0.00232 tissue = Liver
Gene: 999 (CDH1) Chr 16 68771195-68869444 (+)
Pathway: Cell adhesion molecules (CAMs) - Homo sapiens (human) (database: KEGG)
Pathway: Adherens junction - Homo sapiens (human) (database: KEGG)
Pathway: Melanoma - Homo sapiens (human) (database: KEGG)
Pathway: Bladder cancer - Homo sapiens (human) (database: KEGG)
Pathway: Bacterial invasion of epithelial cells - Homo sapiens (human) (database: KEGG)
Pathway: Pathogenic Escherichia coli infection - Homo sapiens (human) (database: KEGG)
Pathway: Pathways in cancer - Homo sapiens (human) (database: KEGG)
Pathway: Thyroid cancer - Homo sapiens (human) (database: KEGG)
Pathway: Endometrial cancer - Homo sapiens (human) (database: KEGG)
Pathway: Apoptotic execution phase (database: Wikipathways)
Pathway: Cell cycle (database: Wikipathways)
Pathway: Immunoregulatory interactions between a Lymphoid and a non-Lymphoid cell (database: Wikipathways)
Pathway: Integrin cell surface interactions (database: Wikipathways)
Pathway: Integrated Breast Cancer Pathway (database: Wikipathways)
Pathway: Neural Crest Differentiation (database: Wikipathways)
Pathway: Pathogenic Escherichia coli infection (database: Wikipathways)
Pathway: EGFR1 (database: NetPath)
Pathway: Signaling events mediated by Hepatocyte Growth Factor Receptor (c-Met) (database: PID)
Pathway: Integrin cell surface interactions (database: Reactome)
Pathway: Signal Transduction (database: Reactome)
Pathway: sumoylation as a mechanism to modulate ctbp-dependent gene responses (database: BioCarta)
Pathway: Immunoregulatory interactions between a Lymphoid and a non-Lymphoid cell (database: Reactome)
Pathway: Adaptive Immune System (database: Reactome)
Pathway: Regulation of nuclear beta catenin signaling and target gene transcription (database: PID)
Pathway: tgf beta signaling pathway (database: BioCarta)
Pathway: Wnt (database: NetPath)
Pathway: Apoptotic cleavage of cell adhesion proteins (database: Reactome)
Pathway: Apoptotic cleavage of cellular proteins (database: Reactome)
Pathway: Cell-Cell communication (database: Reactome)
Pathway: Cell junction organization (database: Reactome)
Pathway: Immune System (database: Reactome)
Pathway: Posttranslational regulation of adherens junction stability and dissassembly (database: PID)
Pathway: E-cadherin signaling in the nascent adherens junction (database: PID)
Pathway: Nectin adhesion pathway (database: PID)
Pathway: E-cadherin signaling in keratinocytes (database: PID)
Pathway: Integrin cell surface interactions (database: PID)
Pathway: CDC42 signaling events (database: PID)
Pathway: sumoylation as a mechanism to modulate ctbp-dependent gene responses (database: PID)
Pathway: RAC1 signaling pathway (database: PID)
Pathway: AlphaE beta7 integrin cell surface interactions (database: PID)
Pathway: Adherens junctions interactions (database: Reactome)
Pathway: Cell-cell junction organization (database: Reactome)
Pathway: tgf beta signaling pathway (database: PID)
Pathway: Apoptotic cleavage of cell adhesion proteins (database: PID)
Pathway: a6b1 and a6b4 Integrin signaling (database: PID)
Pathway: Apoptotic execution phase (database: Reactome)

Pathway: Apoptosis (database: Reactome)
Pathway: Immunoregulatory interactions between a Lymphoid and a non-Lymphoid cell (database: PID)
Pathway: Stabilization and expansion of the E-cadherin adherens junction (database: PID)
Pathway: downregulated of mta-3 in er-negative breast tumors (database: PID)
Pathway: Arf6 trafficking events (database: PID)
Pathway: FGF signaling pathway (database: PID)
Pathway: downregulated of mta-3 in er-negative breast tumors (database: BioCarta)
Protein: CADH1 Cadherin-1;
KO: K05689 cadherin 1, type 1, E-cadherin
OMIM: *192090 CADHERIN 1; CDH1
OMIM: #137215 GASTRIC CANCER, HEREDITARY DIFFUSE; HDGC
OMIM: #167000 OVARIAN CANCER OVARIAN CANCER, EPITHELIAL, INCLUDED

Mapping eQTL-SNP rs10852440 Chr 16 68317061 p-value = 0.0001343 tissue = Lymphoblastoid
Gene: 9057 (SLC7A6) Chr 16 68298423-68335722 (+)
Pathway: Lysinuric Protein Intolerance (database: SMPDB)
Pathway: Hartnup Disorder (database: SMPDB)
Pathway: Leukocyte TarBase (database: Wikipathways)
Pathway: Lymphocyte TarBase (database: Wikipathways)
Pathway: Muscle cell TarBase (database: Wikipathways)
Pathway: SLC-mediated transmembrane transport (database: Reactome)
Pathway: Amino acid transport across the plasma membrane (database: Reactome)
Pathway: Amino acid and oligopeptide SLC transporters (database: Reactome)
Pathway: Transport of inorganic cations/anions and amino acids/oligopeptides (database: Reactome)
Pathway: Transmembrane transport of small molecules (database: Reactome)
Pathway: Basigin interactions (database: Reactome)
Pathway: Glycine, serine, alanine and threonine metabolism (database: EHMN)
Pathway: Amino acid transport across the plasma membrane (database: PID)
Pathway: Cell surface interactions at the vascular wall (database: Reactome)
Pathway: Hemostasis (database: Reactome)
Protein: YLAT2 Y+L amino acid transporter 2;
KO: K13872 solute carrier family 7 (L-type amino acid transporter), member 6
OMIM: *605641 SOLUTE CARRIER FAMILY 7 (CATIONIC AMINO ACID TRANSPORTER, y+ SYSTEM),MEMBER 6; SLC7A6

Mapping eQTL-SNP rs35316276 Chr 16 67850700 p-value = 0.00019135 tissue = Lymphoblastoid
Gene: 9114 (ATP6V0D1) Chr 16 67471917-67515089 (-)
Pathway: Synaptic vesicle cycle - Homo sapiens (human) (database: KEGG)
Pathway: Vibrio cholerae infection - Homo sapiens (human) (database: KEGG)
Pathway: Phagosome - Homo sapiens (human) (database: KEGG)
Pathway: Lysosome - Homo sapiens (human) (database: KEGG)
Pathway: Epithelial cell signaling in Helicobacter pylori infection - Homo sapiens (human) (database: KEGG)
Pathway: Metabolic pathways - Homo sapiens (human) (database: KEGG)
ReactionKEGG: N6-(L-1,3-Dicarboxypropyl)-L-lysine + NADP+ + H2O <=> L-Lysine + 2-Oxoglutarate + NADPH + H+
ReactionKEGG: Biocytin + H2O <=> Biotin + L-Lysine
Pathway: Oxidative phosphorylation - Homo sapiens (human) (database: KEGG)
Pathway: Tuberculosis - Homo sapiens (human) (database: KEGG)
Pathway: Collecting duct acid secretion - Homo sapiens (human) (database: KEGG)
Pathway: Viral carcinogenesis - Homo sapiens (human) (database: KEGG)
Pathway: Rheumatoid arthritis - Homo sapiens (human) (database: KEGG)
Pathway: Unfolded Protein Response (database: Wikipathways)
Pathway: Phagosomal maturation (early endosomal stage) (database: Reactome)
Pathway: Latent infection of Homo sapiens with Mycobacterium tuberculosis (database: Reactome)
Pathway: Signal Transduction (database: Reactome)
Pathway: Disease (database: Reactome)
Pathway: Transferrin endocytosis and recycling (database: Reactome)
Pathway: Iron uptake and transport (database: Reactome)
Pathway: Activation of Chaperones by IRE1alpha (database: PID)
Pathway: Transmembrane transport of small molecules (database: Reactome)
Pathway: Signaling by Insulin receptor (database: Reactome)
Pathway: Insulin receptor recycling (database: Reactome)
Protein: VA0D1 V-type proton ATPase subunit d 1;
KO: K02146 V-type H+-transporting ATPase subunit AC39 [EC:3.6.3.14]
EC/TCDB: TCDB:3.A.2.2.4 H+- or Na+-translocating F-type, V-type and A-type ATPase (F-ATPase) superfamily.
EC/TCDB: TCDB:3.A.2.2.4 H+- or Na+-translocating F-type, V-type and A-type ATPase (F-ATPase) superfamily.
EC/TCDB: TCDB:3.A.2.2.4 H+- or Na+-translocating F-type, V-type and A-type ATPase (F-ATPase) superfamily.
EC/TCDB: TCDB:3.A.2.2.4 H+- or Na+-translocating F-type, V-type and A-type ATPase (F-ATPase) superfamily.
EC/TCDB: TCDB:3.A.2.2.4 H+- or Na+-translocating F-type, V-type and A-type ATPase

(F-ATPase) superfamily.
EC/TCDB: TCDB:3.A.2.2.4   H+- or Na+-translocating F-type, V-type and A-type ATPase (F-ATPase) superfamily.
EC/TCDB: TCDB:3.A.2.2.4   H+- or Na+-translocating F-type, V-type and A-type ATPase (F-ATPase) superfamily.
EC/TCDB: TCDB:3.A.2.2.4   H+- or Na+-translocating F-type, V-type and A-type ATPase (F-ATPase) superfamily.
EC/TCDB: TCDB:3.A.2.2.4   H+- or Na+-translocating F-type, V-type and A-type ATPase (F-ATPase) superfamily.
EC/TCDB: TCDB:3.A.2.2.4   H+- or Na+-translocating F-type, V-type and A-type ATPase (F-ATPase) superfamily.
EC/TCDB: TCDB:3.A.2.2.4   H+- or Na+-translocating F-type, V-type and A-type ATPase (F-ATPase) superfamily.
EC/TCDB: TCDB:3.A.2.2.4   H+- or Na+-translocating F-type, V-type and A-type ATPase (F-ATPase) superfamily.
EC/TCDB: TCDB:3.A.2.2.4   H+- or Na+-translocating F-type, V-type and A-type ATPase (F-ATPase) superfamily.
OMIM: *607028 ATPase, H+ TRANSPORTING, LYSOSOMAL, 38-KD, V0 SUBUNIT D, ISOFORM 1;ATP6V0D1

Mapping eQTL-SNP   rs9937560   Chr 16   68435025   p-value = 0.00024849   tissue = Lymphoblastoid
Mapping eQTL-SNP   rs9937560   Chr 16   68435025   p-value = 0.00024849   tissue = Lymphoblastoid
Gene: 10664 (CTCF)   Chr 16   67596310-67673088 (+)
Pathway: SIDS Susceptibility Pathways   (database: Wikipathways)
Pathway: TGF_beta_Receptor   (database: NetPath)
Pathway: ctcf: first multivalent nuclear factor   (database: PID)
Pathway: ctcf: first multivalent nuclear factor   (database: BioCarta)
Protein: CTCF   Transcriptional repressor CTCF;
OMIM: *604167 CCCTC-BINDING FACTOR; CTCF

Mapping eQTL-SNP   rs9788810   Chr 16   68329997   p-value = 1.78e-006   tissue = Liver
Mapping eQTL-SNP   rs3743742   Chr 16   68345036   p-value = 4.9534e-009   tissue = Lymphoblastoid
Mapping eQTL-SNP   rs13336173   Chr 16   68345691   p-value = 5.042e-008   tissue = Lymphoblastoid
Mapping eQTL-SNP   rs4783618   Chr 16   68356419   p-value = 6.1052e-009   tissue = Lymphoblastoid
Mapping eQTL-SNP   rs2290698   Chr 16   68362809   p-value = 4.7555e-008   tissue = Lymphoblastoid
Mapping eQTL-SNP   rs1111574   Chr 16   68378557   p-value = 7.236e-008   tissue = Lymphoblastoid
Mapping eQTL-SNP   rs3826166   Chr 16   68388781   p-value = 5.6637e-008   tissue = Lymphoblastoid
Mapping eQTL-SNP   rs3785129   Chr 16   68401377   p-value = 1.0847e-008   tissue = Lymphoblastoid
Mapping eQTL-SNP   rs1868157   Chr 16   68403654   p-value = 6.9599e-008   tissue = Lymphoblastoid
Mapping eQTL-SNP   rs2166768   Chr 16   68406907   p-value = 3.0711e-008   tissue = Lymphoblastoid
Mapping eQTL-SNP   rs2166767   Chr 16   68407196   p-value = 4.7577e-008   tissue = Lymphoblastoid
Mapping eQTL-SNP   rs3760001   Chr 16   68407454   p-value = 4.5436e-008   tissue = Lymphoblastoid
Gene: 54496 (PRMT7)   Chr 16   68344877-68391169 (+)
Protein: ANM7   Protein arginine N-methyltransferase 7;
KO: K11438   protein arginine N-methyltransferase 7 [EC:2.1.1.-]
EC/TCDB: EC:2.1.1.125   Histone-arginine N-methyltransferase.
EC/TCDB: EC:2.1.1.126   [Myelin basic protein]-arginine N-methyltransferase.
OMIM: *610087 PROTEIN ARGININE N-METHYLTRANSFERASE 7; PRMT7

Mapping eQTL-SNP   rs12917815   Chr 16   68497455   p-value = 0.00027856   tissue = Lymphoblastoid
Mapping eQTL-SNP   rs7184977   Chr 16   68505787   p-value = 0.00013037   tissue = Lymphoblastoid
Gene: 84080 (C16orf48)   Chr 16   67696850-67700628 (-)
Protein: CP048   Uncharacterized protein C16orf48;

Mapping SNP rs9896573 Chr 17 68139044

Distance to locus: 7295
    Gene: 3773 (KCNJ16) Chr 17 68071426-68131749 (+)
        Pathway: Gastric acid secretion - Homo sapiens (human) (database: KEGG)
        Pathway: Inhibition of voltage gated Ca2+ channels via Gbeta/gamma subunits (database: Reactome)
        Pathway: Activation of GABAB receptors (database: Reactome)
        Pathway: GABA B receptor activation (database: Reactome)
        Pathway: GABA receptor activation (database: Reactome)
        Pathway: Neurotransmitter Receptor Binding And Downstream Transmission In The Postsynaptic Cell (database: Reactome)
        Pathway: Transmission across Chemical Synapses (database: Reactome)
        Pathway: Neuronal System (database: Reactome)
        Pathway: Activation of G protein gated Potassium channels (database: Reactome)
        Pathway: G protein gated Potassium channels (database: Reactome)
        Pathway: Potassium transport channels (database: Reactome)
        Pathway: Inwardly rectifying K+ channels (database: Reactome)
        Pathway: Potassium Channels (database: Reactome)
        Protein: IRK16 Inward rectifier potassium channel 16;
            KO: K05009 potassium inwardly-rectifying channel subfamily J member 16
        OMIM: *605722 POTASSIUM CHANNEL, INWARDLY RECTIFYING, SUBFAMILY J, MEMBER 16; KCNJ16

Distance to locus: -24058
    Gene: 400617 (FLJ36644) Chr 17 68163102-68165543 (-)

Distance to locus: -26632
    Gene: 3759 (KCNJ2) Chr 17 68165676-68176185 (+)
        Pathway: Cholinergic synapse - Homo sapiens (human) (database: KEGG)
        Pathway: Gastric acid secretion - Homo sapiens (human) (database: KEGG)
        Pathway: Antiarrhythmic Pathway, Pharmacodynamics (database: PharmGKB)
        Pathway: Disopyramide Pathway (database: SMPDB)
        Pathway: Procainamide (Antiarrhythmic) Pathway (database: SMPDB)
        Pathway: Quinidine Pathway (database: SMPDB)
        Pathway: TarBasePathway (database: Wikipathways)
        Pathway: Epithelium TarBase (database: Wikipathways)
        Pathway: Inhibition of voltage gated Ca2+ channels via Gbeta/gamma subunits (database: Reactome)
        Pathway: Activation of GABAB receptors (database: Reactome)
        Pathway: GABA B receptor activation (database: Reactome)
        Pathway: GABA receptor activation (database: Reactome)
        Pathway: Neurotransmitter Receptor Binding And Downstream Transmission In The Postsynaptic Cell (database: Reactome)
        Pathway: Transmission across Chemical Synapses (database: Reactome)
        Pathway: Neuronal System (database: Reactome)
        Pathway: Activation of G protein gated Potassium channels (database: Reactome)
        Pathway: G protein gated Potassium channels (database: Reactome)
        Pathway: Classical Kir channels (database: Reactome)
        Pathway: Inwardly rectifying K+ channels (database: Reactome)
        Pathway: Potassium Channels (database: Reactome)
        Protein: IRK2 Inward rectifier potassium channel 2;
            KO: K04996 potassium inwardly-rectifying channel subfamily J member 2
            EC/TCDB: TCDB:1.A.2.1.2 inward rectifier K+ channel (IRK-C) family.
        OMIM: *600681 POTASSIUM CHANNEL, INWARDLY RECTIFYING, SUBFAMILY J, MEMBER 2; KCNJ2
            OMIM: #170390 ANDERSEN CARDIODYSRHYTHMIC PERIODIC PARALYSIS
            OMIM: #609620 SHORT QT SYNDROME 1; SQT1
            OMIM: #609622 SHORT QT SYNDROME 3; SQT3
            OMIM: #613980 ATRIAL FIBRILLATION, FAMILIAL, 9; ATFB9

Distance to locus: -98366
    Gene: 100128390 (CALM2P1) Chr 17 68237410-68238524 (+)

GWAS catalog
----

rs11867479 Chr 17 68090207 (48837) KCNJ16/KCNJ2 Height [Lango Allen H, 09/29/2010, Nature]
rs8079702 Chr 17 68190826 (-51782) KCNJ2 Primary tooth development (number of teeth) [Pillas D, 02/26/2010, PLoS Genet]
rs8079702 Chr 17 68190826 (-51782) KCNJ2 Primary tooth development (time to first

tooth eruption) [Pillas D, 02/26/2010, PLoS Genet]
rs623011 Chr 17 68259446 (-120402) KCNJ2 Thyrotoxic hypokalemic periodic paralysis [Jongjaroenprasert W, 03/08/2012, J Hum Genet]
rs6501384 Chr 17 68291133 (-152089) NR Eosinophilic esophagitis (pediatric) [Rothenberg ME, 03/07/2010, Nat Genet]
rs312691 Chr 17 68326338 (-187294) KCNJ16, KCNJ2 Thyrotoxic hypokalemic periodic paralysis [Cheung CL, 08/05/2012, Nat Genet]
rs2366017 Chr 17 67850901 (288143) KCNJ16 Palmitoleic acid (16:1n-7) plasma levels [Wu JH, 01/29/2013, Circ Cardiovasc Genet]
rs11870477 Chr 17 67802352 (336692) MAP2K6 Response to TNF-alpha inhibitors in rheumatoid arthritis (Change in DAS28) [Krintel SB, 05/06/2012, Pharmacogenet Genomics]
rs17779747 Chr 17 68494992 (-355948) KCNJ2 QT interval [Pfeufer A, 03/22/2009, Nat Genet]
rs7219669 Chr 17 68521861 (-382817) KCNJ2 Cardiac repolarization [Marjamaa A, 02/15/2012, Heart Rhythm]
rs16975985 Chr 17 68634065 (-495021) KCNJ2 Obesity-related traits (LDL) [Comuzzie AG, 12/04/2012, PLoS One]

Mapping SNP rs7422339 Chr 2 211540507

Distance to locus: 0
Gene: 1373 (CPS1) Chr 2 211342406-211543831 (+)
Pathway: Alanine, aspartate and glutamate metabolism - Homo sapiens (human) (database: KEGG)
ReactionKEGG: Glycine + 2-Oxoglutarate <=> Glyoxylate + L-Glutamate
ReactionKEGG: L-Alanine + Glyoxylate <=> Pyruvate + Glycine
ReactionKEGG: L-Serine + Glyoxylate <=> Hydroxypyruvate + Glycine
Pathway: Arginine and proline metabolism - Homo sapiens (human) (database: KEGG)
ReactionKEGG: Glycine + H2O + Oxygen <=> Glyoxylate + NH3 + Hydrogen peroxide
ReactionKEGG: Cys-Gly + H2O <=> L-Cysteine + Glycine
ReactionKEGG: R-S-Cysteinylglycine + H2O <=> S-Substituted L-cysteine + Glycine
ReactionKEGG: L-Arginine + Glycine <=> L-Ornithine + Guanidinoacetate
Pathway: Metabolic pathways - Homo sapiens (human) (database: KEGG)
ReactionKEGG: ATP + gamma-L-Glutamyl-L-cysteine + Glycine <=> ADP + Orthophosphate + Glutathione
ReactionKEGG: Glycine + 2-Oxoglutarate <=> Glyoxylate + L-Glutamate
ReactionKEGG: Glycine + H2O + Oxygen <=> Glyoxylate + NH3 + Hydrogen peroxide
ReactionKEGG: Sarcosine + H2O + Oxygen <=> Glycine + Formaldehyde + Hydrogen peroxide
ReactionKEGG: Choloyl-CoA + Glycine <=> CoA + Glycocholate
ReactionKEGG: L-Alanine + Glyoxylate <=> Pyruvate + Glycine
ReactionKEGG: 5,10-Methylenetetrahydrofolate + Glycine + H2O <=> Tetrahydrofolate + L-Serine
ReactionKEGG: Cys-Gly + H2O <=> L-Cysteine + Glycine
ReactionKEGG: Glycine + Tetrahydrofolate + NAD+ <=> 5,10-Methylenetetrahydrofolate + NH3 + CO2 + NADH + H+
ReactionKEGG: Sarcosine + Electron-transferring flavoprotein + H2O <=> Glycine + Formaldehyde + Reduced electron-transferring flavoprotein
ReactionKEGG: L-Serine + Glyoxylate <=> Hydroxypyruvate + Glycine
ReactionKEGG: Glycine + Lipoylprotein <=> S-Aminomethyldihydrolipoylprotein + CO2
ReactionKEGG: L-Serine + 5,6,7,8-Tetrahydromethanopterin <=> 5,10-Methylenetetrahydromethanopterin + Glycine + H2O
ReactionKEGG: ATP + 5-Phosphoribosylamine + Glycine <=> ADP + Orthophosphate + 5'-Phosphoribosylglycinamide
ReactionKEGG: R-S-Cysteinylglycine + H2O <=> S-Substituted L-cysteine + Glycine
ReactionKEGG: Chenodeoxycholoyl-CoA + Glycine <=> Glycochenodeoxycholate + CoA
ReactionKEGG: L-Arginine + Glycine <=> L-Ornithine + Guanidinoacetate
ReactionKEGG: Succinyl-CoA + Glycine <=> 5-Aminolevulinate + CoA + CO2
Pathway: Ammonia Recycling (database: SMPDB)
ReactionKEGG: Glycine + Tetrahydrofolate + NAD+ <=> 5,10-Methylenetetrahydrofolate + NH3 + CO2 + NADH + H+
Pathway: Carbamoyl Phosphate Synthetase Deficiency (database: SMPDB)
Pathway: Urea Cycle (database: SMPDB)
Pathway: Prolidase Deficiency(PD) (database: SMPDB)
ReactionKEGG: Glycine + H2O + Oxygen <=> Glyoxylate + NH3 + Hydrogen peroxide
ReactionKEGG: Cys-Gly + H2O <=> L-Cysteine + Glycine
ReactionKEGG: R-S-Cysteinylglycine + H2O <=> S-Substituted L-cysteine + Glycine
ReactionKEGG: L-Arginine + Glycine <=> L-Ornithine + Guanidinoacetate
Pathway: Arginine and Proline Metabolism (database: SMPDB)
ReactionKEGG: Glycine + H2O + Oxygen <=> Glyoxylate + NH3 + Hydrogen peroxide
ReactionKEGG: Cys-Gly + H2O <=> L-Cysteine + Glycine
ReactionKEGG: R-S-Cysteinylglycine + H2O <=> S-Substituted L-cysteine + Glycine
ReactionKEGG: L-Arginine + Glycine <=> L-Ornithine + Guanidinoacetate
Pathway: Metabolism of amino acids and derivatives (database: Wikipathways)
Pathway: Urea cycle and metabolism of amino groups (database: Wikipathways)
ReactionKEGG: L-Arginine + Glycine <=> L-Ornithine + Guanidinoacetate
Pathway: Urea cycle and metabolism of arginine, proline, glutamate, aspartate and asparagine (database: EHMN)
ReactionKEGG: Glycine + H2O + Oxygen <=> Glyoxylate + NH3 + Hydrogen peroxide
ReactionKEGG: Cys-Gly + H2O <=> L-Cysteine + Glycine
ReactionKEGG: R-S-Cysteinylglycine + H2O <=> S-Substituted L-cysteine + Glycine
ReactionKEGG: L-Arginine + Glycine <=> L-Ornithine + Guanidinoacetate
Pathway: Pyrimidine nucleotides nucleosides metabolism (database: INOH)
Pathway: Metabolism of amino acids and derivatives (database: Reactome)
ReactionKEGG: Glycine + 2-Oxoglutarate <=> Glyoxylate + L-Glutamate
ReactionKEGG: Glycine + H2O + Oxygen <=> Glyoxylate + NH3 + Hydrogen peroxide
ReactionKEGG: L-Alanine + Glyoxylate <=> Pyruvate + Glycine
ReactionKEGG: 5,10-Methylenetetrahydrofolate + Glycine + H2O <=> Tetrahydrofolate + L-Serine
ReactionKEGG: L-Serine + Glyoxylate <=> Hydroxypyruvate + Glycine
ReactionKEGG: L-Serine + 5,6,7,8-Tetrahydromethanopterin <=> 5,10-Methylenetetrahydromethanopterin + Glycine + H2O
ReactionKEGG: L-Arginine + Glycine <=> L-Ornithine + Guanidinoacetate
Pathway: Metabolism (database: Reactome)
ReactionKEGG: ATP + gamma-L-Glutamyl-L-cysteine + Glycine <=> ADP + Orthophosphate + Glutathione
ReactionKEGG: Glycine + 2-Oxoglutarate <=> Glyoxylate + L-Glutamate
ReactionKEGG: Glycine + H2O + Oxygen <=> Glyoxylate + NH3 + Hydrogen peroxide
ReactionKEGG: Choloyl-CoA + Glycine <=> CoA + Glycocholate
ReactionKEGG: L-Alanine + Glyoxylate <=> Pyruvate + Glycine
ReactionKEGG: 5,10-Methylenetetrahydrofolate + Glycine + H2O <=> Tetrahydrofolate + L-Serine
ReactionKEGG: L-Serine + Glyoxylate <=> Hydroxypyruvate + Glycine
ReactionKEGG: Phenylacetyl-CoA + Glycine <=> Phenylacetylglycine + CoA
ReactionKEGG: L-Serine + 5,6,7,8-Tetrahydromethanopterin <=> 5,10-Methylenetetrahydromethanopterin + Glycine + H2O
ReactionKEGG: ATP + 5-Phosphoribosylamine + Glycine <=> ADP + Orthophosphate + 5'-Phosphoribosylglycinamide
ReactionKEGG: Chenodeoxycholoyl-CoA + Glycine <=> Glycochenodeoxycholate + CoA
ReactionKEGG: L-Arginine + Glycine <=> L-Ornithine + Guanidinoacetate
ReactionKEGG: Succinyl-CoA + Glycine <=> 5-Aminolevulinate + CoA + CO2
Pathway: Urea cycle (database: PID)
Pathway: Glutamate Glutamine metabolism (database: INOH)
ReactionKEGG: ATP + gamma-L-Glutamyl-L-cysteine + Glycine <=> ADP + Orthophosphate + Glutathione
Pathway: urea cycle (database: HumanCyc)
Pathway: Urea cycle (database: Reactome)
Pathway: superpathway of citrulline metabolism (database: HumanCyc)
Protein: CPSM Carbamoyl-phosphate synthase [ammonia], mitochondrial;
KO: K01948 carbamoyl-phosphate synthase (ammonia) [EC:6.3.4.16]
ReactionKEGG: 2 ATP + NH3 + CO2 + H2O <=> 2 ADP + Orthophosphate + Carbamoyl phosphate
R00149+R00575+R00372: CO2 + 2-Oxoglutarate + NH3 + Glycine <=> HCO3- + L-Glutamine + Glyoxylate
R00149+R01398+R00565: L-Arginine + H2O + CO2 + NH3 + Glycine + 2 ATP <=> 2 ADP + L-Citrulline + Guanidinoacetate + 2 Orthophosphate
EC/TCDB: EC:6.3.4.16 Carbamoyl-phosphate synthase (ammonia).

ReactionHepatoNet1: H2O_mito + 2 ATP_mito + CO2_mito + NH3_mito <=> 2 ADP_mito + Pi_mito + Carbamoyl-P_mito
OMIM: +608307 CARBAMOYL PHOSPHATE SYNTHETASE I; CPS1 VENOOCCLUSIVE DISEASE AFTER BONE MARROW TRANSPLANTATION, SUSCEPTIBILITYTO, INCLUDED
OMIM: #237300 CARBAMOYL PHOSPHATE SYNTHETASE I DEFICIENCY, HYPERAMMONEMIA DUE TO
OMIM: #615371 PULMONARY HYPERTENSION, NEONATAL, SUSCEPTIBILITY TO; PHN

Distance to locus: 55907
Gene: 29034 (CPS1-IT1) Chr 2 211482295-211484600 (+)

Distance to locus: 199008
Gene: 10314 (LANCL1) Chr 2 211295973-211341499 (-)
Protein: LANC1 LanC-like protein 1;
OMIM: *604155 LanC-LIKE 1; LANCL1

Distance to locus: 360612
Gene: 4632 (MYL1) Chr 2 211154868-211179895 (-)
Pathway: Intracellular Signalling Through Prostacyclin Receptor and Prostacyclin (database: SMPDB)
Pathway: Striated Muscle Contraction (database: Wikipathways)
Pathway: Regulation of Actin Cytoskeleton (database: Wikipathways)
Pathway: G13 Signaling Pathway (database: Wikipathways)
Pathway: Endothelin (database: Wikipathways)
Pathway: Striated Muscle Contraction (database: Reactome)
Pathway: Muscle contraction (database: Reactome)
Pathway: Regulation of retinoblastoma protein (database: PID)
Protein: MYL1 Myosin light chain 1/3, skeletal muscle isoform;
KO: K05738 myosin light chain 1
OMIM: *160780 MYOSIN, LIGHT POLYPEPTIDE 1, ALKALI, SKELETAL, FAST; MYL1

Distance to locus: -429144
Gene: 100420775 (LOC100420775) Chr 2 211969651-211970454 (+)

Distance to locus: 450292
Gene: 33 (ACADL) Chr 2 211052714-211090215 (-)
Pathway: Fatty acid metabolism - Homo sapiens (human) (database: KEGG)
Pathway: Metabolic pathways - Homo sapiens (human) (database: KEGG)
ReactionKEGG: ATP + gamma-L-Glutamyl-L-cysteine + Glycine <=> ADP + Orthophosphate + Glutathione
ReactionKEGG: Glycine + 2-Oxoglutarate <=> Glyoxylate + L-Glutamate
ReactionKEGG: Glycine + H2O + Oxygen <=> Glyoxylate + NH3 + Hydrogen peroxide
ReactionKEGG: Sarcosine + H2O + Oxygen <=> Glycine + Formaldehyde + Hydrogen peroxide
ReactionKEGG: Choloyl-CoA + Glycine <=> CoA + Glycocholate
ReactionKEGG: L-Alanine + Glyoxylate <=> Pyruvate + Glycine
ReactionKEGG: 5,10-Methylenetetrahydrofolate + Glycine + H2O <=> Tetrahydrofolate + L-Serine
ReactionKEGG: Cys-Gly + H2O <=> L-Cysteine + Glycine
ReactionKEGG: Glycine + Tetrahydrofolate + NAD+ <=> 5,10-Methylenetetrahydrofolate + NH3 + CO2 + NADH + H+
ReactionKEGG: Sarcosine + Electron-transferring flavoprotein + H2O <=> Glycine + Formaldehyde + Reduced electron-transferring flavoprotein
ReactionKEGG: L-Serine + Glyoxylate <=> Hydroxypyruvate + Glycine
ReactionKEGG: Glycine + Lipoylprotein <=> S-Aminomethyldihydrolipoylprotein + CO2
ReactionKEGG: L-Serine + 5,6,7,8-Tetrahydromethanopterin <=> 5,10-Methylenetetrahydromethanopterin + Glycine + H2O
ReactionKEGG: ATP + 5-Phosphoribosylamine + Glycine <=> ADP + Orthophosphate + 5'-Phosphoribosylglycinamide
ReactionKEGG: R-S-Cysteinylglycine + H2O <=> S-Substituted L-cysteine + Glycine
ReactionKEGG: Chenodeoxycholoyl-CoA + Glycine <=> Glycochenodeoxycholate + CoA
ReactionKEGG: L-Arginine + Glycine <=> L-Ornithine + Guanidinoacetate
ReactionKEGG: Succinyl-CoA + Glycine <=> 5-Aminolevulinate + CoA + CO2
Pathway: PPAR signaling pathway - Homo sapiens (human) (database: KEGG)
Pathway: Fatty acid Metabolism (database: SMPDB)
Pathway: Fatty Acid Beta Oxidation (database: Wikipathways)
Pathway: Mitochondrial LC-Fatty Acid Beta-Oxidation (database: Wikipathways)
Pathway: Valine, leucine and isoleucine degradation (database: EHMN)
Pathway: Propanoate metabolism (database: EHMN)
Pathway: Di-unsaturated fatty acid beta-oxidation (database: EHMN)
Pathway: Metabolism (database: Reactome)
ReactionKEGG: ATP + gamma-L-Glutamyl-L-cysteine + Glycine <=> ADP + Orthophosphate + Glutathione
ReactionKEGG: Glycine + 2-Oxoglutarate <=> Glyoxylate + L-Glutamate
ReactionKEGG: Glycine + H2O + Oxygen <=> Glyoxylate + NH3 + Hydrogen peroxide
ReactionKEGG: Choloyl-CoA + Glycine <=> CoA + Glycocholate
ReactionKEGG: L-Alanine + Glyoxylate <=> Pyruvate + Glycine
ReactionKEGG: 5,10-Methylenetetrahydrofolate + Glycine + H2O <=> Tetrahydrofolate + L-Serine
ReactionKEGG: L-Serine + Glyoxylate <=> Hydroxypyruvate + Glycine
ReactionKEGG: Phenylacetyl-CoA + Glycine <=> Phenylacetylglycine + CoA
ReactionKEGG: L-Serine + 5,6,7,8-Tetrahydromethanopterin <=> 5,10-Methylenetetrahydromethanopterin + Glycine + H2O
ReactionKEGG: ATP + 5-Phosphoribosylamine + Glycine <=> ADP + Orthophosphate + 5'-Phosphoribosylglycinamide
ReactionKEGG: Chenodeoxycholoyl-CoA + Glycine <=> Glycochenodeoxycholate + CoA
ReactionKEGG: L-Arginine + Glycine <=> L-Ornithine + Guanidinoacetate
ReactionKEGG: Succinyl-CoA + Glycine <=> 5-Aminolevulinate + CoA + CO2
Pathway: Beta oxidation of myristoyl-CoA to lauroyl-CoA (database: Reactome)
Pathway: Beta oxidation of lauroyl-CoA to decanoyl-CoA-CoA (database: Reactome)
Pathway: mitochondrial fatty acid beta-oxidation of saturated fatty acids (database: Reactome)
Pathway: Mitochondrial Fatty Acid Beta-Oxidation (database: Reactome)
Pathway: Fatty acid, triacylglycerol, and ketone body metabolism (database: Reactome)
Pathway: Dimethyl-branched-chain fatty acid mitochondrial beta-oxidation (database: EHMN)
Pathway: Mono-unsaturated fatty acid beta-oxidation (database: EHMN)
Pathway: Saturated fatty acids beta-oxidation (database: EHMN)
ReactionKEGG: Choloyl-CoA + Glycine <=> CoA + Glycocholate
ReactionKEGG: Chenodeoxycholoyl-CoA + Glycine <=> Glycochenodeoxycholate + CoA
Pathway: Metabolism of lipids and lipoproteins (database: Reactome)
ReactionKEGG: Choloyl-CoA + Glycine <=> CoA + Glycocholate
ReactionKEGG: Chenodeoxycholoyl-CoA + Glycine <=> Glycochenodeoxycholate + CoA
Pathway: Mitochondrial Fatty Acid Beta-Oxidation (database: PID)
Pathway: Beta oxidation of myristoyl-CoA to lauroyl-CoA (database: PID)

Pathway: mitochondrial fatty acid beta-oxidation of saturated fatty acids (database: PID)
Pathway: mitochondrial fatty acid beta-oxidation of unsaturated fatty acids (database: PID)
Pathway: Beta oxidation of lauroyl-CoA to decanoyl-CoA-CoA (database: PID)
Protein: ACADL Long-chain specific acyl-CoA dehydrogenase, mitochondrial;
KO: K00255 long-chain-acyl-CoA dehydrogenase [EC:1.3.8.8]
ReactionKEGG: Palmitoyl-CoA + FAD <=> trans-Hexadec-2-enoyl-CoA + FADH2
R01279+R01281+R00945: H2O + 5,10-Methylenetetrahydrofolate + Glycine + trans-Hexadec-2-enoyl-CoA + FADH2 <=> FAD + Tetrahydrofolate + CoA + 3-Dehydrosphinganine + CO2
R01279+R01281+R00588: FAD + CoA + 3-Dehydrosphinganine + CO2 + Glyoxylate <=> Glycine + Hydroxypyruvate + trans-Hexadec-2-enoyl-CoA + FADH2
R01279+R01281+R09099: 5,6,7,8-Tetrahydromethanopterin + FAD + CoA + 3-Dehydrosphinganine + CO2 <=> 5,10-Methylenetetrahydromethanopterin + H2O + Glycine + trans-Hexadec-2-enoyl-CoA + FADH2
ReactionKEGG: Octanoyl-CoA + FAD <=> trans-Oct-2-enoyl-CoA + FADH2
ReactionKEGG: Lauroyl-CoA + FAD <=> 2-trans-Dodecenoyl-CoA + FADH2
ReactionKEGG: Tetradecanoyl-CoA + FAD <=> trans-Tetradec-2-enoyl-CoA + FADH2
ReactionKEGG: Hexanoyl-CoA + FAD <=> trans-Hex-2-enoyl-CoA + FADH2
ReactionKEGG: Decanoyl-CoA + FAD <=> trans-Dec-2-enoyl-CoA + FADH2
EC/TCDB: EC:1.3.99.13 Long-chain-acyl-CoA dehydrogenase.
OMIM: *609576 ACYL-CoA DEHYDROGENASE, LONG-CHAIN; ACADL

GWAS catalog
----

rs7422339	Chr 2	211540507 (0)	CPS1	Chronic kidney disease (eGFRcrea)	[Kottgen A, 04/11/2010, Nat Genet]
rs7422339	Chr 2	211540507 (0)	CPS1	Homocysteine levels	[Lange LA, 02/13/2010, Hum Mol Genet]
rs7422339	Chr 2	211540507 (0)	CPS1	Fibrinogen	[Danik JS, 04/01/2009, Circ Cardiovasc Genet]
rs715	Chr 2	211543055 (-2548)	CPS1	Metabolite levels (glycine)	[Xie W, 02/01/2013, Diabetes]
rs2371030	Chr 2	211569722 (-29215)	CPS1	Non-small cell lung cancer (Additive model)	[Lee Y, 11/08/2012, Carcinogenesis]
rs2216405	Chr 2	211616894 (-76387)	CPS1	Metabolic traits (glycine + 17 other traits)	[Suhre K, 08/31/2011, Nature]
rs3764913	Chr 2	211074909 (465598)	ACADL	Metabolite levels (2,6 dimethylheptanoyl carnitine)	[Hong MG, 03/01/2013, Hum Mutat]
rs2286963	Chr 2	211060050 (480457)	ACADL	Metabolite levels (C9/C10:2)	[Illig T, 12/27/2009, Nat Genet]

Mapping SNP rs1922005   Chr 2   217733496

Distance to locus: 8714
    Gene: 7141 (TNP1)   Chr 2   217724181-217724782 (-)
        Protein: STP1   Spermatid nuclear transition protein 1;
        OMIM: *190231 TRANSITION PROTEIN 1; TNP1

Distance to locus: 82594
    Gene: 100129310 (RPL31P14)   Chr 2   217650458-217650902 (-)

Distance to locus: 173224
    Gene: 3488 (IGFBP5)   Chr 2   217536828-217560272 (-)
        Pathway: Regulation of Insulin-like Growth Factor (IGF) Activity by Insulin-like Growth Factor Binding Proteins (IGFBPs)   (database: Wikipathways)
        Pathway: Myometrial Relaxation and Contraction Pathways   (database: Wikipathways)
        Pathway: Senescence and Autophagy   (database: Wikipathways)
        Pathway: Disease   (database: Reactome)
            ReactionKEGG: (S)-Lactate + NAD+ <=> Pyruvate + NADH + H+
        Pathway: Regulation of Insulin-like Growth Factor (IGF) Activity by Insulin-like Growth Factor Binding Proteins (IGFBPs)   (database: Reactome)
        Pathway: Diabetes pathways   (database: Reactome)
        Pathway: Regulation of Insulin-like Growth Factor (IGF) Activity by Insulin-like Growth Factor Binding Proteins (IGFBPs)   (database: PID)
        Pathway: IGF signaling   (database: INOH)
        Protein: IBP5   Insulin-like growth factor-binding protein 5;
        OMIM: *146734 INSULIN-LIKE GROWTH FACTOR-BINDING PROTEIN 5; IGFBP5

Distance to locus: 204337
    Gene: 3485 (IGFBP2)   Chr 2   217498127-217529159 (+)
        Pathway: Regulation of Insulin-like Growth Factor (IGF) Activity by Insulin-like Growth Factor Binding Proteins (IGFBPs)   (database: Wikipathways)
        Pathway: Myometrial Relaxation and Contraction Pathways   (database: Wikipathways)
        Pathway: Disease   (database: Reactome)
            ReactionKEGG: (S)-Lactate + NAD+ <=> Pyruvate + NADH + H+
        Pathway: Regulation of Insulin-like Growth Factor (IGF) Activity by Insulin-like Growth Factor Binding Proteins (IGFBPs)   (database: Reactome)
        Pathway: Diabetes pathways   (database: Reactome)
        Pathway: Regulation of Insulin-like Growth Factor (IGF) Activity by Insulin-like Growth Factor Binding Proteins (IGFBPs)   (database: PID)
        Pathway: IGF signaling   (database: INOH)
        Protein: IBP2   Insulin-like growth factor-binding protein 2;
        OMIM: *146731 INSULIN-LIKE GROWTH FACTOR-BINDING PROTEIN 2; IGFBP2

Distance to locus: 257736
    Gene: 130700 (PSMB3P2)   Chr 2   217474994-217475760 (-)

Distance to locus: 367306
    Gene: 6168 (RPL37A)   Chr 2   217363520-217366190 (+)
        Pathway: Ribosome - Homo sapiens (human)   (database: KEGG)
        Pathway: Cytoplasmic Ribosomal Proteins   (database: Wikipathways)
        Pathway: L13a-mediated translational silencing of Ceruloplasmin expression   (database: Reactome)
        Pathway: Eukaryotic Translation Initiation   (database: Reactome)
        Pathway: SRP-dependent cotranslational protein targeting to membrane   (database: Reactome)
        Pathway: Nonsense Mediated Decay Enhanced by the Exon Junction Complex   (database: Reactome)
        Pathway: Nonsense Mediated Decay Independent of the Exon Junction Complex   (database: Reactome)
        Pathway: Nonsense-Mediated Decay   (database: Reactome)
        Pathway: Eukaryotic Translation Elongation   (database: Reactome)
        Pathway: Eukaryotic Translation Termination   (database: Reactome)
        Pathway: Translation   (database: Reactome)
        Pathway: Gene Expression   (database: Reactome)
        Pathway: Metabolism of proteins   (database: Reactome)
            ReactionKEGG: (R)-Lactate + 2 Ferricytochrome c <=> Pyruvate + 2 Ferrocytochrome c + 2 H+
        Pathway: Metabolism of RNA   (database: Reactome)
        Pathway: 3, -UTR-mediated translational regulation   (database: Reactome)
        Pathway: Metabolism of mRNA   (database: Reactome)
        Pathway: Peptide chain elongation   (database: PID)
        Pathway: L13a-mediated translational silencing of Ceruloplasmin expression   (database:

```
PID)
        Pathway: Regulation of gene expression in beta cells   (database: PID)
            ReactionKEGG:       dGTP + Pyruvate <=> dGDP + Phosphoenolpyruvate
            ReactionKEGG:       ATP + Pyruvate <=> ADP + Phosphoenolpyruvate
            ReactionKEGG:       Nucleoside triphosphate + Pyruvate <=> NDP + Phosphoenolpyruvate
            ReactionKEGG:       dATP + Pyruvate <=> dADP + Phosphoenolpyruvate
            ReactionKEGG:       GTP + Pyruvate <=> GDP + Phosphoenolpyruvate
        Pathway: GTP hydrolysis and joining of the 60S ribosomal subunit   (database: PID)
        Pathway: Formation of a pool of free 40S subunits   (database: PID)
        Pathway: Peptide chain elongation   (database: Reactome)
        Pathway: Eukaryotic Translation Initiation   (database: PID)
        Pathway: Cap-dependent Translation Initiation   (database: PID)
        Pathway: Eukaryotic Translation Termination   (database: PID)
        Pathway: Insulin Synthesis and Processing   (database: PID)
        Pathway: Viral mRNA Translation   (database: PID)
        Pathway: Formation of a pool of free 40S subunits   (database: Reactome)
        Pathway: GTP hydrolysis and joining of the 60S ribosomal subunit   (database: Reactome)
        Pathway: Cap-dependent Translation Initiation   (database: Reactome)
        Protein: RL37A   60S ribosomal protein L37a;
            KO: K02921   large subunit ribosomal protein L37Ae
        OMIM: *613314 RIBOSOMAL PROTEIN L37A; RPL37A


Distance to locus: 370027
    Gene: 100507554 (LOC100507554)   Chr 2   217351335-217363469  (-)


Distance to locus: 385720
    Gene: 50485 (SMARCAL1)   Chr 2   217277137-217347776  (+)
        Pathway: ATR signaling pathway   (database: PID)
        Protein: SMAL1   SWI/SNF-related matrix-associated actin-dependent regulator of chromatin
subfamily A-like protein 1;
            KO: K14440   SWI/SNF-related matrix-associated actin-dependent regulator of chromatin
subfamily A-like protein 1 [EC:3.6.4.12]
        OMIM: *606622 SWI/SNF-RELATED, MATRIX-ASSOCIATED, ACTIN-DEPENDENT REGULATOR OF
CHROMATIN,SUBFAMILY A-LIKE PROTEIN 1; SMARCAL1
            OMIM: #242900 IMMUNOOSSEOUS DYSPLASIA, SCHIMKE TYPE


Distance to locus: -415250
    Gene: 729582 (DIRC3)   Chr 2   218148746-218621316  (-)
        OMIM: *608262 DISRUPTED IN RENAL CARCINOMA 3; DIRC3DIRC3/HSPBAP1 FUSION GENE, INCLUDED


Distance to locus: 496746
    Gene: 57574 (MARCH4)   Chr 2   217122585-217236750  (-)
        Protein: MARH4   E3 ubiquitin-protein ligase MARCH4;
            KO: K10659   E3 ubiquitin-protein ligase MARCH4/9/11 [EC:6.3.2.19]
        OMIM: *608208 MEMBRANE-ASSOCIATED RING-CH FINGER PROTEIN 4; MARCH4




-------------------------------------------
GWAS catalog
-------------------------------------------

rs13015993   Chr 2   217625523 (107973)   IGFBP5      Thyroid hormone levels (TSH)
[Porcu E, 02/07/2013, PLoS Genet]
rs13015993   Chr 2   217625523 (107973)   IGFBP5      Thyroid hormone levels (TSH - Females)
[Porcu E, 02/07/2013, PLoS Genet]
rs13015993   Chr 2   217625523 (107973)   IGFBP5      Thyroid hormone levels (TSH - Males)
[Porcu E, 02/07/2013, PLoS Genet]
rs6435957   Chr 2   217878209 (-144713)   Intergenic  Primary tooth development (number of teeth)
[Pillas D, 02/26/2010, PLoS Genet]
rs4491709   Chr 2   217894756 (-161260)   TNP1        Permanent tooth development
[Geller F, 09/08/2011, PLoS Genet]
rs13387042   Chr 2   217905832 (-172336)  Intergenic  Breast cancer
[Michailidou K, 04/01/2013, Nat Genet]
rs13387042   Chr 2   217905832 (-172336)  Intergenic  Breast cancer
[Fletcher O, 01/24/2011, J Natl Cancer Inst]
rs13387042   Chr 2   217905832 (-172336)  Intergenic  Breast cancer
[Li J, 09/26/2010, Breast Cancer Res Treat]
rs13387042   Chr 2   217905832 (-172336)  Intergenic  Breast cancer
[Turnbull C, 05/09/2010, Nat Genet]
rs13387042   Chr 2   217905832 (-172336)  Intergenic  Breast cancer
[Thomas G, 03/29/2009, Nat Genet]
rs13387042   Chr 2   217905832 (-172336)  Intergenic  Breast cancer
[Stacey SN, 05/27/2007, Nat Genet]
rs2241193   Chr 2   217554213 (179283)    IGFBP5      Visceral fat (men)
```

[Fox CS, 05/10/2012, PLoS Genet]
rs9288520 Chr 2 217481271 (252225) IGFB2 Esophageal cancer (alcohol interaction)
[Wu C, 09/09/2012, Nat Genet]
rs2553026 Chr 2 218123648 (-390152) Intergenic Height
[N'Diaye A, 10/06/2011, PLoS Genet]

Mapping SNP rs11687765   Chr 2   82325531

Distance to locus: -170331
    Gene: 100420968 (LOC100420968)   Chr 2   82495862-82496804 (-)

GWAS catalog
----

rs12052359   Chr 2   81872922 (452609) AC013262.1 Bilirubin levels
[Chen G, 11/16/2011, Eur J Hum Genet]
rs12615721   Chr 2   81856526 (469005) Intergenic Pulmonary function decline (FEV1 decline in asthmatics) [Imboden M, 03/15/2012, J Allergy Clin Immunol]

Mapping SNP rs2540641   Chr 22   18959684

Distance to locus: 0
    Gene: 26220 (DGCR5)   Chr 22   18958027-18982141 (+)

Distance to locus: -25847
    Gene: 100287576 (LOC100287576)   Chr 22   18985531-18987264 (+)

Distance to locus: 35618
    Gene: 5625 (PRODH)   Chr 22   18900287-18924066 (-)
        Pathway: Arginine and proline metabolism - Homo sapiens (human)   (database: KEGG)
            ReactionKEGG: L-Proline + NAD+ <=> (S)-1-Pyrroline-5-carboxylate + NADH + H+
            ReactionKEGG: L-Proline + 2-Oxoglutarate + Oxygen <=> Hydroxyproline + Succinate + CO2
            ReactionKEGG: D-Proline + Oxygen <=> 1-Pyrroline-2-carboxylate + Hydrogen peroxide
            ReactionKEGG: L-Proline + Acceptor <=> (S)-1-Pyrroline-5-carboxylate + Reduced acceptor
            ReactionKEGG: Peptide + H2O <=> L-Proline + Peptide
            ReactionKEGG: L-Proline + NADP+ <=> (S)-1-Pyrroline-5-carboxylate + NADPH + H+
        Pathway: Metabolic pathways - Homo sapiens (human)   (database: KEGG)
            ReactionKEGG: L-Proline + NAD+ <=> (S)-1-Pyrroline-5-carboxylate + NADH + H+
            ReactionKEGG: L-Proline + 2-Oxoglutarate + Oxygen <=> Hydroxyproline + Succinate + CO2
            ReactionKEGG: D-Proline + Oxygen <=> 1-Pyrroline-2-carboxylate + Hydrogen peroxide
            ReactionKEGG: L-Proline + Acceptor <=> (S)-1-Pyrroline-5-carboxylate + Reduced acceptor
            ReactionKEGG: Peptide + H2O <=> L-Proline + Peptide
            ReactionKEGG: ATP + L-Proline + tRNA(Pro) <=> AMP + Diphosphate + L-Prolyl-tRNA(Pro)
            ReactionKEGG: L-Proline + NADP+ <=> (S)-1-Pyrroline-5-carboxylate + NADPH + H+
        Pathway: Prolidase Deficiency(PD)   (database: SMPDB)
            ReactionKEGG: L-Proline + NAD+ <=> (S)-1-Pyrroline-5-carboxylate + NADH + H+
            ReactionKEGG: L-Proline + 2-Oxoglutarate + Oxygen <=> Hydroxyproline + Succinate + CO2
            ReactionKEGG: D-Proline + Oxygen <=> 1-Pyrroline-2-carboxylate + Hydrogen peroxide
            ReactionKEGG: L-Proline + Acceptor <=> (S)-1-Pyrroline-5-carboxylate + Reduced acceptor
            ReactionKEGG: Peptide + H2O <=> L-Proline + Peptide
            ReactionKEGG: ATP + L-Proline + tRNA(Pro) <=> AMP + Diphosphate + L-Prolyl-tRNA(Pro)
            ReactionKEGG: L-Proline + NADP+ <=> (S)-1-Pyrroline-5-carboxylate + NADPH + H+
        Pathway: Arginine and Proline Metabolism   (database: SMPDB)
            ReactionKEGG: L-Proline + NAD+ <=> (S)-1-Pyrroline-5-carboxylate + NADH + H+
            ReactionKEGG: L-Proline + 2-Oxoglutarate + Oxygen <=> Hydroxyproline + Succinate + CO2
            ReactionKEGG: D-Proline + Oxygen <=> 1-Pyrroline-2-carboxylate + Hydrogen peroxide
            ReactionKEGG: L-Proline + Acceptor <=> (S)-1-Pyrroline-5-carboxylate + Reduced acceptor
            ReactionKEGG: Peptide + H2O <=> L-Proline + Peptide
            ReactionKEGG: ATP + L-Proline + tRNA(Pro) <=> AMP + Diphosphate + L-Prolyl-tRNA(Pro)
            ReactionKEGG: L-Proline + NADP+ <=> (S)-1-Pyrroline-5-carboxylate + NADPH + H+
        Pathway: Hyperprolinemia Type I   (database: SMPDB)
            ReactionKEGG: L-Proline + Acceptor <=> (S)-1-Pyrroline-5-carboxylate + Reduced acceptor
        Pathway: Metabolism of amino acids and derivatives   (database: Wikipathways)
            ReactionKEGG: L-Proline + Acceptor <=> (S)-1-Pyrroline-5-carboxylate + Reduced acceptor
        Pathway: Urea cycle and metabolism of arginine, proline, glutamate, aspartate and asparagine   (database: EHMN)
            ReactionKEGG: L-Proline + NAD+ <=> (S)-1-Pyrroline-5-carboxylate + NADH + H+
            ReactionKEGG: L-Proline + 2-Oxoglutarate + Oxygen <=> Hydroxyproline + Succinate + CO2
            ReactionKEGG: D-Proline + Oxygen <=> 1-Pyrroline-2-carboxylate + Hydrogen peroxide
            ReactionKEGG: L-Proline + Acceptor <=> (S)-1-Pyrroline-5-carboxylate + Reduced acceptor

ReactionKEGG: ATP + L-Proline + tRNA(Pro) <=> AMP + Diphosphate + L-Prolyl-tRNA(Pro)
ReactionKEGG: L-Proline + NADP+ <=> (S)-1-Pyrroline-5-carboxylate + NADPH + H+
Pathway: Metabolism of amino acids and derivatives (database: Reactome)
ReactionKEGG: L-Proline + NAD+ <=> (S)-1-Pyrroline-5-carboxylate + NADH + H+
ReactionKEGG: D-Proline + Oxygen <=> 1-Pyrroline-2-carboxylate + Hydrogen peroxide
ReactionKEGG: L-Proline + Acceptor <=> (S)-1-Pyrroline-5-carboxylate + Reduced acceptor
ReactionKEGG: L-Proline + NADP+ <=> (S)-1-Pyrroline-5-carboxylate + NADPH + H+
Pathway: Metabolism (database: Reactome)
ReactionKEGG: L-Proline + NAD+ <=> (S)-1-Pyrroline-5-carboxylate + NADH + H+
ReactionKEGG: D-Proline + Oxygen <=> 1-Pyrroline-2-carboxylate + Hydrogen peroxide
ReactionKEGG: L-Proline + Acceptor <=> (S)-1-Pyrroline-5-carboxylate + Reduced acceptor
ReactionKEGG: L-Proline + NADP+ <=> (S)-1-Pyrroline-5-carboxylate + NADPH + H+
Pathway: Proline catabolism (database: PID)
ReactionKEGG: L-Proline + Acceptor <=> (S)-1-Pyrroline-5-carboxylate + Reduced acceptor
Pathway: citrulline biosynthesis (database: HumanCyc)
ReactionKEGG: L-Proline + Acceptor <=> (S)-1-Pyrroline-5-carboxylate + Reduced acceptor
Pathway: proline degradation (database: HumanCyc)
ReactionKEGG: L-Proline + Acceptor <=> (S)-1-Pyrroline-5-carboxylate + Reduced acceptor
Pathway: superpathway of citrulline metabolism (database: HumanCyc)
ReactionKEGG: L-Proline + Acceptor <=> (S)-1-Pyrroline-5-carboxylate + Reduced acceptor
Pathway: Proline catabolism (database: Reactome)
ReactionKEGG: L-Proline + Acceptor <=> (S)-1-Pyrroline-5-carboxylate + Reduced acceptor
Protein: PROD Proline dehydrogenase 1, mitochondrial;
KO: K00318 proline dehydrogenase [EC:1.5.99.8]
ReactionKEGG: L-Proline + Acceptor <=> (S)-1-Pyrroline-5-carboxylate + Reduced acceptor
ReactionKEGG: L-erythro-4-Hydroxyglutamate + NADH + H+ <=> L-4-Hydroxyglutamate semialdehyde + NAD+ + H2O
R05051+R05052+R01252: NADH + L-Proline + Oxygen + H + D-4-Hydroxy-2-oxoglutarate + L-Glutamate <=> Succinate + Hydroxyproline + NAD + H2O + CO2 + L-4-Hydroxyglutamatesemialdehyde
EC/TCDB: EC:1.5.99.8 Proline dehydrogenase.
ReactionHepatoNet1: NAD_cyto + Proline_cyto <=> NADH_cyto + 1-Pyrroline-5-carboxylate_cyto
ReactionHepatoNet1: NADP_cyto + Proline_cyto <=> NADPH_cyto + 1-Pyrroline-5-carboxylate_cyto
ReactionHepatoNet1: Proline_mito + Ubiquinone_mito <=> Ubiquinol_mito + 1-Pyrroline-5-carboxylate_mito
OMIM: *606810 PROLINE DEHYDROGENASE; PRODH
OMIM: #239500 HYPERPROLINEMIA, TYPE I; HPI
OMIM: #600850 SCHIZOPHRENIA 4; SCZD4

Distance to locus: -42180
Gene: 100506454 (LOC100506454) Chr 22 19001864-19018742 (+)

Distance to locus: -45663
Gene: 25787 (DGCR9) Chr 22 19005347-19007761 (+)

Distance to locus: -50453
Gene: 26222 (DGCR10) Chr 22 19010137-19011063 (+)

Distance to locus: 60083
Gene: 8214 (DGCR6) Chr 22 18893736-18899601 (+)
Protein: DGCR6 Protein DGCR6;
OMIM: *601279 DIGEORGE SYNDROME CRITICAL REGION GENE 6; DGCR6

Distance to locus: -64111
Gene: 9993 (DGCR2) Chr 22 19023795-19109967 (-)
Protein: IDD Integral membrane protein DGCR2/IDD;
OMIM: *600594 DIGEORGE SYNDROME CRITICAL REGION GENE 2; DGCR2

Distance to locus: -73991

Gene: 25786 (DGCR11)   Chr 22   19033675-19035888 (-)

Distance to locus: -83670
Gene: 100129262 (LOC100129262)   Chr 22   19043354-19043700 (+)

Distance to locus: 110386
Gene: 100287541 (LOC100287541)   Chr 22   18842618-18849298 (+)

Distance to locus: 113531
Gene: 100133163 (BCRP7)   Chr 22   18842676-18846153 (-)

Distance to locus: 120362
Gene: 727983 (LOC727983)   Chr 22   18833962-18839322 (+)

Distance to locus: 127240
Gene: 100132900 (LOC100132900)   Chr 22   18781949-18832444 (-)

Distance to locus: 136448
Gene: 386610 (LOC386610)   Chr 22   18821109-18823236 (+)

Distance to locus: -152747
Gene: 23752 (TSSK1A)   Chr 22   19112431-19113380 (+)

Distance to locus: -158108
Gene: 8220 (DGCR14)   Chr 22   19117792-19132190 (-)
Protein: DGC14   Protein DGCR14;
KO: K13118   protein DGCR14
OMIM: *601755 DIGEORGE SYNDROME CRITICAL REGION GENE 14; DGCR14

Distance to locus: -158637
Gene: 23617 (TSSK2)   Chr 22   19118321-19120136 (+)
Protein: TSSK2   Testis-specific serine/threonine-protein kinase 2;
KO: K08811   testis-specific serine kinase [EC:2.7.11.1]
EC/TCDB: EC:2.7.11.1   Non-specific serine/threonine protein kinase.
OMIM: *610710 TESTIS-SPECIFIC SERINE/THREONINE KINASE 2; TSSK2

Distance to locus: -176820
Gene: 2928 (GSC2)   Chr 22   19136504-19137796 (-)
Protein: GSC2   Homeobox protein goosecoid-2;
KO: K09325   homeobox protein goosecoid-like
OMIM: *601845 GOOSECOID HOMEOBOX 2; GSC2

Distance to locus: 180210
Gene: 2679 (GGT3P)   Chr 22   18761202-18779474 (-)
Pathway: Glutathione synthesis and recycling   (database: Reactome)
Pathway: Biological oxidations   (database: Reactome)
Pathway: Metabolism   (database: Reactome)
ReactionKEGG: L-Proline + NAD+ <=> (S)-1-Pyrroline-5-carboxylate + NADH + H+
ReactionKEGG: D-Proline + Oxygen <=> 1-Pyrroline-2-carboxylate + Hydrogen peroxide
ReactionKEGG: L-Proline + Acceptor <=> (S)-1-Pyrroline-5-carboxylate + Reduced acceptor
ReactionKEGG: L-Proline + NADP+ <=> (S)-1-Pyrroline-5-carboxylate + NADPH + H+
Pathway: Glutathione conjugation   (database: Reactome)
Pathway: Phase II conjugation   (database: Reactome)
Pathway: γ-glutamyl cycle   (database: HumanCyc)

Distance to locus: -199224
Gene: 100652736 (LOC100652736)   Chr 22   19158908-19160342 (+)

Distance to locus: -203410
Gene: 6576 (SLC25A1)   Chr 22   19163094-19166301 (-)
Pathway: Fatty acid, triacylglycerol, and ketone body metabolism   (database: Wikipathways)
Pathway: Metabolism of carbohydrates   (database: Wikipathways)
Pathway: Epithelium TarBase   (database: Wikipathways)
ReactionKEGG: L-Proline + 2-Oxoglutarate + Oxygen <=> Hydroxyproline +

Succinate + CO2
Pathway: Leukocyte TarBase (database: Wikipathways)
Pathway: Lymphocyte TarBase (database: Wikipathways)
ReactionKEGG: L-Proline + 2-Oxoglutarate + Oxygen <=> Hydroxyproline + Succinate + CO2
Pathway: Muscle cell TarBase (database: Wikipathways)
ReactionKEGG: L-Proline + 2-Oxoglutarate + Oxygen <=> Hydroxyproline + Succinate + CO2
Pathway: Glycolysis and Gluconeogenesis (database: EHMN)
Pathway: TCA cycle (database: EHMN)
Pathway: Metabolism of carbohydrates (database: Reactome)
Pathway: Metabolism (database: Reactome)
ReactionKEGG: L-Proline + NAD+ <=> (S)-1-Pyrroline-5-carboxylate + NADH + H+
ReactionKEGG: D-Proline + Oxygen <=> 1-Pyrroline-2-carboxylate + Hydrogen peroxide
ReactionKEGG: L-Proline + Acceptor <=> (S)-1-Pyrroline-5-carboxylate + Reduced acceptor
ReactionKEGG: L-Proline + NADP+ <=> (S)-1-Pyrroline-5-carboxylate + NADPH + H+
Pathway: Glucose metabolism (database: Reactome)
Pathway: Fatty Acyl-CoA Biosynthesis (database: Reactome)
Pathway: Triglyceride Biosynthesis (database: Reactome)
Pathway: Fatty acid, triacylglycerol, and ketone body metabolism (database: Reactome)
Pathway: Metabolism of lipids and lipoproteins (database: Reactome)
Pathway: Fatty Acyl-CoA Biosynthesis (database: PID)
Pathway: Gluconeogenesis (database: PID)
Pathway: Gluconeogenesis (database: Reactome)
Protein: TXTP Tricarboxylate transport protein, mitochondrial;
KO: K15100 solute carrier family 25 (mitochondrial citrate transporter), member 1
EC/TCDB: TCDB:2.A.29.7.2 mitochondrial carrier (MC) family.
ReactionHepatoNet1: Malate_mito + Citrate_cyto <=> Malate_cyto + Citrate_mito
ReactionHepatoNet1: Malate_cyto + Isocitrate_mito <=> Malate_mito + Isocitrate_cyto
ReactionHepatoNet1: Succinate_mito + Citrate_cyto <=> Succinate_cyto + Citrate_mito
ReactionHepatoNet1: PEP_mito + Citrate_cyto <=> PEP_cyto + Citrate_mito
ReactionHepatoNet1: Citrate_cyto + Isocitrate_mito <=> Citrate_mito + Isocitrate_cyto
ReactionHepatoNet1: Citrate_blood + Na_blood --> Citrate_cyto + Na_cyto
ReactionHepatoNet1: AKG_cyto + Isocitrate_mito <=> AKG_mito + Isocitrate_cyto
ReactionHepatoNet1: Citrate_mito + cis-Aconitate_cyto <=> Citrate_cyto + cis-Aconitate_mito
ReactionHepatoNet1: Isocitrate_mito + cis-Aconitate_cyto <=> Isocitrate_cyto + cis-Aconitate_mito
ReactionHepatoNet1: AKG_cyto + Citrate_mito <=> AKG_mito + Citrate_cyto
ReactionHepatoNet1: Citrate_mito + Oxalate_cyto <=> Citrate_cyto + Oxalate_mito
ReactionHepatoNet1: Succinate_cyto + Isocitrate_mito <=> Succinate_mito + Isocitrate_cyto
ReactionHepatoNet1: Oxalate_cyto + Isocitrate_mito <=> Oxalate_mito + Isocitrate_cyto
ReactionHepatoNet1: AKG_cyto + cis-Aconitate_mito <=> AKG_mito + cis-Aconitate_cyto
ReactionHepatoNet1: Succinate_cyto + cis-Aconitate_mito <=> Succinate_mito + cis-Aconitate_cyto
ReactionHepatoNet1: Malate_cyto + cis-Aconitate_mito <=> Malate_mito + cis-Aconitate_cyto
ReactionHepatoNet1: Oxalate_cyto + cis-Aconitate_mito <=> Oxalate_mito + cis-Aconitate_cyto
ReactionHepatoNet1: AKG_cyto + H(PG)_mito + Citrate_mito <-- AKG_mito + H(PG)_cyto + Citrate_cyto
ReactionHepatoNet1: Succinate_cyto + H(PG)_mito + Citrate_mito <-- Succinate_mito + H(PG)_cyto + Citrate_cyto
ReactionHepatoNet1: H(PG)_mito + Malate_cyto + Citrate_mito <-- H(PG)_cyto + Malate_mito + Citrate_cyto
ReactionHepatoNet1: H(PG)_mito + Citrate_mito + Oxalate_cyto <-- H(PG)_cyto + Citrate_cyto + Oxalate_mito
ReactionHepatoNet1: AKG_cyto + H(PG)_mito + Isocitrate_mito <-- AKG_mito + H(PG)_cyto + Isocitrate_cyto
ReactionHepatoNet1: Succinate_cyto + H(PG)_mito + Isocitrate_mito <-- Succinate_mito + H(PG)_cyto + Isocitrate_cyto
ReactionHepatoNet1: H(PG)_mito + Malate_cyto + Isocitrate_mito <-- H(PG)_cyto + Malate_mito + Isocitrate_cyto
ReactionHepatoNet1: H(PG)_mito + Oxalate_cyto + Isocitrate_mito <-- H(PG)_cyto + Oxalate_mito + Isocitrate_cyto
ReactionHepatoNet1: AKG_cyto + H(PG)_mito + cis-Aconitate_mito <--

AKG_mito + H(PG)_cyto + cis-Aconitate_cyto
ReactionHepatoNet1: Succinate_cyto + H(PG)_mito + cis-Aconitate_mito <-- Succinate_mito + H(PG)_cyto + cis-Aconitate_cyto
ReactionHepatoNet1: H(PG)_mito + Malate_cyto + cis-Aconitate_mito <-- H(PG)_cyto + Malate_mito + cis-Aconitate_cyto
ReactionHepatoNet1: H(PG)_mito + Oxalate_cyto + cis-Aconitate_mito <-- H(PG)_cyto + Oxalate_mito + cis-Aconitate_cyto
ReactionHepatoNet1: PEP_cyto + Isocitrate_mito <=> PEP_mito + Isocitrate_cyto
ReactionHepatoNet1: PEP_cyto + cis-Aconitate_mito <=> PEP_mito + cis-Aconitate_cyto
OMIM: *190315 SOLUTE CARRIER FAMILY 25 (MITOCHONDRIAL CARRIER, CITRATE TRANSPORTER),MEMBER 1; SLC25A1
OMIM: #615182 COMBINED D-2- AND L-2-HYDROXYGLUTARIC ACIDURIA; D2L2AD

Distance to locus: -207302
Gene: 8218 (CLTCL1) Chr 22 19166986-19279239 (-)
Pathway: Synaptic vesicle cycle - Homo sapiens (human) (database: KEGG)
Pathway: Endocytosis - Homo sapiens (human) (database: KEGG)
Pathway: Lysosome - Homo sapiens (human) (database: KEGG)
Pathway: Huntington,s disease - Homo sapiens (human) (database: KEGG)
Pathway: Endocrine and other factor-regulated calcium reabsorption - Homo sapiens (human) (database: KEGG)
Pathway: Bacterial invasion of epithelial cells - Homo sapiens (human) (database: KEGG)
Pathway: Synaptic Vesicle Pathway (database: Wikipathways)
Pathway: Formation of annular gap junctions (database: Reactome)
Pathway: Gap junction degradation (database: Reactome)
Pathway: Gap junction trafficking (database: Reactome)
Pathway: Membrane Trafficking (database: Reactome)
Pathway: Gap junction trafficking and regulation (database: Reactome)
Protein: CLH2 Clathrin heavy chain 2;
KO: K04646 clathrin heavy chain
OMIM: *601273 CLATHRIN, HEAVY POLYPEPTIDE-LIKE 1; CLTCL1

Distance to locus: 221843
Gene: 642643 (LOC642643) Chr 22 18721540-18737841 (+)

Distance to locus: 252726
Gene: 100132295 (KIAA0649P4) Chr 22 18703328-18706958 (+)

Distance to locus: -266282
Gene: 100271504 (RPL34P35) Chr 22 19225966-19226262 (-)

Distance to locus: 274480
Gene: 100652750 (LOC100652750) Chr 22 18666950-18685204 (-)

Distance to locus: -280960
Gene: 8215 (DVL1L1) Chr 22 19240644-19240992 (-)
Pathway: Wnt Canonical (database: INOH)
Pathway: Wnt Mammals (database: INOH)
OMIM: *601225 DISHEVELLED 1-LIKE 1; DVL1L1

Distance to locus: -285316
Gene: 100287635 (LOC100287635) Chr 22 19245000-19246384 (+)

Distance to locus: 299520
Gene: 11274 (USP18) Chr 22 18632758-18660164 (+)
Pathway: Interferon alpha-beta signaling (database: Wikipathways)
Pathway: Cytokine Signaling in Immune system (database: Reactome)
Pathway: Immune System (database: Reactome)
Pathway: Regulation of IFNA signaling (database: Reactome)
Pathway: Interferon alpha/beta signaling (database: Reactome)
Pathway: Interferon Signaling (database: Reactome)
Protein: UBP18 Ubl carboxyl-terminal hydrolase 18;
KO: K11846 ubiquitin carboxyl-terminal hydrolase 18/41 [EC:3.1.2.15]
OMIM: *607057 UBIQUITIN-SPECIFIC PROTEASE 18; USP18

Distance to locus: 319065
Gene: 100506285 (LOC100506285) Chr 22 18632622-18640619 (-)

Distance to locus: 345186

Gene: 51807 (TUBA8)   Chr 22   18593453-18614498 (+)
Pathway: Phagosome - Homo sapiens (human)   (database: KEGG)
Pathway: Gap junction - Homo sapiens (human)   (database: KEGG)
Pathway: Pathogenic Escherichia coli infection - Homo sapiens (human)   (database: KEGG)
Pathway: Pathogenic Escherichia coli infection   (database: Wikipathways)
Pathway: stathmin and breast cancer resistance to antimicrotubule agents   (database: PID)
Pathway: downregulated of mta-3 in er-negative breast tumors   (database: PID)
Pathway: stathmin and breast cancer resistance to antimicrotubule agents   (database: BioCarta)
Pathway: downregulated of mta-3 in er-negative breast tumors   (database: BioCarta)
Protein: TBA8   Tubulin alpha-8 chain;
KO: K07374   tubulin alpha
OMIM: *605742 TUBULIN, ALPHA-8; TUBA8
OMIM: #613180 POLYMICROGYRIA WITH OPTIC NERVE HYPOPLASIA

Distance to locus: -358540
Gene: 7290 (HIRA)   Chr 22   19318224-19419219 (-)
Protein: HIRA   Protein HIRA;
KO: K11293   protein HIRA/HIR1
OMIM: *600237 HISTONE CELL CYCLE REGULATION DEFECTIVE, S. CEREVISIAE, HOMOLOG OF,A; HIRA

Distance to locus: 385887
Gene: 55670 (PEX26)   Chr 22   18560686-18573797 (+)
Pathway: Peroxisome - Homo sapiens (human)   (database: KEGG)
ReactionKEGG: D-Proline + Oxygen <=> 1-Pyrroline-2-carboxylate + Hydrogen peroxide
Protein: PEX26   Peroxisome assembly protein 26;
KO: K13340   peroxin-26
EC/TCDB: TCDB:3.A.20.1.1   peroxisomal protein importer (PPI) family.
EC/TCDB: TCDB:3.A.20.1.1   peroxisomal protein importer (PPI) family.
EC/TCDB: TCDB:3.A.20.1.1   peroxisomal protein importer (PPI) family.
EC/TCDB: TCDB:3.A.20.1.1   peroxisomal protein importer (PPI) family.
EC/TCDB: TCDB:3.A.20.1.1   peroxisomal protein importer (PPI) family.
EC/TCDB: TCDB:3.A.20.1.1   peroxisomal protein importer (PPI) family.
EC/TCDB: TCDB:3.A.20.1.1   peroxisomal protein importer (PPI) family.
EC/TCDB: TCDB:3.A.20.1.1   peroxisomal protein importer (PPI) family.
EC/TCDB: TCDB:3.A.20.1.1   peroxisomal protein importer (PPI) family.
EC/TCDB: TCDB:3.A.20.1.1   peroxisomal protein importer (PPI) family.
EC/TCDB: TCDB:3.A.20.1.1   peroxisomal protein importer (PPI) family.
OMIM: *608666 PEROXISOME BIOGENESIS FACTOR 26; PEX26
OMIM: #614872 PEROXISOME BIOGENESIS DISORDER 7A (ZELLWEGER); PBD7A   PEROXISOME BIOGENESIS DISORDER, COMPLEMENTATION GROUP 8, INCLUDED;CG8, INCLUDED
OMIM: #614873 PEROXISOME BIOGENESIS DISORDER 7B; PBD7B

Distance to locus: 438950
Gene: 100192420 (FLJ41941)   Chr 22   18512151-18520734 (+)

Distance to locus: 452359
Gene: 57553 (MICAL3)   Chr 22   18270415-18507325 (-)
Protein: MICA3   Protein-methionine sulfoxide oxidase MICAL3;
OMIM: *608882 MICROTUBULE-ASSOCIATED MONOOXYGENASE, CALPONIN AND LIM DOMAINS-CONTAINING,3; MICAL3

Distance to locus: 456314
Gene: 692231 (LOC692231)   Chr 22   18501972-18503370 (-)

Distance to locus: -460352
Gene: 64976 (MRPL40)   Chr 22   19420036-19423598 (+)
Protein: RM40   39S ribosomal protein L40, mitochondrial;
OMIM: *605089 MITOCHONDRIAL RIBOSOMAL PROTEIN L40; MRPL40

Distance to locus: -468725
Gene: 128977 (C22orf39)   Chr 22   19428409-19435755 (-)
Protein: CV039   UPF0545 protein C22orf39;

Distance to locus: -475732
Gene: 100506503 (LOC100506503)   Chr 22   19435416-19437686 (+)

Distance to locus: -477780
Gene: 7353 (UFD1L)   Chr 22   19437464-19466738 (-)

Pathway: Protein processing in endoplasmic reticulum - Homo sapiens (human) (database: KEGG)
Protein: UFD1 Ubiquitin fusion degradation protein 1 homolog;
KO: K14016 ubiquitin fusion degradation protein 1
OMIM: *601754 UBIQUITIN FUSION DEGRADATION 1-LIKE; UFD1L

Distance to locus: 495957
Gene: 693233 (MIR648) Chr 22 18463634-18463727 (-)

GWAS catalog
----

rs2023634 Chr 22 18972450 (-12766) PRODH Metabolic traits (proline + 5 other traits) [Suhre K, 08/31/2011, Nature]
rs807669 Chr 22 19154522 (-194838) SLC25A1 Metabolite levels (Citrate) [Kettunen J, 01/29/2012, Nat Genet]
rs712964 Chr 22 19156117 (-196433) GSC2, SLC25A1, CLTCL1 Metabolite levels [Inouye M, 08/16/2012, PLoS Genet]

Mapping SNP rs248386 Chr 5 78330227

Distance to locus: 0
Gene: 29958 (DMGDH) Chr 5 78293429-78365449 (-)
Pathway: Glycine, serine and threonine metabolism - Homo sapiens (human) (database: KEGG)
ReactionKEGG: N,N-Dimethylglycine + Electron-transferring flavoprotein + H2O <=> Sarcosine + Formaldehyde + Reduced electron-transferring flavoprotein
ReactionKEGG: Betaine + L-Homocysteine <=> N,N-Dimethylglycine + L-Methionine
Pathway: Metabolic pathways - Homo sapiens (human) (database: KEGG)
ReactionKEGG: N,N-Dimethylglycine + Electron-transferring flavoprotein + H2O <=> Sarcosine + Formaldehyde + Reduced electron-transferring flavoprotein
ReactionKEGG: Betaine + L-Homocysteine <=> N,N-Dimethylglycine + L-Methionine
Pathway: Glycine, Serine and Threonine Metabolism (database: SMPDB)
ReactionKEGG: N,N-Dimethylglycine + Electron-transferring flavoprotein + H2O <=> Sarcosine + Formaldehyde + Reduced electron-transferring flavoprotein
ReactionKEGG: Betaine + L-Homocysteine <=> N,N-Dimethylglycine + L-Methionine
Pathway: Dimethylglycine Dehydrogenase Deficiency (database: SMPDB)
ReactionKEGG: N,N-Dimethylglycine + Electron-transferring flavoprotein + H2O <=> Sarcosine + Formaldehyde + Reduced electron-transferring flavoprotein
Pathway: Dihydropyrimidine Dehydrogenase Deficiency (DHPD) (database: SMPDB)
ReactionKEGG: N,N-Dimethylglycine + Electron-transferring flavoprotein + H2O <=> Sarcosine + Formaldehyde + Reduced electron-transferring flavoprotein
Pathway: Glycine, serine, alanine and threonine metabolism (database: EHMN)
ReactionKEGG: N,N-Dimethylglycine + Electron-transferring flavoprotein + H2O <=> Sarcosine + Formaldehyde + Reduced electron-transferring flavoprotein
Pathway: Glycine Serine metabolism (database: INOH)
ReactionKEGG: N,N-Dimethylglycine + Electron-transferring flavoprotein + H2O <=> Sarcosine + Formaldehyde + Reduced electron-transferring flavoprotein
ReactionKEGG: Betaine + L-Homocysteine <=> N,N-Dimethylglycine + L-Methionine
Pathway: glycine betaine degradation (database: HumanCyc)
ReactionKEGG: N,N-Dimethylglycine + Electron-transferring flavoprotein + H2O <=> Sarcosine + Formaldehyde + Reduced electron-transferring flavoprotein
ReactionKEGG: Betaine + L-Homocysteine <=> N,N-Dimethylglycine + L-Methionine
Protein: M2GD Dimethylglycine dehydrogenase, mitochondrial;
KO: K00315 dimethylglycine dehydrogenase [EC:1.5.8.4]
ReactionKEGG: N,N-Dimethylglycine + Electron-transferring flavoprotein + H2O <=> Sarcosine + Formaldehyde + Reduced electron-transferring flavoprotein
EC/TCDB: EC:1.5.8.4 Dimethylglycine dehydrogenase.
OMIM: *605849 DIMETHYLGLYCINE DEHYDROGENASE; DMGDH
OMIM: #605850 DIMETHYLGLYCINE DEHYDROGENASE DEFICIENCY; DMGDHD

Distance to locus: -35320
Gene: 23743 (BHMT2) Chr 5 78365547-78385897 (+)
Pathway: superpathway of methionine degradation (database: HumanCyc)
ReactionKEGG: Betaine + L-Homocysteine <=> N,N-Dimethylglycine + L-Methionine
Pathway: glycine betaine degradation (database: HumanCyc)
ReactionKEGG: N,N-Dimethylglycine + Electron-transferring flavoprotein + H2O <=> Sarcosine + Formaldehyde + Reduced electron-transferring flavoprotein
ReactionKEGG: Betaine + L-Homocysteine <=> N,N-Dimethylglycine + L-Methionine
Pathway: methionine salvage II (mammalia) (database: HumanCyc)
ReactionKEGG: Betaine + L-Homocysteine <=> N,N-Dimethylglycine + L-Methionine
Protein: BHMT2 S-methylmethionine--homocysteine S-methyltransferase BHMT2;
EC/TCDB: EC:2.1.1.5 Betaine--homocysteine S-methyltransferase.
OMIM: *605932 BETAINE-HOMOCYSTEINE METHYLTRANSFERASE 2; BHMT2

Distance to locus: 47870
Gene: 411 (ARSB) Chr 5 78073032-78282357 (-)
Pathway: Lysosome - Homo sapiens (human) (database: KEGG)
Pathway: Metabolic pathways - Homo sapiens (human) (database: KEGG)
ReactionKEGG: N,N-Dimethylglycine + Electron-transferring flavoprotein + H2O <=> Sarcosine + Formaldehyde + Reduced electron-transferring flavoprotein
ReactionKEGG: Betaine + L-Homocysteine <=> N,N-Dimethylglycine + L-Methionine
Pathway: Glycosaminoglycan degradation - Homo sapiens (human) (database: KEGG)
Pathway: Androgen and estrogen biosynthesis and metabolism (database: EHMN)
Pathway: PTM: gamma carboxylation, hypusine formation and arylsulfatase activation (database: Reactome)
Pathway: Disease (database: Reactome)
Pathway: Metabolism of carbohydrates (database: Reactome)
Pathway: Metabolism (database: Reactome)
ReactionKEGG: Betaine + L-Homocysteine <=> N,N-Dimethylglycine + L-Methionine
Pathway: Post-translational protein modification (database: Reactome)

Pathway: Metabolism of proteins (database: Reactome)
Pathway: Phospholipid metabolism (database: Reactome)
Pathway: CS/DS degradation (database: Reactome)
Pathway: Chondroitin sulfate/dermatan sulfate metabolism (database: Reactome)
Pathway: Glycosaminoglycan metabolism (database: Reactome)
Pathway: MPS VII - Sly syndrome (database: Reactome)
Pathway: MPS IIIA - Sanfilippo syndrome A (database: Reactome)
Pathway: MPS IX - Natowicz syndrome (database: Reactome)
Pathway: MPS I - Hurler syndrome (database: Reactome)
Pathway: MPS II - Hunter syndrome (database: Reactome)
Pathway: MPS IIIB - Sanfilippo syndrome B (database: Reactome)
Pathway: MPS IIID - Sanfilippo syndrome D (database: Reactome)
Pathway: MPS IV - Morquio syndrome A (database: Reactome)
Pathway: MPS IV - Morquio syndrome B (database: Reactome)
Pathway: MPS VI - Maroteaux-Lamy syndrome (database: Reactome)
Pathway: Mucopolysaccharidoses (database: Reactome)
Pathway: chondroitin sulfate degradation (metazoa) (database: HumanCyc)
Pathway: Metabolism of lipids and lipoproteins (database: Reactome)
Pathway: MPS IIIC - Sanfilippo syndrome C (database: Reactome)
Pathway: The activation of arylsulfatases (database: Reactome)
Pathway: Glycosphingolipid metabolism (database: Reactome)
Pathway: Sphingolipid metabolism (database: Reactome)
Protein: ARSB Arylsulfatase B;
KO: K01135 arylsulfatase B [EC:3.1.6.12]
ReactionKEGG: G12336 + H2O <=> G13043 + Sulfate
EC/TCDB: EC:3.1.6.12 N-acetylgalactosamine-4-sulfatase.
OMIM: *611542 ARYLSULFATASE B; ARSB
OMIM: #253200 MUCOPOLYSACCHARIDOSIS TYPE VI

Distance to locus: -77377
Gene: 635 (BHMT) Chr 5 78407604-78428113 (+)
Pathway: Cysteine and methionine metabolism - Homo sapiens (human) (database: KEGG)
ReactionKEGG: Betaine + L-Homocysteine <=> N,N-Dimethylglycine + L-Methionine
Pathway: Glycine, serine and threonine metabolism - Homo sapiens (human) (database: KEGG)
ReactionKEGG: N,N-Dimethylglycine + Electron-transferring flavoprotein + H2O <=> Sarcosine + Formaldehyde + Reduced electron-transferring flavoprotein
ReactionKEGG: Betaine + L-Homocysteine <=> N,N-Dimethylglycine + L-Methionine
Pathway: Metabolic pathways - Homo sapiens (human) (database: KEGG)
ReactionKEGG: N,N-Dimethylglycine + Electron-transferring flavoprotein + H2O <=> Sarcosine + Formaldehyde + Reduced electron-transferring flavoprotein
ReactionKEGG: Betaine + L-Homocysteine <=> N,N-Dimethylglycine + L-Methionine
Pathway: Methionine Metabolism (database: SMPDB)
ReactionKEGG: Betaine + L-Homocysteine <=> N,N-Dimethylglycine + L-Methionine
Pathway: Glycine, Serine and Threonine Metabolism (database: SMPDB)
ReactionKEGG: N,N-Dimethylglycine + Electron-transferring flavoprotein + H2O <=> Sarcosine + Formaldehyde + Reduced electron-transferring flavoprotein
ReactionKEGG: Betaine + L-Homocysteine <=> N,N-Dimethylglycine + L-Methionine
Pathway: Betaine Metabolism (database: SMPDB)
ReactionKEGG: Betaine + L-Homocysteine <=> N,N-Dimethylglycine + L-Methionine
Pathway: One Carbon Metabolism (database: Wikipathways)
ReactionKEGG: Betaine + L-Homocysteine <=> N,N-Dimethylglycine + L-Methionine
Pathway: superpathway of methionine degradation (database: HumanCyc)
ReactionKEGG: Betaine + L-Homocysteine <=> N,N-Dimethylglycine + L-Methionine
Pathway: Metabolism of amino acids and derivatives (database: Reactome)
ReactionKEGG: Betaine + L-Homocysteine <=> N,N-Dimethylglycine + L-Methionine
Pathway: Metabolism (database: Reactome)
ReactionKEGG: Betaine + L-Homocysteine <=> N,N-Dimethylglycine + L-Methionine
Pathway: Sulfur amino acid metabolism (database: Reactome)
ReactionKEGG: Betaine + L-Homocysteine <=> N,N-Dimethylglycine + L-Methionine
Pathway: Glycine Serine metabolism (database: INOH)
ReactionKEGG: N,N-Dimethylglycine + Electron-transferring flavoprotein + H2O <=> Sarcosine + Formaldehyde + Reduced electron-transferring flavoprotein
ReactionKEGG: Betaine + L-Homocysteine <=> N,N-Dimethylglycine + L-Methionine
Pathway: glycine betaine degradation (database: HumanCyc)
ReactionKEGG: N,N-Dimethylglycine + Electron-transferring flavoprotein + H2O <=> Sarcosine + Formaldehyde + Reduced electron-transferring flavoprotein
ReactionKEGG: Betaine + L-Homocysteine <=> N,N-Dimethylglycine + L-Methionine
Pathway: Methionine Cysteine metabolism (database: INOH)
ReactionKEGG: Betaine + L-Homocysteine <=> N,N-Dimethylglycine + L-Methionine
Pathway: methionine salvage II (mammalia) (database: HumanCyc)
ReactionKEGG: Betaine + L-Homocysteine <=> N,N-Dimethylglycine + L-Methionine
Protein: BHMT1 Betaine--homocysteine S-methyltransferase 1;
KO: K00544 betaine-homocysteine S-methyltransferase [EC:2.1.1.5]
ReactionKEGG: Betaine + L-Homocysteine <=> N,N-Dimethylglycine + L-Methionine
EC/TCDB: EC:2.1.1.5 Betaine--homocysteine S-methyltransferase.

```
        OMIM: *602888 BETAINE-HOMOCYSTEINE METHYLTRANSFERASE; BHMT


Distance to locus: -201698
    Gene: 133746 (JMY)   Chr 5   78531925-78623038  (+)
        Pathway: Direct p53 effectors   (database: PID)
        Protein: JMY   Junction-mediating and -regulatory protein;
        OMIM: *604279 JUNCTION-MEDIATING AND REGULATORY PROTEIN; JMY


Distance to locus: -249684
    Gene: 643932 (RPS3AP20)   Chr 5   78579911-78580769  (-)


Distance to locus: -339559
    Gene: 9456 (HOMER1)   Chr 5   78669786-78809700  (-)
        Pathway: Glutamatergic synapse - Homo sapiens (human)   (database: KEGG)
        Protein: HOME1   Homer protein homolog 1;
            KO: K15010   homer
        OMIM: *604798 HOMER, DROSOPHILA, HOMOLOG OF, 1; HOMER1


Distance to locus: 385579
    Gene: 10184 (LHFPL2)   Chr 5   77781038-77944648  (-)
        Protein: LHPL2   Lipoma HMGIC fusion partner-like 2 protein;
        OMIM: *609718 LHFP-LIKE PROTEIN 2; LHFPL2


Distance to locus: 389559
    Gene: 100419947 (LOC100419947)   Chr 5   77940065-77940668  (-)


Distance to locus: -475978
    Gene: 728820 (RPL29P15)   Chr 5   78806205-78806840  (+)


Distance to locus: -494877
    Gene: 133748 (RPL7AP32)   Chr 5   78825104-78825974  (+)




-------------------------------------------
GWAS catalog
-------------------------------------------

rs17823642   Chr 5   78341297 (-11070)   BHMT, BHMT2  Metabolite levels (betaine)
[Xie W, 02/01/2013, Diabetes]
rs2052550   Chr 5   78272942 (57285)     ARSB         Iron status biomarkers (serum ferritin)
[Benyamin B, 12/17/2008, Am J Hum Genet]
rs337847   Chr 5   78259888 (70339)      ARSB         Hippocampal atrophy
[Potkin SG, 08/07/2009, PLoS One]
rs784420   Chr 5   77987524 (342703)     Intergenic   Economic and political preferences (Trust)
[Benjamin DJ, 05/07/2012, Proc Natl Acad Sci U S A]
rs10078095   Chr 5   78756778 (-426551)  HOMER1       Height
[Sanna S, 01/13/2008, Nat Genet]
rs7713917   Chr 5   78829249 (-499022)   HOMER1       Major depressive disorder
[Rietschel M, 07/29/2010, Biol Psychiatry]
```
