# Supplementary material for: Insight in Genome-Wide Association of Metabolite Quantitative Traits by Exome Sequence Analyses
Source: PLoS Genet. 2015 Jan 8;11(1):e1004835. doi: 10.1371/journal.pgen.1004835 (PMC4287344; doi:10.1371/journal.pgen.1004835)
Supplement: S2 Text — Methods on NMR spectroscopy, genotyping, exome sequencing and statistics. (PDF) [file pgen.1004835.s004.pdf]

Supplementary Text 2

Methods on NMR spectroscopy, genotyping and statistics

NMR Sample Preparation

The serum samples were thawed at 4°C and were mixed by inverting the containers 10 times. The samples (300 µL) were mixed with 300 µL 75 mM disodium phosphate buffer in H_2O/D_2O (80/20) with a pH of 7.4 containing 6.15 mM NaN_3 and 4.64 mM sodium 3-[trimethylsilyl] d4-propionate (TSP) using a Gilson 215 liquid handler in combination with a Bruker SampleTrack system. Samples were transferred into 5 mm SampleJet NMR tubes in 96 tube racks using a modified Gilson 215 tube filling station and kept at 6°C on a SampleJet sample changer while queued for acquisition.

Acquisition and processing of the NMR spectra

All proton nuclear magnetic resonance (^{1}H-NMR) experiments were acquired on a 600 MHz Bruker Avance II spectrometer (Bruker BioSpin, Karlsruhe, Germany) equipped with a 5mm TCI cryogenic probe head with Z-gradient system and automatic tuning and matching. The NMR pulse sequences used for this study were the CPMG (Carr-Purcell-Meiboom-Gill) and 2D J-resolved (JRES) experiments. All experiments were performed at 310 K. Temperature calibration was done prior to each batch of measurements using the method of Findeisen *et al.* [*1*]. Duration of the π/2 pulses was automatically calibrated for each individual sample using a homonuclear-gated nutation experiment on the locked and shimmed samples after automatic tuning and matching of the probe head [1].

For water suppression, presaturation of the water resonance with an effective field of γB_1 = 25 Hz was applied during the relaxation delay [2]. J-resolved spectra ((JRES) were recorded with a relaxation delay of 2 s and a single scan for each increment in the indirect dimension. A data matrix of 40 x 12,288 data points was collected covering a sweep width of 78 x 10,000 Hz. A sine-shaped window function was applied and the data was zero-filled to 256 x 16,384 complex data points prior

to Fourier transformation. In order to remove the skew, the resulting data matrix was tilted along the rows by shifting each row (k) by 0.4992*(128-k) points and symmetrised about the central horizontal lines. For T2-filtered ^{1}H-NMR spectra, a standard 1D CPMG (Carr–Purcell–Meiboom–Gill) pulse sequence [3] was used with a relaxation delay of 4 seconds. A pulse train of 130 refocusing pulses with individual spin echo delays of 0.6 ms were applied, resulting in a total T2 filtering delay of 78 ms. 73,728 data points covering a spectral width of 12,019 Hz were collected using 16 scans. The FID was zero-filled to 131,072 complex data points and an exponential window function was applied with a line broadening factor of 1.0 Hz prior to Fourier transformation. The spectra were automatically phase and baseline corrected.

The spectra were imported into Matlab® (R2009a, The Mathworks Inc., Natick, MA, USA) and processed further using in-house code. Within Matlab, the CPMG spectra were baseline-corrected by subtracting the mean value of empty regions of the spectrum upfield from -3 ppm and downfield from 12.5 ppm. Next, the spectra referenced to the anomeric glucose resonance (5.23 ppm) because this peak shows less positional variation than the TSP peak. Piecewise cubic spline interpolation was used to exactly align the points of the different spectra. Binning was applied with a bin size of 0.002 ppm. Metabolite intensities were determined by applying metabolite-specific linear models to optimal regions in the binned CPMG spectra. For the metabolites that are represented by more than one peak, the ones with the highest intensity in spectral areas with the least spectral crowding were selected (Supplementary Table 2).

Heritability estimation

Heritability estimations were obtained using SOLAR software [4] (version 6.6.2, http://www.txbiomed.org/departments/genetics/) . Since SOLAR is sensitive for distortion of traits distribution we transformed the metabolite and ratio traits such that the resulting data followed a normal

distribution. For this reason the data were rank transformed. Subsequently SOLAR's polygenic model with age and gender as covariates was applied. Heritability estimations included a second variance component, the sibship effect (S), which is an estimate of phenotypic similarity, due to effects of a shared (early) environment and genetically dominant effects.

Genome-wide association analysis

Samples showing trait value below or above the mean +/- 4*standard deviations from the mean were removed. All genetic analysis were performed using R 2.15.3 and the statistical package GenABEL v. 1.7-4 [5] applying a polygenic model and adjustment for gender age and kinship. Genotypes were based on 300K Beadchip of Illumina and imputed using MACH 1.0 using the HapMap 22 build 36. SNPs with call rate < 0.98, MAF < 0.1 or HWE P-value < 1×10^{-6} were excluded prior to imputation.

Exome sequencing

Rare variant analysis were performed with the third data freeze (N = 1309) from the ERF pedigree which were sequenced "in-house" at the Center for Biomics of the Cell Biology department of the Erasmus MC, The Netherlands, using the Agilent version V4 capture kit on an Illumina Hiseq2000 sequencer using the TruSeq Version 3 protocol. The sequence reads were aligned to the human genome build 19 (hg19) using BWA and the NARWHAL pipeline[6,7]. Subsequently, the aligned reads were processed further using the IndelRealigner, MarkDuplicates and TableRecalibration tools from the Genome Analysis Toolkit (GATK) [8] and Picard (http://broadinstitute.github.io/picard/) to remove systematic biases and to recalibrate the PHRED quality scores in the alignments. After processing, genetic variants were called using the Unified Genotyper tool from the GATK. The effects of the called variants on the respective protein sequences were determined with a custom variant annotation script. For each sample, at least 4 Gigabases of sequence was aligned to the genome.

Further for comparison and prediction of the functionality of the variants annotations were also performed using the dbNSFP (database of human non-synonymous SNPs and their functional predictions) and Seattle databases available at: http://snp.gs.washington.edu/SeattleSeqAnnotation141/. These databases gave functional prediction results from four different programs including polyPhen2, SIFT, MutationTaster and LRT, apart from gene and variant annotations. In total about 1.2 million Single Nucleotide Variants (SNVs) were called. After removing the low quality variants (QUAL < 150) we retrieved about 700,000 high-quality SNVs were included in the analysis in this study. Exonic variants in genes of interest (Supplementary table 4) were selected for further analysis. Variants with less than 5 observations were removed. Of the 1,309 individuals with exome sequencing data, 1,100 had data on the metabolite levels. Single variant analyses were performed using GenABEL adjusting for relatedness.

REFERENCES

1. Wu PS, Otting G (2005) Rapid pulse length determination in high-resolution NMR. J Magn Reson 176: 115-119.
2. Vilen EM, Klinger M, Sandstrom C (2011) Application of diffusion-edited NMR spectroscopy for selective suppression of water signal in the determination of monomer composition in alginates. Magn Reson Chem.
3. Nicholson JK, Foxall PJ, Spraul M, Farrant RD, Lindon JC (1995) 750 MHz 1H and 1H-13C NMR spectroscopy of human blood plasma. Anal Chem 67: 793-811.
4. Almasy L, Blangero J (1998) Multipoint quantitative-trait linkage analysis in general pedigrees. Am J Hum Genet 62: 1198-1211.
5. Aulchenko YS, Ripke S, Isaacs A, van Duijn CM (2007) GenABEL: an R library for genome-wide association analysis. Bioinformatics 23: 1294-1296.
6. Li H, Durbin R (2009) Fast and accurate short read alignment with Burrows-Wheeler transform. Bioinformatics 25: 1754-1760.
7. Brouwer RW, van den Hout MC, Grosveld FG, van Ijcken WF NARWHAL, a primary analysis pipeline for NGS data. Bioinformatics 28: 284-285.
8. McKenna A, Hanna M, Banks E, Sivachenko A, Cibulskis K, et al. The Genome Analysis Toolkit: a MapReduce framework for analyzing next-generation DNA sequencing data. Genome Res 20: 1297-1303.
