# Supplementary material for: Insight in Genome-Wide Association of Metabolite Quantitative Traits by Exome Sequence Analyses
Source: PLoS Genet. 2015 Jan 8;11(1):e1004835. doi: 10.1371/journal.pgen.1004835 (PMC4287344; doi:10.1371/journal.pgen.1004835)
Supplement: S1 Table — Characteristics of ERF study sample. (PDF) [file pgen.1004835.s005.pdf]

Supplementary Table 1

Characteristics of ERF study sample

	**Mean**	**SD**	**min**	**max**
Age (years)	50.36	15.19	16.65	95.68
Total cholesterol (mmol/L)	5.55	1.08	1.80	9.20
HDL cholesterol (mmol/L)	1.27	0.36	0.20	3.10
LDL cholesterol (mmol/L)	3.72	0.97	1.00	7.10
Triglycerides (mmol/L)	1.33	0.73	0.00	6.90
Glucose (mmol/L)	4.67	1.12	1.90	15.90
Systolic blood pressure (mmHg)	140.05	20.26	85.50	239.00
Body mass index (kg/m^2)	26.87	4.65	15.54	61.80
	N	*%*		
Men	1,206	43.0		
Current smoking	1,011	39.0		
Type 2 diabetes	179	7.0		
Lipid lowering medication	329	12.7		
