# Supplementary material for: Insight in Genome-Wide Association of Metabolite Quantitative Traits by Exome Sequence Analyses
Source: PLoS Genet. 2015 Jan 8;11(1):e1004835. doi: 10.1371/journal.pgen.1004835 (PMC4287344; doi:10.1371/journal.pgen.1004835)
Supplement: S2 Table — Unique NMR Metabolite peaks selected for GWAS. In total we studied 42 uniquely annotated NMR peaks. (PDF) [file pgen.1004835.s006.pdf]

Supplementary Table 2. Unique NMR Metabolite peaks selected for GWAS. In total we studied 42 uniquely annotated NMR peaks

Metabolite	Chemical Shift
Lipids (CH3)	0.87
2-Hydroxybutyrate	0.89
Leucine	0.95
Isoleucine	1.00
Valine	1.03
3-Hydroxyisobutyrate	1.06
Alpha-ketoisovalerate	1.11
Ethanol	1.18
3-Hydroxybutyrate	1.19
Lipids (CH2)	1.27
Alanine	1.47
Acetate	1.91
L-Acetylcarnitine / Methionine	2.13
Acetone	2.22
Lipids (CH2CO)	2.22
Glutamate	2.35
Pyruvate	2.36
Oxaloacetate	2.39
Carnitine	2.41
Glutamine	2.46
Citrate	2.53
DMA	2.72
Dimethyl-glycine	2.92
Alpha-ketoglutarate	2.99
Lysine	3.02
Ornithine	3.05
TMAO / Betaine	3.25
1,5-Anhydrosorbitol	3.27
Methanol	3.36
Glycine	3.55
Glycerol	3.56
Myoinositol	3.62
Betaine	3.88
Creatine	3.92
Creatinine	4.04
Lactate	4.10
Proline	4.12
α-D-Glucose	5.23
Lipids (CH=CH*CH2CH2)	5.30
Tyrosine	6.89
Phenylalanine	7.42
Formate	8.45
