# Supplementary material for: Insight in Genome-Wide Association of Metabolite Quantitative Traits by Exome Sequence Analyses
Source: PLoS Genet. 2015 Jan 8;11(1):e1004835. doi: 10.1371/journal.pgen.1004835 (PMC4287344; doi:10.1371/journal.pgen.1004835)
Supplement: S4 Table — Correlation to risk factors of disease. (PDF) [file pgen.1004835.s008.pdf]

Supplementary Table 4 –A Correlation to risk factors of disease.

		Carnitine		Dimethyl-glycine		Proline		Pyruvate		Glycine		Lysine		Valine		3-Hydroxybutyrate	
		r	P-value	r	P-value	r	P-value	r	P-value	r	P-value	r	P-value	r	P-value	r	P-value
T2D	Cholesterol	0,05	1,10E-02	-0,01	4,97E-01	-0,05	1,17E-02	0,00	9,49E-01	0,01	7,14E-01	-0,11	***1,23E-07***	-0,01	5,89E-01	-0,04	3,73E-02
	LDL-cholesterol	0,02	2,96E-01	-0,02	4,41E-01	-0,06	5,44E-03	-0,04	6,41E-02	0,05	1,01E-02	-0,07	8,89E-04	-0,02	2,80E-01	-0,04	2,25E-02
	HDL-cholesterol	-0,04	8,93E-02	-0,02	4,44E-01	-0,13	***2,94E-10***	-0,13	***1,02E-10***	-0,01	6,61E-01	-0,14	***6,66E-12***	-0,24	***3,06E-32***	0,03	1,18E-01
	Triglycerides	0,13	***1,06E-10***	0,02	4,58E-01	0,12	***2,26E-09***	0,24	***1,33E-32***	-0,12	***8,50E-09***	0,00	8,71E-01	0,28	***1,07E-43***	-0,02	1,94E-01
	Glucose (fasting)	0,02	4,25E-01	-0,03	2,04E-01	0,04	5,64E-02	0,20	***1,63E-21***	-0,10	***6,03E-07***	0,13	***3,47E-10***	0,26	***1,20E-36***	0,04	8,77E-02
	Adiponectin	-0,09	3,07E-04	-0,03	1,65E-01	-0,12	***1,74E-06***	-0,14	***8,45E-09***	0,09	2,23E-04	-0,06	2,33E-02	-0,27	***1,39E-29***	0,06	9,54E-03
	HOMA insulin resistance	0,12	***1,31E-06***	0,06	1,53E-02	0,13	***3,02E-08***	0,27	***3,86E-29***	-0,14	***6,33E-09***	0,01	5,81E-01	0,30	***4,33E-36***	-0,03	1,09E-01
	Insulin	0,12	***1,76E-06***	0,06	1,48E-02	0,15	***8,54E-10***	0,26	***1,43E-27***	-0,15	***7,32E-10***	0,00	9,30E-01	0,30	***5,92E-37***	-0,05	1,47E-02
	Albumin	0,10	***5,21E-06***	-0,11	***2,41E-06***	-0,01	7,57E-01	-0,10	***1,50E-05***	0,03	1,69E-01	0,11	***2,97E-06***	-0,01	7,82E-01	-0,01	9,47E-01
	Leptin	0,14	***1,12E-08***	0,04	1,48E-01	0,10	***4,64E-05***	0,17	***1,35E-11***	-0,11	***2,21E-05***	0,06	1,67E-02	0,24	***4,94E-23***	-0,09	2,58E-04
	Resistin	-0,07	5,93E-03	0,15	***2,81E-09***	0,01	6,58E-01	0,07	7,73E-03	0,02	3,36E-01	-0,04	1,01E-01	-0,07	8,67E-03	0,01	9,01E-01
CVD	Heart rate	-0,03	3,29E-01	0,02	3,75E-01	0,03	2,51E-01	0,03	2,40E-01	-0,05	7,50E-02	-0,04	1,43E-01	0,07	6,00E-03	0,01	4,35E-01
	DBP	0,01	7,33E-01	-0,04	8,67E-02	0,04	7,04E-02	0,02	3,46E-01	-0,03	1,46E-01	-0,05	9,80E-03	0,11	***3,90E-07***	0,01	6,57E-01
	SBP	0,05	2,01E-02	0,00	9,61E-01	0,08	***8,11E-05***	0,05	1,43E-02	-0,05	1,61E-02	0,00	8,66E-01	0,14	***4,38E-11***	-0,01	8,25E-01
	Pulse wave velocity	0,02	4,09E-01	0,04	7,09E-02	0,04	4,55E-02	0,10	***8,92E-06***	-0,04	1,09E-01	0,01	7,77E-01	0,11	***3,35E-07***	0,01	8,24E-01
	QRS interval	-0,01	6,02E-01	0,00	9,12E-01	0,04	1,13E-01	-0,04	8,16E-02	0,01	7,94E-01	0,02	5,06E-01	0,06	2,00E-02	-0,01	7,68E-01
	QT interval	0,00	8,98E-01	0,01	8,25E-01	0,03	2,57E-01	-0,05	3,70E-02	-0,02	4,59E-01	0,01	5,67E-01	0,06	2,33E-02	-0,02	3,49E-01
	Intima-media thickness	0,01	7,99E-01	0,06	1,28E-02	-0,02	4,70E-01	-0,02	3,85E-01	-0,05	2,21E-02	0,07	4,90E-03	0,07	2,35E-03	0,03	2,32E-01
Inflammation	C-reactive protein	-0,05	2,85E-02	0,06	1,44E-02	-0,07	2,44E-03	0,06	1,65E-02	-0,11	***7,29E-06***	-0,12	***4,72E-07***	0,05	5,50E-02	0,08	7,47E-04
Kidney function	Creatine	0,01	7,08E-01	0,19	***3,02E-19***	0,02	4,81E-01	-0,03	2,56E-01	0,08	2,17E-04	0,03	1,56E-01	0,02	3,27E-01	0,03	1,02E-01
	eGFR	-0,02	3,11E-01	-0,14	***2,77E-10***	0,01	7,75E-01	0,05	2,25E-02	-0,09	***3,91E-05***	-0,04	5,16E-02	-0,03	2,30E-01	-0,03	6,91E-02
	Uric acid	0,28	***3,25E-16***	0,20	***1,20E-08***	0,14	***5,37E-05***	0,18	***1,17E-07***	-0,13	2,01E-04	0,12	4,01E-04	0,30	***1,39E-18***	-0,01	7,60E-01
	Creatinin	0,00	9,64E-01	0,22	***1,31E-22***	0,01	5,24E-01	-0,03	2,52E-01	0,08	2,35E-04	0,01	5,49E-01	0,01	6,23E-01	0,05	9,29E-03
Iron metabolism	Serum Iron levels	-0,02	6,41E-01	-0,04	2,72E-01	-0,12	9,89E-04	-0,09	7,61E-03	-0,01	7,44E-01	0,09	8,04E-03	0,00	9,20E-01	0,10	3,78E-03
	Ferritin levels	0,05	1,33E-01	0,03	4,85E-01	-0,07	5,06E-02	0,07	4,12E-02	-0,08	2,61E-02	0,08	2,12E-02	0,15	***2,26E-05***	-0,04	2,41E-01
	Transferrin saturation (%)	-0,03	4,21E-01	0,01	7,67E-01	-0,08	1,53E-02	-0,08	2,58E-02	0,03	3,54E-01	0,12	9,79E-04	-0,01	8,92E-01	0,08	1,61E-02
Osteoporosis	Lumbar-spine BMD	-0,03	2,09E-01	-0,06	1,54E-02	0,04	1,30E-01	0,03	2,48E-01	-0,11	***2,14E-05***	0,00	9,01E-01	0,00	9,65E-01	-0,01	8,12E-01
	Femural neck BMD	0,01	7,16E-01	-0,03	2,46E-01	0,03	1,88E-01	0,04	8,48E-02	-0,10	***1,36E-04***	0,07	8,65E-03	0,11	***9,15E-06***	-0,04	1,38E-01

Antropometry	Body-mass index	0,14	***4,40E-11***	0,01	5,84E-01	0,12	***2,80E-09***	0,24	***5,40E-32***	-0,18	***4,19E-18***	0,13	***1,45E-10***	0,38	***2,05E-82***	0,20	***1,85E-23***
	Waist-to-hip ratio	0,11	***7,32E-08***	0,04	4,30E-02	0,12	***5,10E-09***	0,23	***1,22E-29***	-0,15	***3,09E-12***	0,08	***1,56E-04***	0,33	***2,07E-57***	-0,05	1,88E-02
	Fat %	0,11	***3,29E-07***	0,01	5,13E-01	0,08	3,19E-04	0,18	***1,82E-17***	-0,15	***4,36E-12***	0,05	3,05E-02	0,29	***7,06E-44***	-0,06	3,53E-03
	Fat mass index	0,13	***4,23E-10***	0,02	3,01E-01	0,11	***5,16E-07***	0,21	***2,08E-23***	-0,16	***4,66E-14***	0,09	***1,10E-05***	0,34	***4,43E-61***	-0,06	3,83E-03
	Lean mass index	0,13	***3,86E-10***	0,00	8,62E-01	0,12	***2,75E-08***	0,16	***1,66E-14***	-0,12	***4,76E-08***	0,16	***7,44E-14***	0,31	***3,36E-51***	-0,12	***4,07E-08***
	Android fat	0,15	***5,09E-13***	0,02	2,60E-01	0,13	***7,91E-10***	0,24	***1,16E-28***	-0,17	***2,07E-15***	0,07	6,15E-04	0,37	***6,99E-72***	-0,09	***3,31E-05***
	Gynoid fat	0,11	***4,07E-07***	0,04	7,64E-02	0,10	***1,54E-06***	0,14	***2,17E-11***	-0,12	***6,90E-08***	0,09	***3,83E-05***	0,27	***4,90E-38***	-0,06	6,34E-03

Supplementary Table 4 –B

		Carnitine		Dimethyl-glycine		Proline		Pyruvate		Glycine		Lysine		Valine		3-hydoxybutyrate	
		r	P-value	r	P-value	r	P-value	r	P-value	r	P-value	r	P-value	r	P-value	r	P-value
T2D	Cholesterol	0,05	1,12E-02	-0,01	4,95E-01	-0,05	1,03E-02	0,00	9,90E-01	0,01	6,81E-01	-0,11	***8,16E-08***	-0,01	5,00E-01	-0,06	3,26E-03
	LDL-cholesterol	0,02	3,48E-01	-0,02	4,35E-01	-0,06	3,68E-03	-0,04	3,44E-02	0,06	5,64E-03	-0,07	5,29E-04	-0,03	1,29E-01	-0,07	1,03E-03
	HDL-cholesterol	0,00	9,99E-01	-0,01	5,18E-01	-0,10	***7,04E-07***	-0,08	2,49E-04	-0,06	4,95E-03	-0,11	***5,78E-08***	-0,16	***1,08E-14***	0,03	1,09E-01
	Triglycerides	0,11	***4,04E-07***	0,01	5,29E-01	0,10	***2,34E-06***	0,20	***1,01E-21***	-0,08	***1,15E-04***	-0,04	7,90E-02	0,21	***5,50E-25***	-0,03	1,66E-01
	Glucose (fasting)	-0,02	4,35E-01	-0,03	1,50E-01	0,01	5,97E-01	0,15	***9,42E-13***	-0,06	2,04E-03	0,10	***7,87E-07***	0,19	***1,27E-19***	0,06	6,67E-03
	Adiponectin	-0,05	2,41E-02	-0,03	1,89E-01	-0,09	2,99E-04	-0,08	6,90E-04	0,05	5,95E-02	-0,02	3,68E-01	-0,19	***2,16E-15***	0,10	***5,85E-05***
	HOMA insulin resistance	0,06	9,72E-03	0,06	1,30E-02	0,09	2,62E-04	0,18	***3,27E-14***	-0,07	5,24E-03	-0,05	2,93E-02	0,15	***5,00E-10***	-0,07	6,76E-03
	Insulin	0,06	1,51E-02	0,06	1,21E-02	0,10	***2,09E-05***	0,17	***1,40E-12***	-0,07	2,14E-03	-0,07	2,40E-03	0,15	***9,18E-10***	-0,07	5,28E-03
	Albumin	0,12	***1,22E-07***	-0,10	***2,76E-06***	0,01	7,79E-01	-0,07	1,01E-03	0,01	5,93E-01	0,12	***7,09E-08***	0,04	9,04E-02	-0,01	7,22E-01
	Leptin	0,11	***1,71E-05***	0,03	2,32E-01	0,04	1,15E-01	0,11	***2,44E-05***	0,00	9,47E-01	-0,03	1,87E-01	0,06	2,50E-02	-0,08	2,27E-03
	Resistin	-0,08	1,91E-03	0,12	***5,79E-06***	0,01	8,16E-01	0,04	1,79E-01	0,04	1,45E-01	-0,02	5,63E-01	-0,08	2,54E-03	-0,02	3,91E-01
CVD	Heart rate	-0,03	2,32E-01	0,02	3,84E-01	0,02	3,33E-01	0,02	4,05E-01	-0,04	1,25E-01	-0,04	9,26E-02	0,06	1,93E-02	0,02	4,31E-01
	DBP	-0,02	3,89E-01	-0,04	6,58E-02	0,02	4,48E-01	-0,02	2,30E-01	0,00	9,21E-01	-0,08	***1,30E-04***	0,04	5,73E-02	0,02	2,79E-01
	SBP	0,03	2,06E-01	0,00	9,66E-01	0,06	2,55E-03	0,01	5,83E-01	-0,02	3,13E-01	-0,02	3,64E-01	0,08	***1,24E-04***	0,00	8,41E-01
	Pulse wave velocity	0,01	6,33E-01	0,04	7,52E-02	0,04	9,00E-02	0,09	***8,38E-05***	-0,03	2,46E-01	0,00	9,51E-01	0,10	***8,88E-06***	0,05	3,95E-02
	QRS interval	-0,02	4,08E-01	0,00	8,92E-01	0,03	1,84E-01	-0,06	1,93E-02	0,02	5,07E-01	0,01	7,04E-01	0,04	1,07E-01	0,00	9,48E-01
	QT interval	0,00	8,70E-01	0,01	8,27E-01	0,03	2,66E-01	-0,06	2,72E-02	-0,02	4,79E-01	0,01	5,86E-01	0,06	1,87E-02	-0,02	4,65E-01
	Intima-media thickness	-0,01	7,40E-01	0,06	1,42E-02	-0,03	2,06E-01	-0,05	4,93E-02	-0,04	1,20E-01	0,05	2,29E-02	0,03	1,29E-01	0,04	6,97E-02
Inflammation	C-reactive protein	-0,06	1,02E-02	0,06	1,54E-02	-0,08	7,30E-04	0,04	6,65E-02	-0,10	***4,21E-05***	-0,13	***5,38E-08***	0,02	3,12E-01	0,08	1,77E-03
Kidney function	Creatine	0,01	7,28E-01	0,19	***3,13E-19***	0,01	4,95E-01	-0,03	2,20E-01	0,08	***1,46E-04***	0,03	1,61E-01	0,02	3,32E-01	0,02	4,03E-01
	eGFR	-0,03	2,38E-01	-0,14	***2,53E-10***	0,00	8,83E-01	0,04	3,85E-02	-0,09	***7,07E-05***	-0,05	3,44E-02	-0,04	7,69E-02	-0,02	3,81E-01

	Uric acid	0,25	***3,93E-13***	0,21	***2,42E-09***	0,10	2,70E-03	0,11	1,81E-03	-0,07	3,86E-02	0,08	1,81E-02	0,19	***3,47E-08***	-0,06	7,39E-02
	Creatinine	0,00	9,73E-01	0,22	***1,35E-22***	0,01	5,27E-01	-0,03	2,31E-01	0,08	1,76E-04	0,01	5,52E-01	0,01	6,17E-01	0,03	1,77E-01
Iron metabolism	Serum Iron levels	0,00	9,10E-01	-0,04	2,84E-01	-0,10	2,66E-03	-0,07	3,51E-02	-0,03	4,19E-01	0,11	2,40E-03	0,04	2,31E-01	0,10	5,61E-03
	Ferritin levels	0,07	4,50E-02	0,00	9,99E-01	-0,06	9,80E-02	0,03	4,26E-01	-0,06	1,06E-01	0,13	2,18E-04	0,19	***4,72E-08***	-0,03	4,12E-01
	Transferrin saturation (%)	-0,01	7,06E-01	0,01	7,38E-01	-0,07	3,94E-02	-0,05	1,30E-01	0,01	7,13E-01	0,13	***1,59E-04***	0,04	2,38E-01	0,08	2,43E-02
Osteoporosis	Lumbar-spine BMD	-0,06	2,51E-02	-0,06	1,11E-02	0,02	4,88E-01	-0,01	6,13E-01	-0,08	1,80E-03	-0,02	4,26E-01	-0,07	4,69E-03	0,00	8,98E-01
	Femural neck BMD	-0,03	2,22E-01	-0,03	1,79E-01	0,00	9,49E-01	-0,03	2,96E-01	-0,05	5,55E-02	0,03	2,31E-01	0,00	8,89E-01	-0,02	3,55E-01
Antropometry	Body-mass index	na	***na***	na	na	na	***na***	na	***na***	na	***na***	na	***na***	na	***na***	na	***na***
	Waist-to-hip ratio	0,05	1,52E-02	0,04	4,21E-02	0,07	8,48E-04	0,13	***1,61E-10***	-0,06	2,02E-03	0,01	5,07E-01	0,16	***2,86E-15***	-0,03	2,20E-01
	Fat %	0,01	8,13E-01	0,01	7,00E-01	-0,03	1,62E-01	-0,01	6,12E-01	-0,01	5,14E-01	-0,09	***1,89E-05***	-0,01	5,47E-01	-0,03	1,19E-01
	Fat mass index	0,02	3,93E-01	0,03	1,30E-01	-0,02	3,46E-01	-0,04	8,01E-02	0,02	4,45E-01	-0,08	***1,04E-04***	-0,05	3,55E-02	-0,03	1,10E-01
	Lean mass index	0,05	2,53E-02	-0,01	7,31E-01	0,04	6,00E-02	-0,03	1,94E-01	0,03	2,01E-01	0,09	***1,81E-05***	0,04	4,83E-02	-0,12	***3,72E-08***
	Android fat	0,07	6,37E-04	0,03	1,42E-01	0,05	2,39E-02	0,05	3,43E-02	-0,02	3,20E-01	-0,10	***1,78E-06***	0,07	1,75E-03	-0,09	***1,55E-05***
	Gynoid fat	-0,01	6,21E-01	0,05	1,47E-02	0,00	9,92E-01	-0,11	***1,91E-07***	0,06	2,81E-03	-0,04	4,86E-02	-0,10	***3,09E-06***	-0,03	2,38E-01

Selected NMR variables with significant and suggestively significant P-values have been selected to investigate their relation to markers of disease, adjusted by age and gender effects. Bold italic numbers show the P values that pass the Bonferroni significance threshold (P-value < 0.000179) for the number of tests (35 × 8=280) performed. HOMA; homeostatic model assessment, eGFR; estimated Glomerular Filtration Rate. Supplementary Table 4B shows the BMI adjusted partial correlation results.
