# Supplementary material for: Insight in Genome-Wide Association of Metabolite Quantitative Traits by Exome Sequence Analyses
Source: PLoS Genet. 2015 Jan 8;11(1):e1004835. doi: 10.1371/journal.pgen.1004835 (PMC4287344; doi:10.1371/journal.pgen.1004835)
Supplement: S5 Table — Association between metabolites and mQTL adjusted by BMI. (PDF) [file pgen.1004835.s009.pdf]

Supplementary Table 5. Association between metabolites and mQTL adjusted by BMI.

Metabolite	SNP	P-value	P-value BMI adjusted	MAF	Location	CHR	Position
Glycine	rs715	1.27×10^{-32}	1.12×10^{-31}	0.34	3'UTR	2	211251300
Proline	rs2540641	1.11×10^{-19}	2.38×10^{-20}	0.09	33 KB from *PRODH*	22	17339684
Dimethyl-glycine[Novel]	rs248386	1.65×10^{-19}	1.32×10^{-18}	0.15	intronic	5	78365983
Carnitine	rs1171614	4.81×10^{-14}	3.31×10^{-16}	0.18	5'UTR	10	61139544
Pyruvate[Novel]	rs1922005	1.26×10^{-8}	2.39×10^{-8}	0.13	intronic	2	217441741
3-Hydroxybutyrate[Novel]	rs9896573	1.65×10^{-8}	3.19×10^{-8}	0.09	6 KB from *KCNJ16*	17	65650639
Lysine	rs8056893	2.14×10^{-8}	1.31×10^{-8}	0.28	intronic	16	66861893
Valine[Novel]	rs11687765	3.49×10^{-8}	6.20×10^{-7}	0.44	intergenic	2	82179042
