# Supplementary material for: Insight in Genome-Wide Association of Metabolite Quantitative Traits by Exome Sequence Analyses
Source: PLoS Genet. 2015 Jan 8;11(1):e1004835. doi: 10.1371/journal.pgen.1004835 (PMC4287344; doi:10.1371/journal.pgen.1004835)
Supplement: S6 Table — The effect of age and gender on metabolite levels. (PDF) [file pgen.1004835.s010.pdf]

Supplementary Table 5. The effects of age and gender on metabolite levels.

	**Age**			**Sex**		
	**R^2**	**Beta**	**P-value**	**R^2**	**Beta (men)**	**P-value**
Lipids (CH3)	3.01	0.17	2.02E-19	4.65	0.22	3.34E-29
Hydroxybutyrate_2	1.85	0.14	1.56E-12	1.51	0.12	1.67E-10
Leucine	1.12	0.11	3.18E-08	21.16	0.46	1.85E-138
Isoleucine	1.44	0.12	4.25E-10	21.55	0.46	6.78E-141
Valine	2.34	0.15	1.62E-15	15.66	0.40	8.65E-100
3-Hydroxyisobutyrate	0.06	0.03	1.13E-01	3.10	0.18	5.92E-20
Alpha-ketoisovalerate	0.23	-0.05	8.25E-03	3.22	0.18	9.54E-21
Ethanol	0.47	-0.07	2.59E-04	0.21	0.05	1.05E-02
3-Hydroxybutyrate	0	0.01	4.91E-01	0.07	-0.03	9.84E-02
Lipids (CH2)	2.67	0.16	2.46E-17	3.88	0.20	1.49E-24
Alanine	6.01	0.25	1.22E-37	0.19	0.05	1.48E-02
Acetate	0.32	-0.06	2.19E-03	0.48	0.07	2.27E-04
Methionine	1.17	0.11	1.56E-08	7.29	0.27	1.85E-45
Acetone	2.20	0.15	1.25E-14	6.06	0.25	9.34E-38
Lipids (CH2CO)	1.86	0.14	1.40E-12	3.42	0.19	9.00E-22
Glutamate	3.89	0.20	1.30E-24	4.04	0.20	1.68E-25
Pyruvate/Oxaloacetate	0.17	0.05	2.06E-02	0.39	-0.07	8.33E-04
Succinate	8.57	0.29	2.74E-53	0.00	0.01	6.77E-01
Carnitine	5.45	0.23	3.99E-34	8.62	0.29	9.68E-54
Glutamine	6.40	0.25	5.62E-40	2.89	0.17	8.78E-19
Citrate	10.09	0.32	3.72E-63	0.21	-0.05	1.11E-02
Dimethylamine	12.20	0.35	1.71E-76	2.63	0.16	3.51E-17
Dimethylglycine	4.86	0.22	1.68E-30	2.18	0.15	1.65E-14
Alpha-ketoglutarate	2.63	0.16	3.85E-17	3.47	0.19	3.70E-22
Lysine	10.39	0.32	4.46E-65	2.89	0.17	9.18E-19
Ornithine	22.52	0.47	5.99E-148	1.53	0.13	1.20E-10
TMAO_Betaine	5.85	0.24	1.80E-36	15.11	0.39	1.06E-95
1.5-Anhydrosorbitol	17.84	0.42	1.38E-114	0.66	0.08	1.78E-05
Methanol	2.14	-0.15	2.79E-14	0.69	0.09	1.11E-05
Glycine	1.44	0.12	4.67E-10	2.58	-0.16	8.30E-17
Glycerol	2.94	0.17	4.82E-19	8.72	-0.30	2.69E-54
Myoinositol	21.36	0.46	1.95E-139	0.76	0.09	4.49E-06
Betaine	8.95	0.30	9.31E-56	13.86	0.37	1.57E-87
Creatine	0.19	0.05	1.36E-02	16.79	-0.41	1.68E-107
Creatinine	1.30	0.12	2.80E-09	25.19	0.50	8.23E-168
Lactate	3.71	0.19	1.43E-23	1.78	0.13	4.18E-12
Proline	4.94	0.22	5.98E-31	8.54	0.29	4.66E-53
Glucose	11.15	0.33	2.45E-69	2.35	0.15	1.89E-15
Lipids (CH=CH*CH2CH2)	2.45	0.16	4.99E-16	4.28	0.21	6.01E-27
Tyrosine	11.60	0.34	8.82E-73	3.98	0.20	2.92E-25
Phenylalanine	11.85	0.34	1.65E-74	0.00	-0.02	4.28E-01
Formate	0.16	0.04	2.18E-02	0.02	0.02	2.20E-01
